# Transcriptomics Changes in the Peritoneum of Mice with Lipopolysaccharide-Induced Peritonitis

**DOI:** 10.3390/ijms222313008

**Published:** 2021-11-30

**Authors:** Shaoguang Liu, Shaotong Zhang, Yulong Sun, Wence Zhou

**Affiliations:** 1 The First Clinical Medical College, Lanzhou University, Lanzhou 730000, China; liushaoguang@gszy.edu.cn; 2 The Clinical Medical College, Ningxia Medical University, Yinchuan 750004, China; zhangshaotong@nxmu.edu.cn; 3 Key Laboratory for Space Biosciences & Biotechnology, Institute of Special Environmental Biophysics, School of Life Sciences, Northwestern Polytechnical University, Xi’an 710072, China; yulongsun@nwpu.edu.cn

**Keywords:** peritonitis, LPS, transcriptomic profiles, RNA sequencing, bioinformatics, molecular dynamics

## Abstract

Peritonitis caused by LPS is a severe clinical challenge, which causes organ damage and death. However, the mechanism of LPS-induced peritonitis has not been fully revealed yet. Here, we investigated the transcriptome profile of the peritoneal tissue of LPS-induced peritonitis in mice. A model of LPS-induced peritonitis in mice was established (LPS 10 mg/kg, i.p.), and the influence of TAK 242 (TLR4 inhibitor) on the level of inflammatory cytokines in mouse peritoneal lavage fluid was investigated by using an ELISA test. Next, the peritoneal tissues of the three groups of mice (Control, LPS, and LPS+TAK 242) (*n* = 6) were isolated and subjected to RNA-seq, followed by a series of bioinformatics analyses, including differentially expressed genes (DEGs), enrichment pathway, protein-protein interaction, and transcription factor pathway. Then, qPCR verified-hub genes that may interact with TAK 242 were obtained. Subsequently, the three-dimensional structure of hub proteins was obtained by using homology modeling and molecular dynamics optimization (300 ns). Finally, the virtual docking between TAK 242 and hub proteins was analyzed. Our results showed that TAK 242 significantly inhibited the production of inflammatory cytokines in the peritoneal lavage fluid of mice with peritonitis, including IL-6, IFN-γ, IL-1β, NO, and TNF-α. Compared with the Control group, LPS treatment induced 4201 DEGs (2442 down-regulated DEGs and 1759 up-regulated DEGs). Compared with the LPS group, 30 DEGs were affected by TAK 242 (8 down-regulated DEGs and 22 up-regulated DEGs). A total of 10 TAK 242-triggered hub genes were obtained, and the possible docking modes between TAK 242 and hub proteins were acquired. Overall, our data demonstrated that a large number of DEGs were affected in LPS-triggered peritonitis mice. Moreover, the TLR4 inhibitor TAK 242 is capable of suppressing the inflammatory response of LPS-induced peritonitis. Our work provides clues for understanding the pathogenesis of LPS-induced peritonitis in mice.

## 1. Introduction

Peritonitis remains a severe clinically challenging problem that brings a heavy burden to the health system. At present, the primary treatment strategy for peritonitis is antibiotic therapy combined with surgical intervention to eliminate the causes. Although a series of treatment progressions have been achieved, the mortality rate caused by peritonitis and its complications is still rising [1]. Based on the cause of its occurrence, peritonitis can be divided into aseptic peritonitis, bacterial peritonitis, peritoneal dialysis-related peritonitis, and LPS (lipopolysaccharide, LPS)-induced peritonitis [2,3,4].

Injecting LPS into the abdominal cavity of mice can cause peritonitis and a significant increase in cytokines in mice, including IL-6, IFN-γ, IL-1β, nitric oxide (NO), and TNF-α [5,6,7,8,9]. An increasing number of therapeutic molecules have been found to be effective in attenuating LPS-mediated peritonitis. (1) For the protein and peptide molecules, LPS-binding protein (LBP) [10], IRW (Ile-Arg-Trp) [11], and Cytochrome P450 1A1 (CYP1A1) [12] can significantly inhibit LPS-mediated peritonitis in mice. LBP suppresses the levels of serum TNF-α and IL-6 in LPS-treated mice peritonitis [10]. CYP1A1 attenuates LPS-induced mouse peritonitis via stimulating M2 macrophages [12]. (2) For the natural products, a series of molecules isolated from plants have shown anti-inflammatory activity in mouse models of LPS-caused peritonitis, including Ba-ME [13], Kaurenoid acid (KA) [14], Epimedium brevicornu Maxim ethanol extract (EBME) [15], and Ilexgenin A [5]. KA inhibits a series of index in LPS-induced peritonitis in mice, such as TNF-α, IL-33, and IL-1β [14]. (3) Certain physical stimuli also suppress the inflammatory reaction of LPS-induced peritonitis in mice, such as artificial sunlight [16] and low-level laser therapy [17]. Very recently, DUSP11 (dual-specificity phosphatase 11, DUSP11) is reported to inhibit LPS-induced peritonitis in mice. Compared with wild-type mice, the LPS-triggered serum production of cytokines (TNF-α, IL-1β, and IL-6) in DUSP11-deficient mice were significantly elevated [6].

Toll-like receptor 4 (also known as CD284 [cluster of differentiation 284]) is a transmembrane protein, which belongs to the pattern recognition receptor (PRR) family [18]. One of the common agonists of TLR4 is LPS, which is a component of selected Gram-positive bacteria and many Gram-negative bacteria [19]. The binding of LPS to TLR4 leads to the activation of a series of downstream signaling pathways, which play a fundamental role in various physiological processes [18,19]. TAK 242 (resatorvid) is a TLR4 inhibitor [20,21,22,23,24,25], which can suppress a series of cytological events in inflammation, including inflammatory cytokine production, inflammatory gene expression, and inflammation-related pathways activation [20,23]. However, to the best of our knowledge, the effect of TLR4 (Toll-like receptor 4) inhibitor on the transcriptomics of LPS-mediated mouse peritonitis has not been reported yet.

So far, the pathogenesis of LPS-mediated peritonitis has not been fully elucidated [1,2]. In the process of searching for clues to LPS-mediated peritonitis, transcriptomics may be an effective tool, which can be used to screen for promising candidate genes.

**Figure 1:**
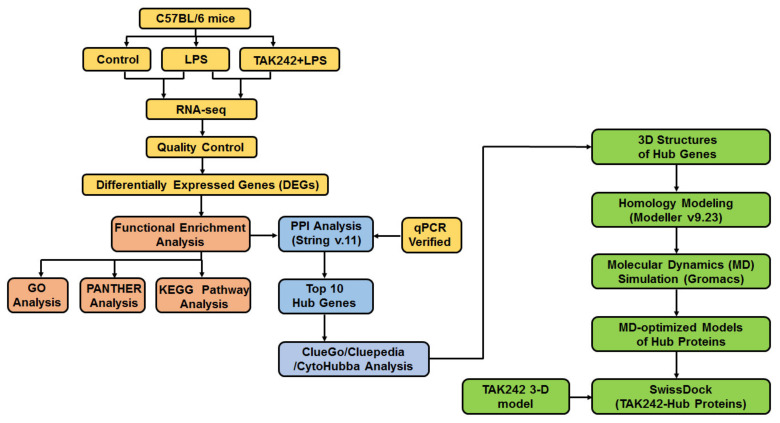
Outline of this study.

In the present study, the transcriptomics profiles of mouse peritoneum with LPS-mediated peritonitis were investigated with a series of comprehensive approaches such as transcriptomics, bioinformatics, and molecular simulation (Figure 1). (1) The establishment of the model. An LPS-stimulated mouse peritonitis model was established. (2) Anti-peritonitis activity of TLR4 inhibitor TAK 242. The effect of TAK 242 on the inflammatory cytokines in the peritoneal lavage fluid of mice was examined by the ELISA test. (3) Transcriptome sequencing of mouse peritoneal tissue. The peritoneal tissues of three groups of mice (Control group, LPS group, and LPS+TAK 242 group) (*n* = 6) were isolated and subjected to RNA-seq. (4) Bioinformatics analysis. A series of bioinformatics analyses were performed, including differentially expressed genes (DEGs), enrichment pathways, protein-protein interaction, transcription factor pathways, and hub genes. (5) Molecular dynamics analysis. The three-dimensional structure of hub proteins corresponding to hub genes was obtained by using homology modeling and molecular dynamics (300 ns). (6) Molecular docking. The molecular docking between TAK 242 and hub proteins was carried out to investigate the possible binding modes. Overall, our data provide clues for gene expression in mouse models of LPS-mediated peritonitis, which will be helpful for exploring the mechanism of LPS-induced peritonitis in mice.

## 2. Results

### 2.1. LPS-Induced Peritonitis in Mice

**Figure 2:**
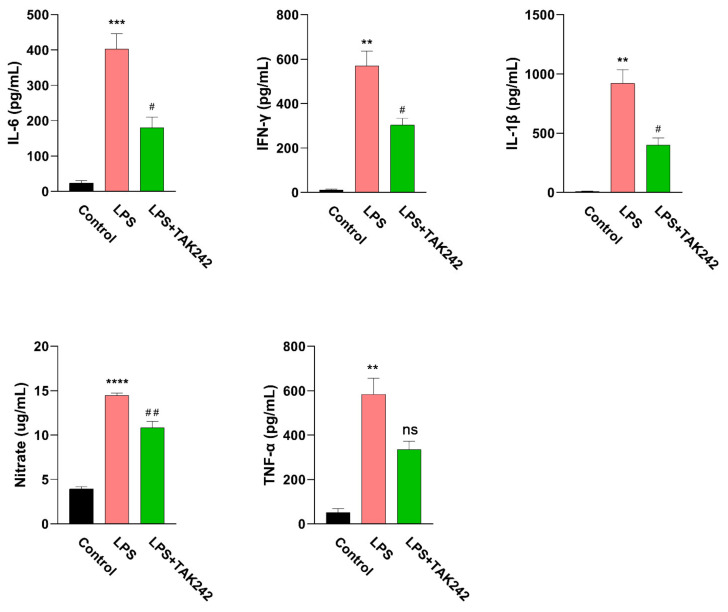
The changes of cytokine levels in LPS-induced peritonitis mice. C57BL/6 mice were injected with LPS (10 mg/kg, i.p.) with or without TAK 242 (5 mg/kg, i.p.). Six hours later, saline (2 mL/mouse) was injected intraperitoneally into the abdominal cavity of the mouse before the mouse was sacrificed. Then, peritoneal lavage fluids were collected and subjected to ELISA assay for a series of cytokines including IL-6, IFN-γ, IL-1β, Nitric oxide, and TNF-α. The results were demonstrated as means ± S.E.M. *, significantly different from the control group; ** *p* < 0.01; *** *p* < 0.001; **** *p* < 0.0001. #, significantly different from the LPS group; #, *p* < 0.05; ##, *p* < 0.01; and ns, no significance. Statistical significance interpretation was carried out by using the *t*-test approach. The data were demonstrated as the means ± S.E.M. Statistical significance interpretation was carried out using the one-way ANOVA followed by the Tukey post hoc tests.

The mouse peritonitis model is constructed by intraperitoneal injection of LPS. The cytokines in the peritoneal lavage fluid of the LPS-treated mice were significantly higher than those in the control group (Figure 2), as TNF α 47.09 ± 13.01 pg/mL vs. 546.7 ± 156.0 pg/mL (LPS vs. control), IL-1 β 8.345 ± 2.746 pg/mL vs. 921.7 ± 114.9 pg/mL (LPS vs. control), IL-6 23.89 ± 6.485 pg/mL vs. 403.4 ± 42.08 pg/mL (LPS vs. control), IFN-γ 11.61 ± 4.252 pg/mL vs. 570.6 ± 65.84 pg/mL (LPS vs. control), and nitric oxide 3.942 ± 0.2423 μg/mL vs. 14.47 ± 0.2477 μg/mL (LPS vs. control).

Subsequently, the effect of TLR4 inhibitor TAK 242 on the production of inflammatory cytokines in the peritoneal lavage fluid of LPS-induced peritonitis mice was tested. Compared with the LPS treatment group, TAK 242 significantly inhibited the levels of inflammatory mediators in the mouse peritoneal lavage fluid, as TNF α 546.7 ± 156.0 pg/mL vs. 312.6 ± 73.53 pg/mL (TAK 242 vs. LPS), IL-1 β 921.7 ± 114.9 pg/mL vs. 400.2 ± 59.19 pg/mL (TAK 242 vs. LPS), IL-6 403.4 ± 42.08 pg/mL vs. 180.2 ± 30.09 pg/mL (TAK 242 vs. LPS), IFN-γ 570.6 ± 65.84 pg/mL vs. 303.4 ± 30.20 pg/mL (TAK 242 vs. LPS), and nitric oxide 14.47 ± 0.2477 μg/mL vs. 10.81 ± 0.7219 μg/mL (TAK 242 vs. LPS) (Figure 2).

### 2.2. Screening of DEGs

**Figure 3:**
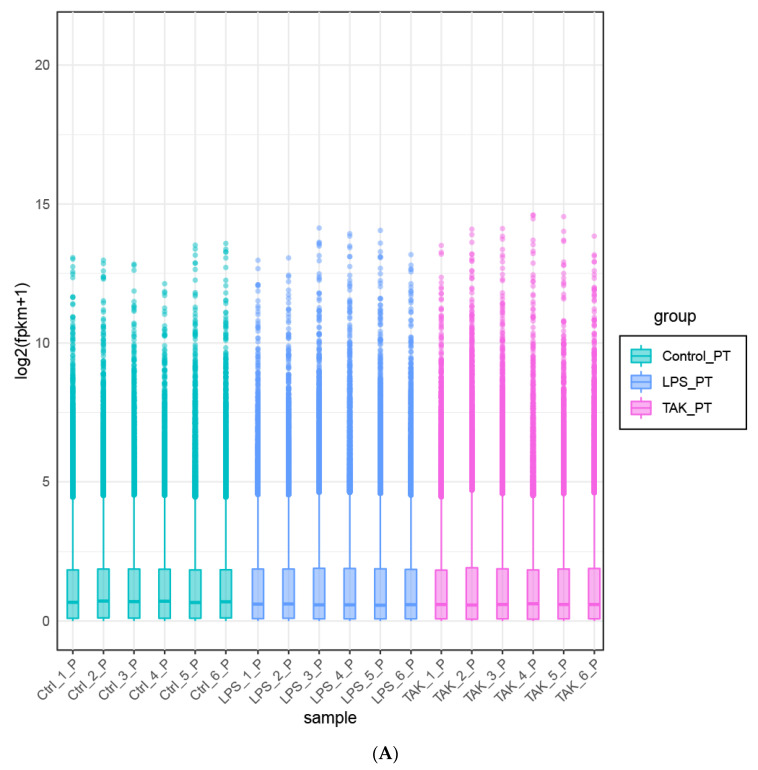

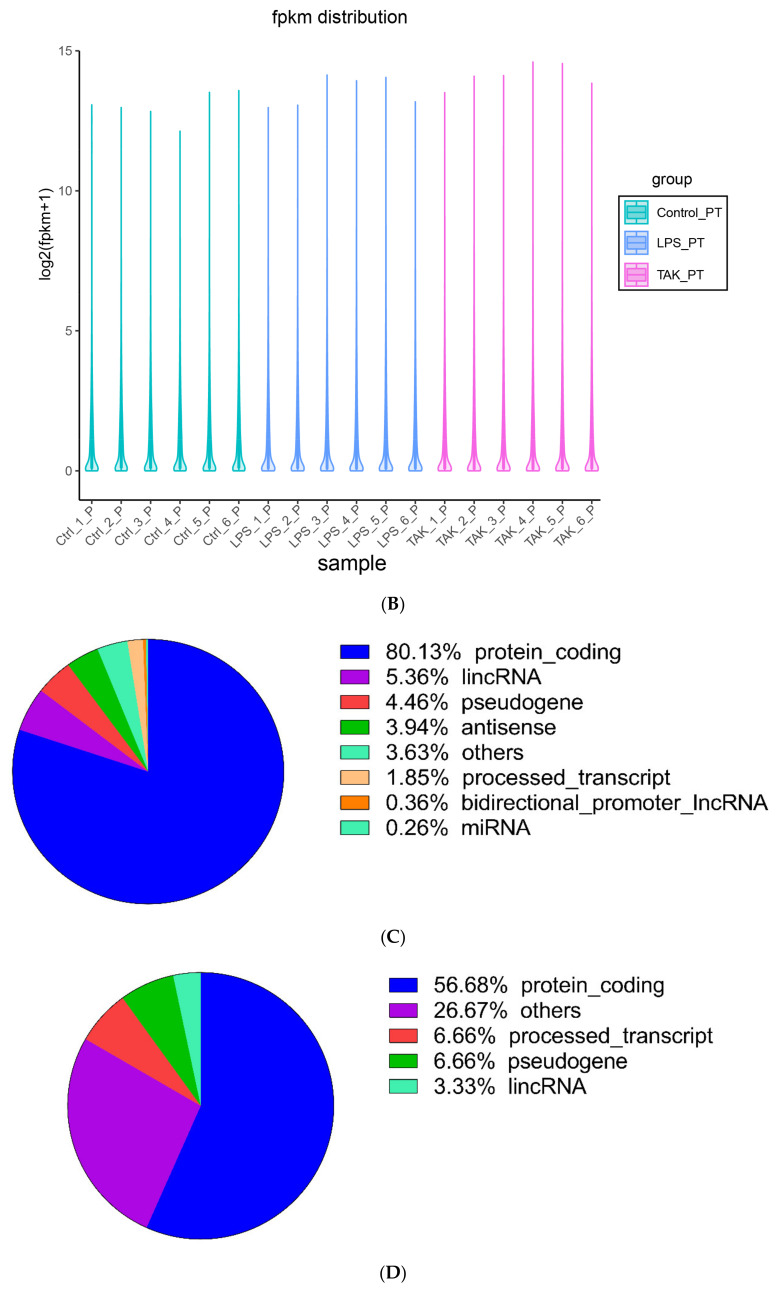

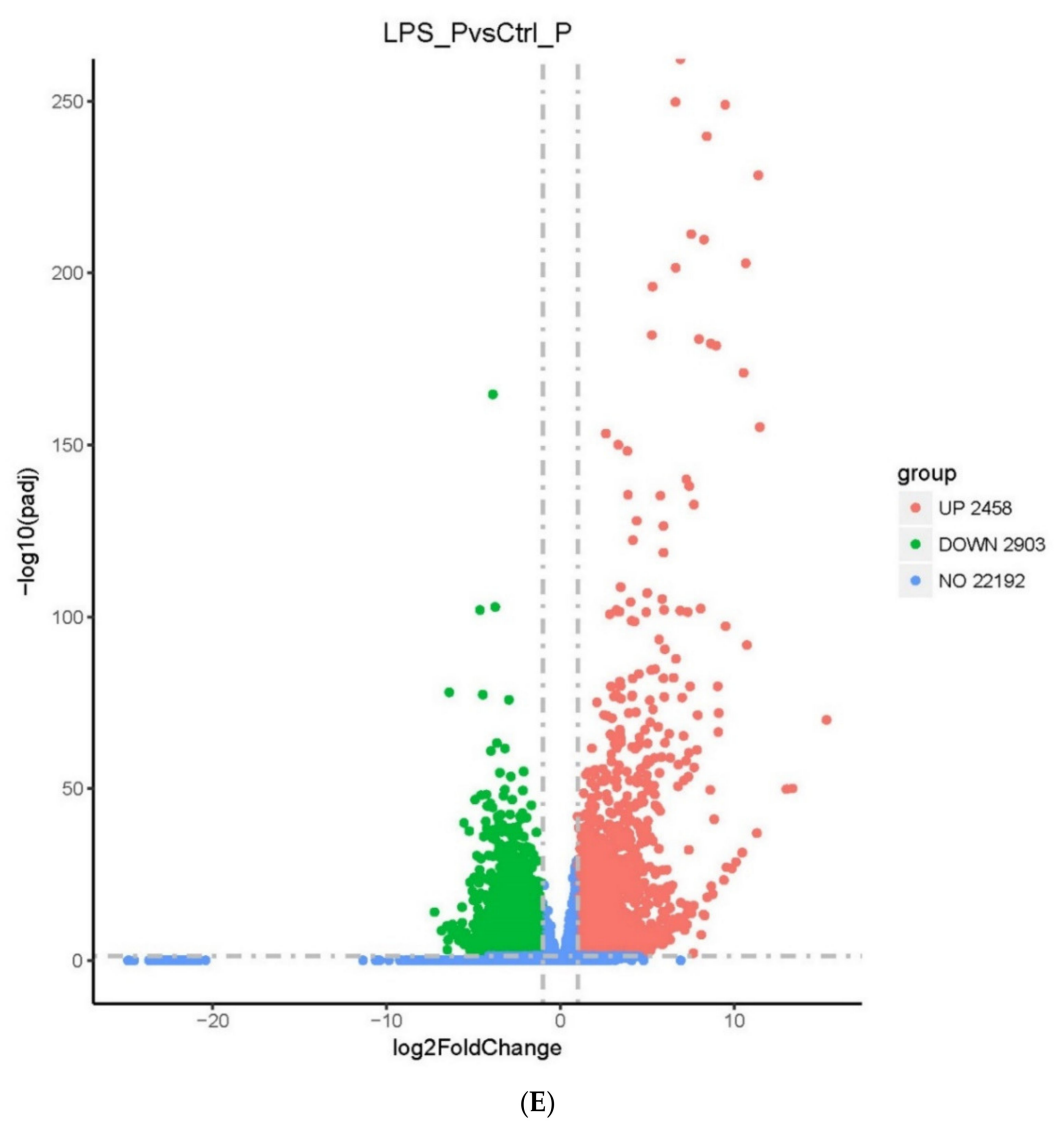

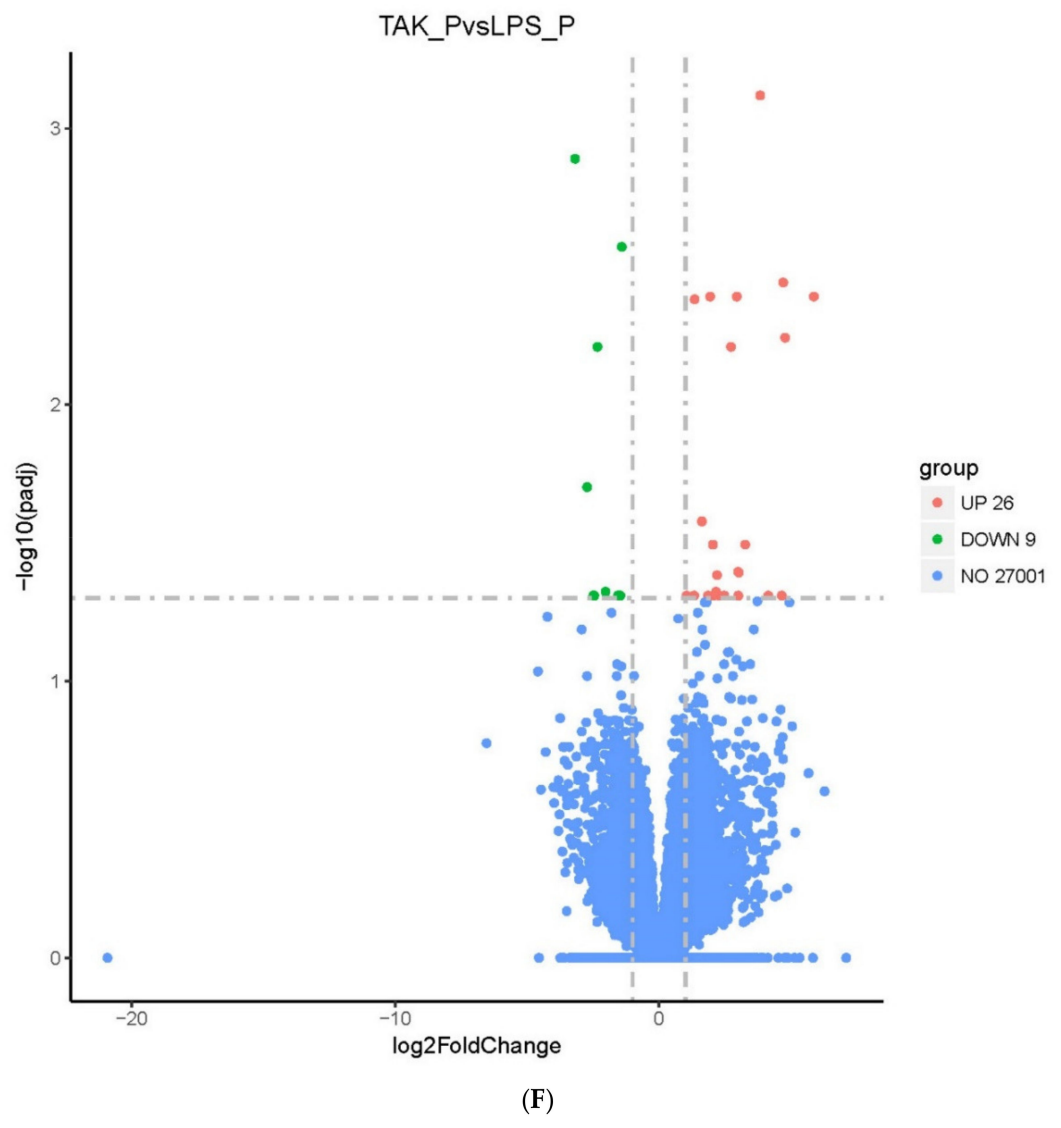

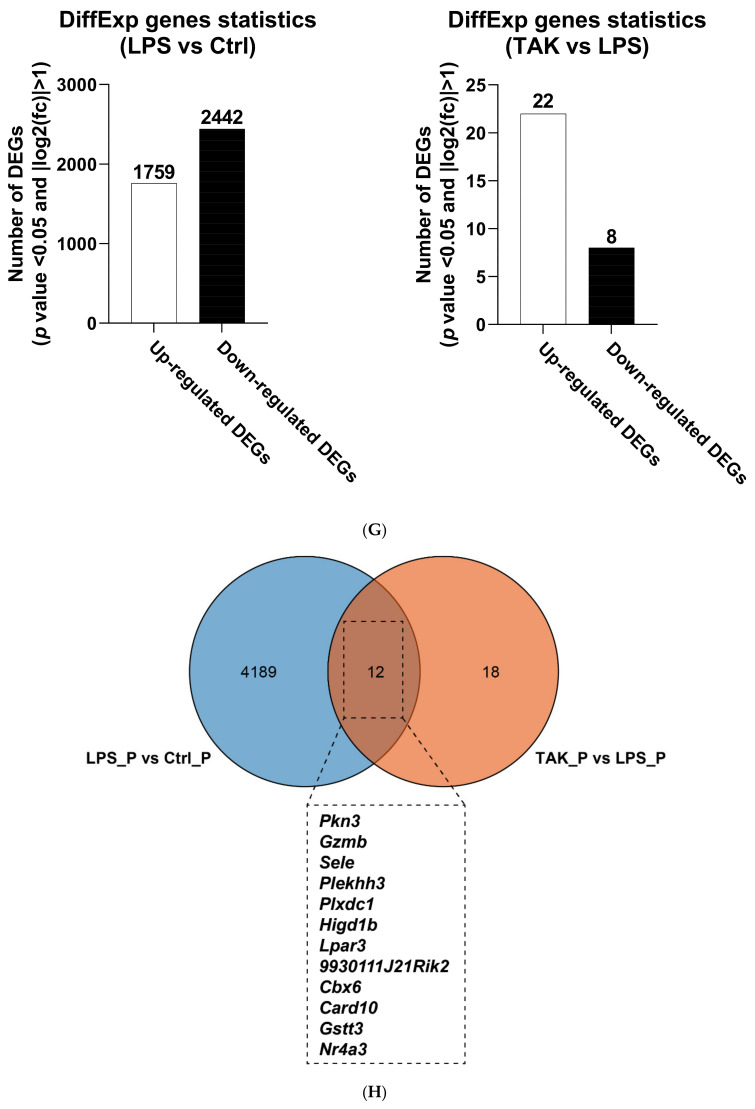

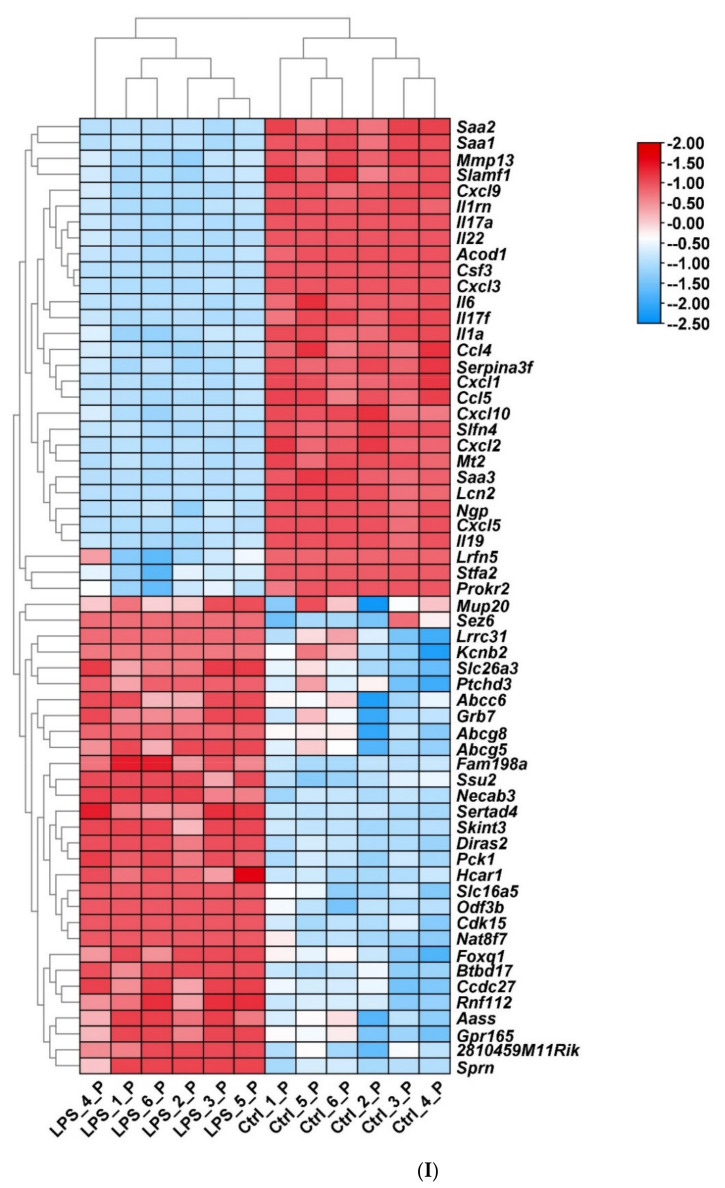

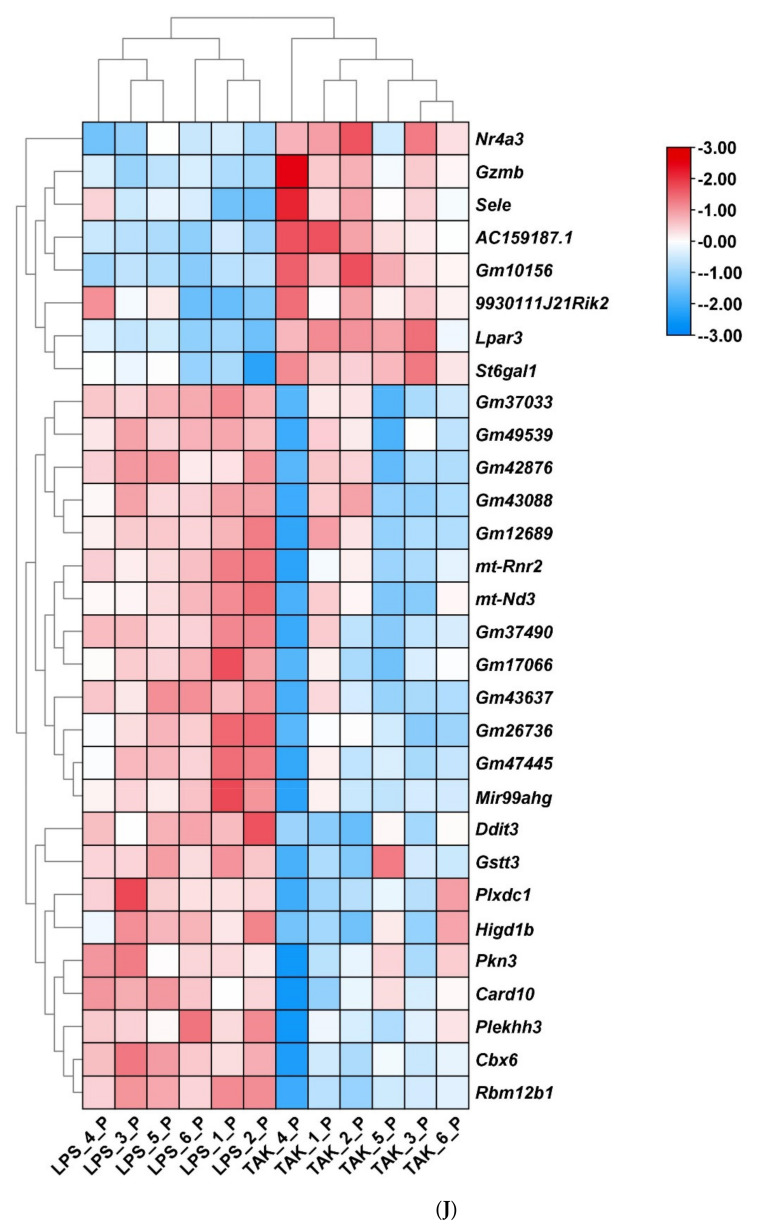
Basic information of RNA-seq detection. (**A**,**B**) The boxplot (**A**) and (**B**) Violin plot gene expression. (**C**,**D**) The proportion of the differentiated expressed genes (DEGs) in group 1 (LPS vs. Control) (**C**) and group 2 (TAK vs. LPS) (**D**). (**E**,**F**) Volcano plots of RNA-seq results in group 1 (LPS vs. Control) (**E**) and group 2 (TAK vs. LPS) (**F**) (green dots, down-regulated genes; red dots, up-regulated genes). (**G**) Statistics of DEGs (those genes that cannot be annotated were removed). (**H**) Venn plot of DEGs. (**I**,**J**) Heat map of the DEGs in group 1 (LPS vs. Control) (**I**) and group 2 (TAK vs. LPS) (**J**). In addition, the 30 genes with the most significant changes in the up-regulated and down-regulated groups were shown separately.

After the LPS-induced peritonitis model in mice was established, the peritoneal tissues of the mice were sent to RNA-seq testing (Figure 3A,C,E,G,I). According to the screening criteria (log2(fc) > 1 and *p*-value < 0.05), a total of 4201 DEGs were obtained, including 2442 down-regulated DEGs and 1759 up-regulated DEGs (Figure 3G and Supplementary_1). In contrast to Figure 3E, only the fully annotated genes were shown in Figure 3G. The distribution of each group of genes was presented with a boxplot, a volcano plot, and a violin plot (Figure 3A,E,I). LPS caused changes in the expression of multiple genes in the mouse peritoneum, including protein_coding: 80.09%, lincRNA: 5.36%, pseudogene: 4.46%, antisense: 3.94%, others: 3.63%, processed_transcript: 1.85%, bidirectional_promoter_lncRNA: 0.36%, and miRNA: 0.26% (Figure 3C). Overall, LPS suppressed more genes (2442 down-regulated genes) than stimulating genes (1759 up-regulated genes) on the transcriptional level of the peritoneum (Supplementary Tables S1, S3, S4, S6, S8, S10 and S11).

After TAK 242 injection, a total of 30 DEGs were acquired, including eight down-regulated DEGs and 22 up-regulated DEGs (Figure 3G and Supplementary_1). TAK 242 treatment resulted in changes in the expression of many types of genes, including protein_coding: 56.66%, others: 26.66%, processed_transcript: 6.66%, pseudogene: 6.66%, and lincRNA: 33% (Figure 3D). Compared with the LPS treatment group, the genetic changes caused by TAK 242 were shown by a boxplot, a volcano plot, and a violin plot (Figure 3B,D,F,J). TAK 242 stimulated more genes (22 up-regulated genes) than inhibiting genes (8 down-regulated genes) on the transcriptional level of the peritoneum (Supplementary Tables S2, S5, S7 and S9).

As shown in Figure 3H, a total of 12 common DEGs were obtained both in group 1 (LPS vs. Control) (E) and group 2 (TAK vs. LPS), including *Pkn3, Gzmb, Sele, Plekhh3, Plxdc1, Higd1b, Lpar3, 9930111J21Rik2, Cbx6, Card10, Gstt3*, and *Nr4a3.*

### 2.3. Enrichment Pathway of DEGs-KEGG

In order to investigate the biological pathways represented by DEGs, a series of tools were used to explore the functions of DEGs, including KEGG, Metascape, PANTHER, and Cluego.

**Figure 4:**
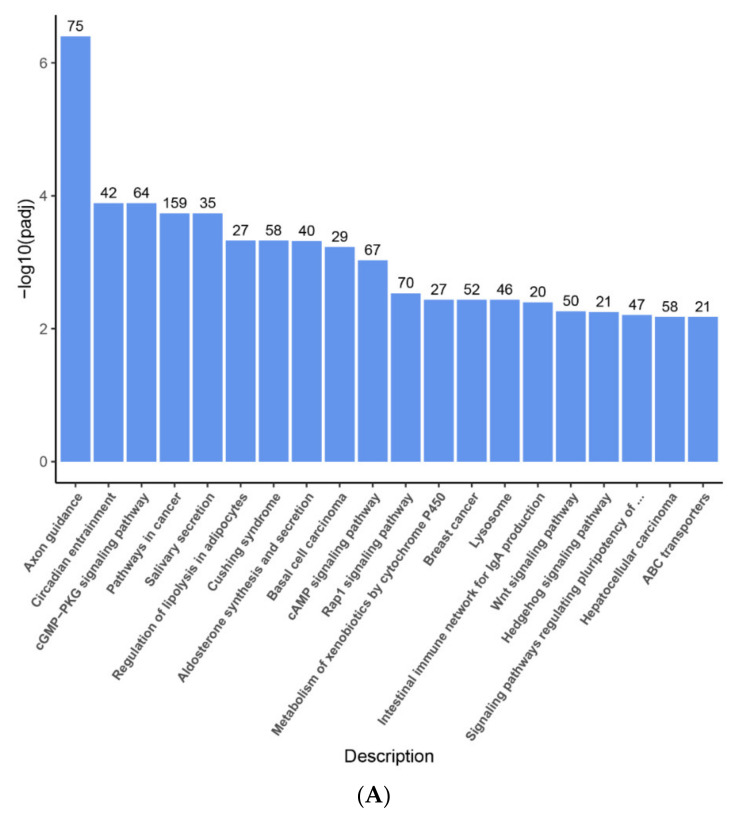

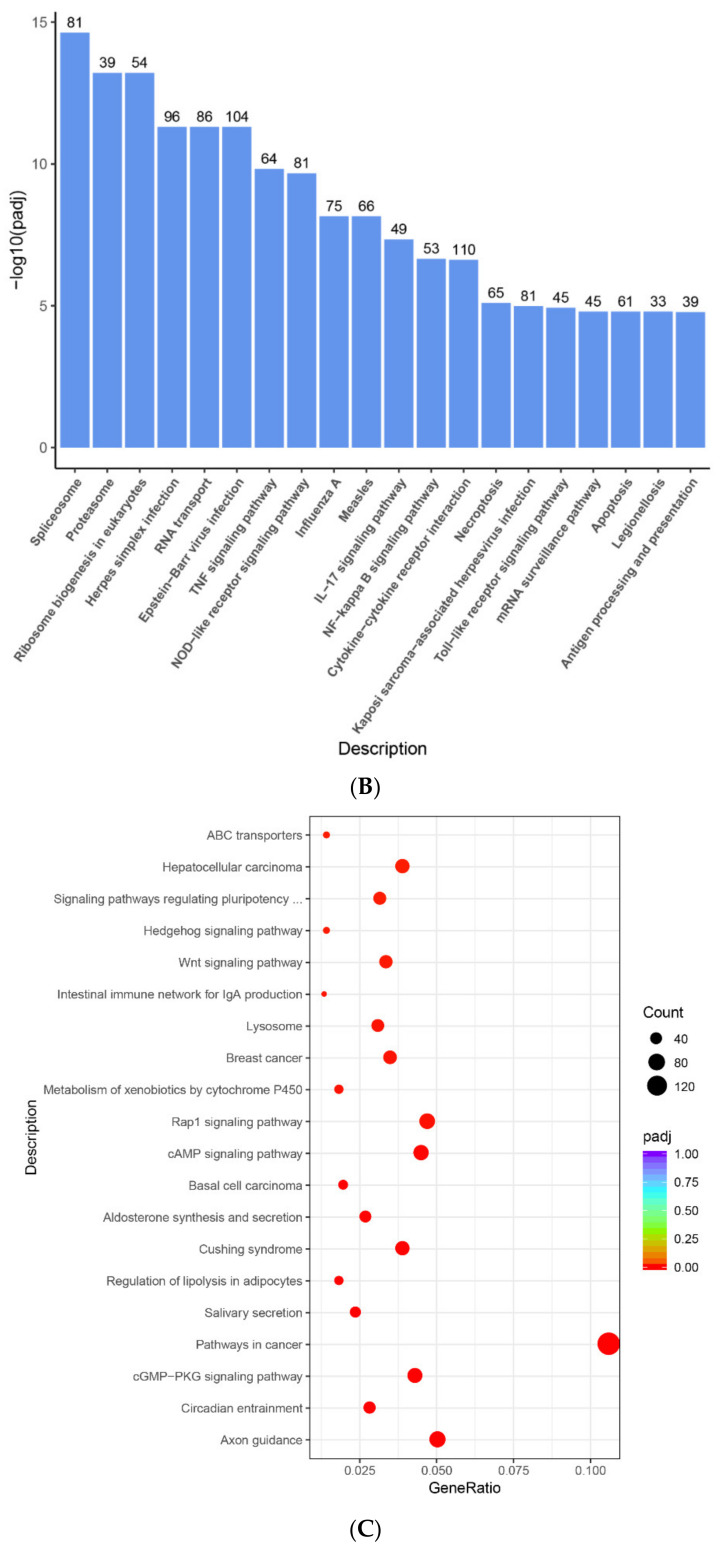

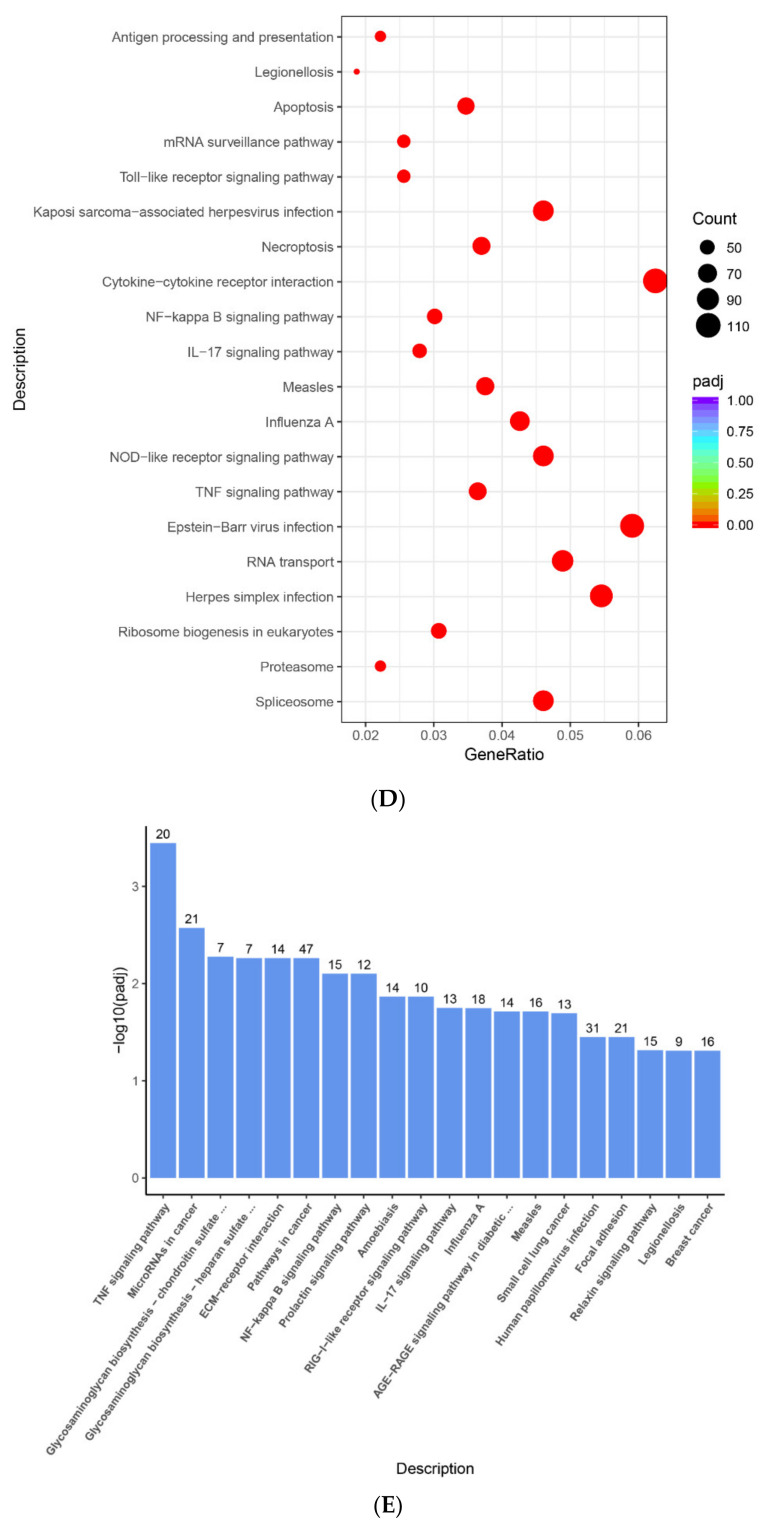

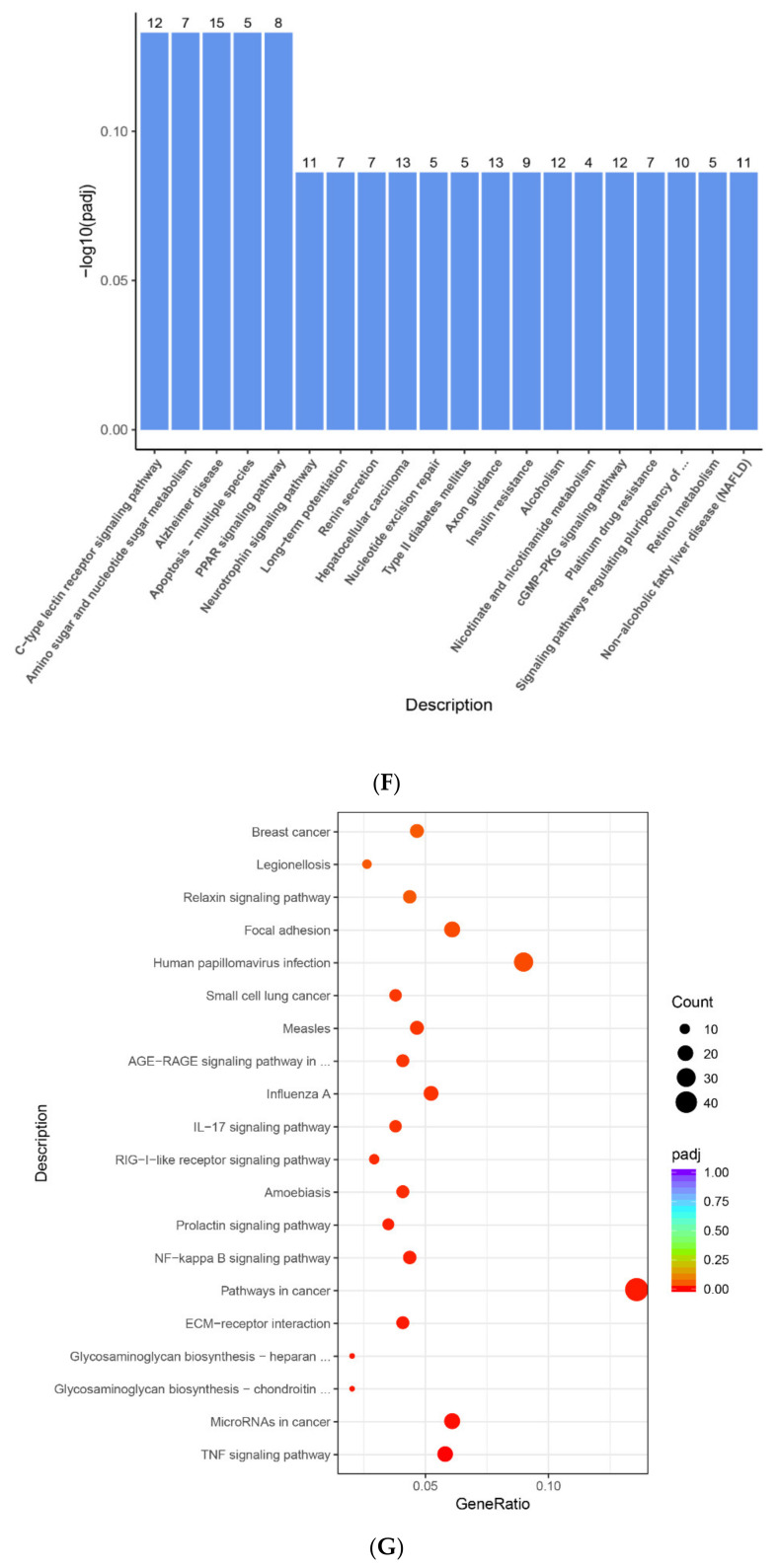

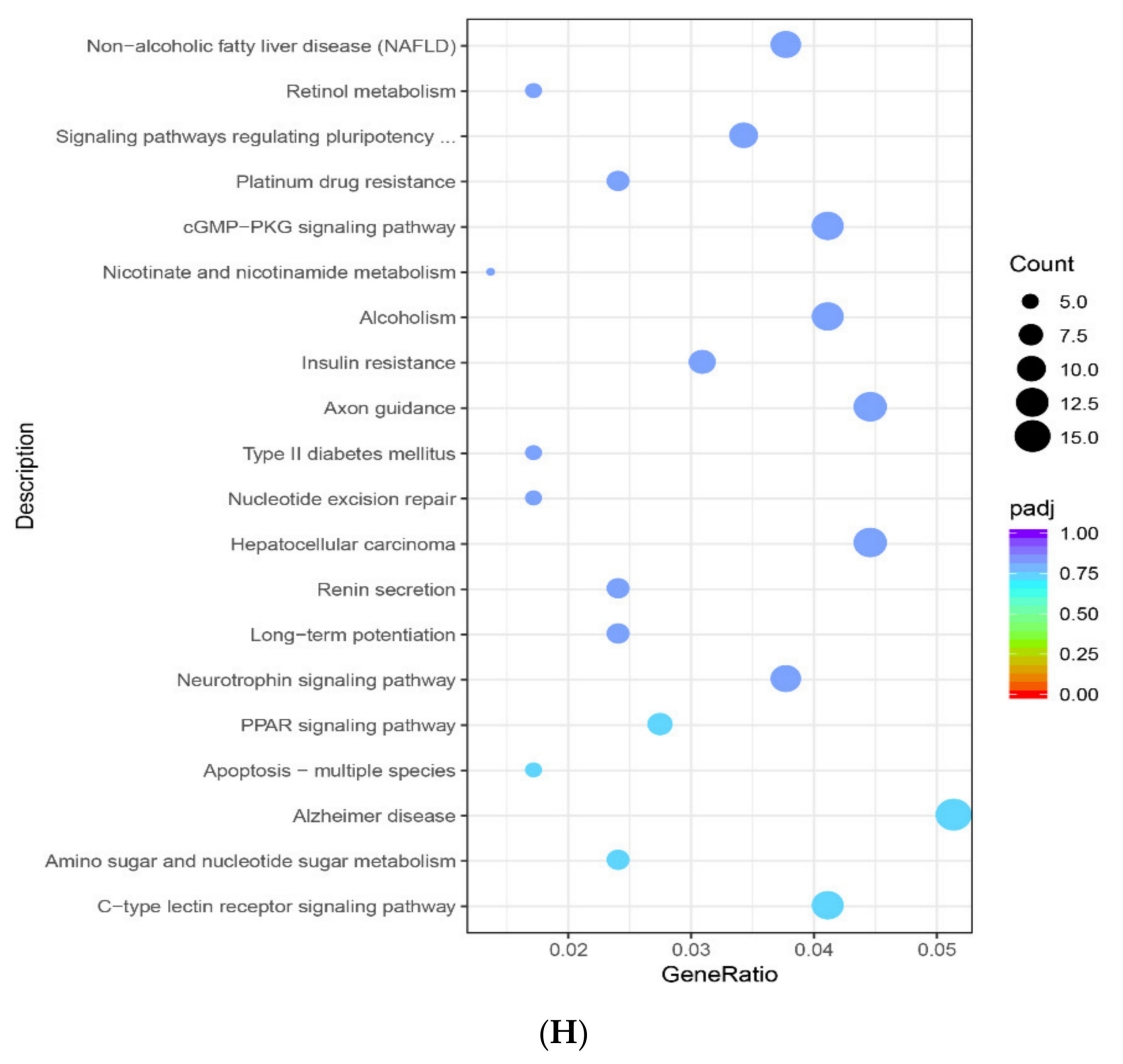
KEGG enrichment analysis of DEGs in the down-regulated DEGs (**A**,**C**) and up-regulated DEGs (**B**,**D**) of group 1 (LPS vs. Control) and group 2 (TAK vs. LPS) ((**E**,**G**) for down-regulated DEGs and (**F**,**H**) for up-regulated DEGs). The abscissa indicated the ratio of DEGs, which annotated the KEGG items to all DEGs.

The LPS-caused DEGs were submitted to the KEGG database for enrichment pathway analysis. The following biological pathways were inhibited by LPS: pathways in cancer (mmu05200; gene count: 159), human papillomavirus infection (mmu05165; gene count: 99), axon guidance (mmu04360; gene count: 75), ras signaling pathway (mmu04014; gene count: 73), rap1 signaling pathway (mmu04015; gene count: 70), cAMP signaling pathway (mmu04024; gene count: 67), and cGMP-PKG signaling pathway (mmu04022; gene count: 64) (Figure 4A,C and Supplementary Table S12). Meanwhile, LPS activated the following biological pathways: cytokine–cytokine receptor interaction (mmu04060; gene count: 110), Epstein–Barr virus infection (mmu 05169; gene count: 104), Herpes simplex infection (mmu05168); gene count:96), human T-cell leukemia virus 1 infection (mmu05166; gene count: 91), RNA transport (mmu03013; gene count: 86), human cytomegalovirus infection (mmu05163; gene count: 83), spliceosome (mmu03040; gene count: 81), and NOD-like receptor signaling pathway (mmu04621; gene count: 81) (Figure 4B,D and Supplementary Table S13).

TAK 242 inhibited a series of LPS-induced biological pathways: pathways in cancer (mmu05200; gene count: 47), human papillomavirus infection (mmu05165; gene count: 31), microRNAs in cancer (mmu05206; gene count: 21), focal adhesion (mmu04510; gene count: 21), and TNF signaling pathway (mmu04668; gene count: 20) (Supplementary Table S14) (Figure 4E,G). The following LPS-stimulated biological processes were activated by TAK 242: Alzheimer disease, renin secretion, nicotinate and nicotinamide metabolism, C-type lectin receptor signaling pathway, PPAR signaling pathway, and non-alcoholic fatty liver disease (NAFLD) (Figure 4F,H).

### 2.4. The Enrichment Pathway of DEGs-Metascape

**Figure 5:**
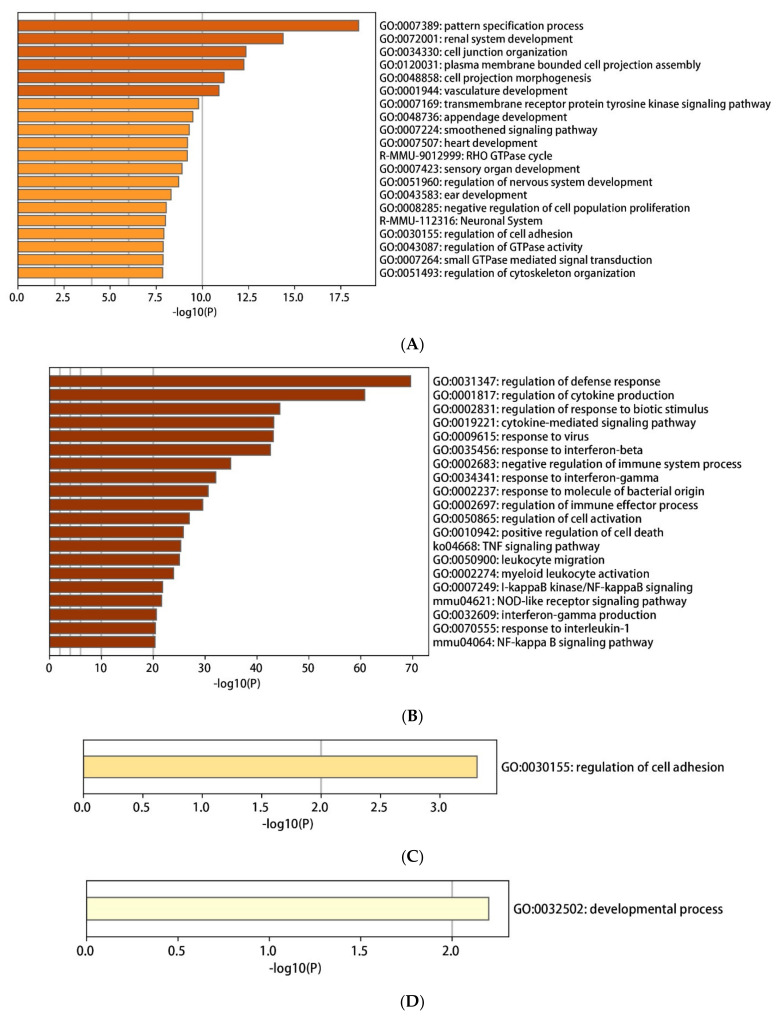

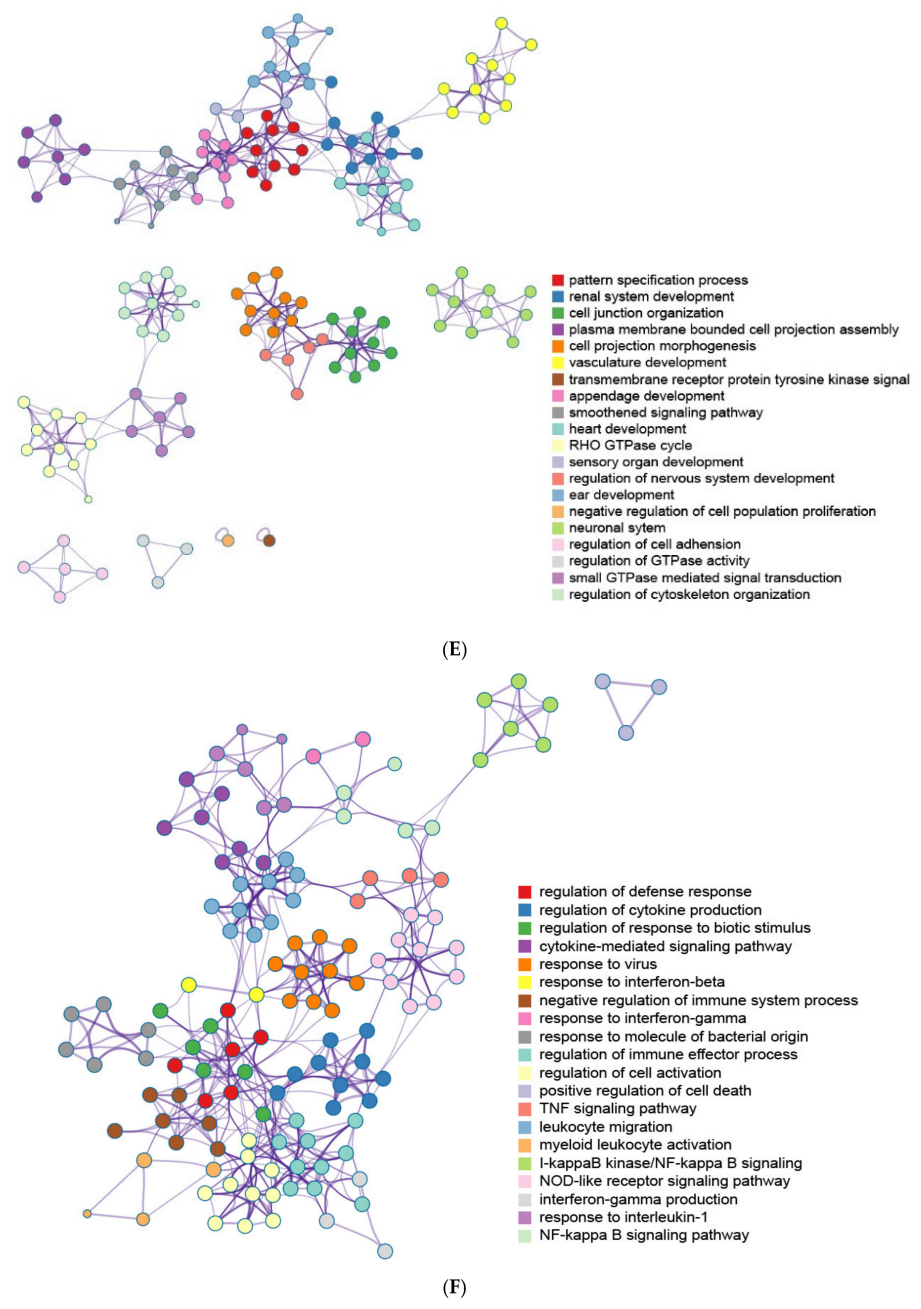

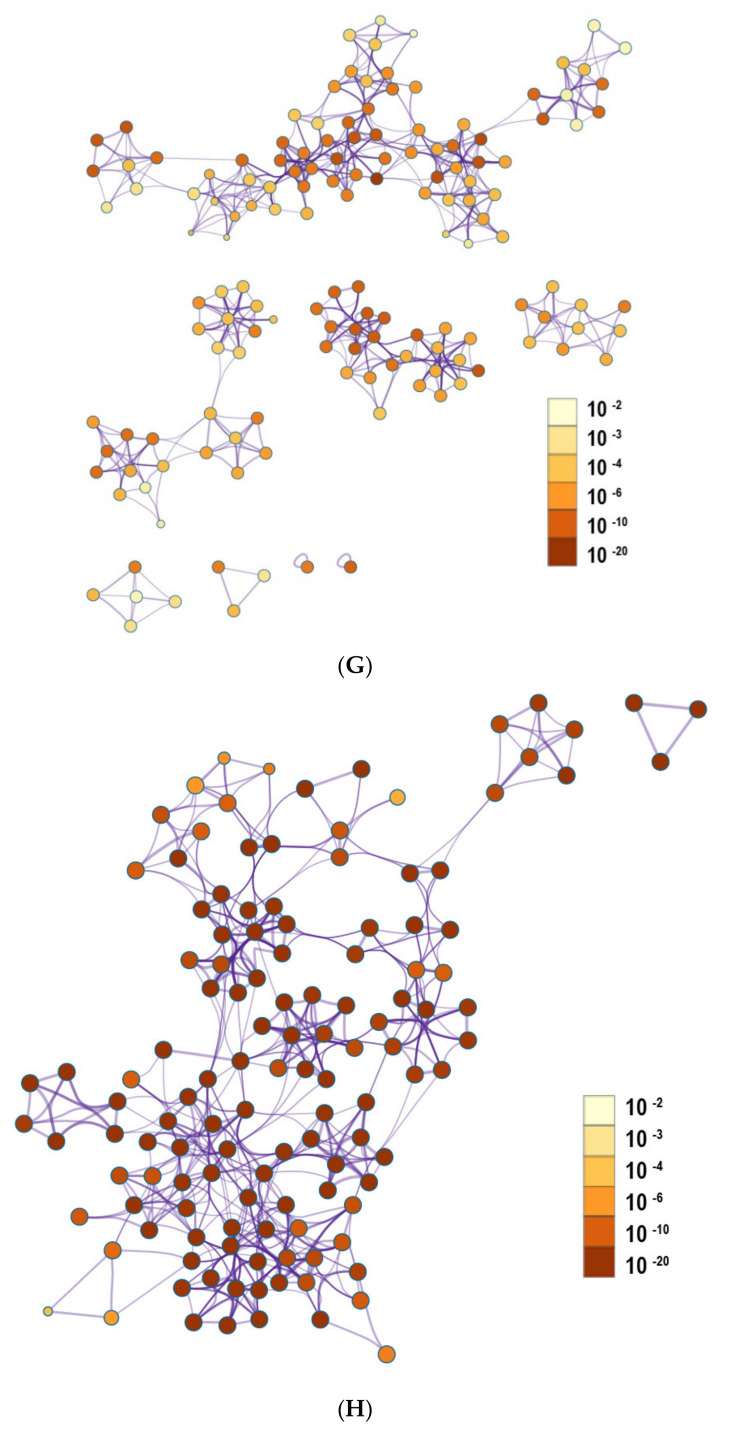
The enrichment analysis results of DEGs by using Metascape. (**A**–**D**) Bar plot of enriched biological pathways (colored by cluster ID) for down-regulated (**A**) and up-regulated DEGs (**B**) in group 1 (LPS vs. Control) and group 2 (TAK vs. LPS) (**E** and **G**, down-regulated DEGs; **F** and **H**, up-regulated DEGs). (**E**,**F**) The network plot of enriched biological pathways (colored by cluster) for down-regulated (**E**) and up-regulated DEGs (**F**) in group 1 (LPS vs. Control). (**G**,**H**) Network plot of enriched pathways (colored by *p*-value) for down-regulated (**G**) and up-regulated DEGs (**H**) in group 1 (LPS vs. Control).

By using an online tool Metascape, DEGs-mediated biology was enriched. The following biological pathways were inhibited by LPS: pattern specification process (GO:0007389), renal system development (GO:0072001), cell junction organization (GO:0034330), plasma membrane bounded cell projection assembly (GO:0120031), cell projection morphogenesis (GO:0048858), and vasculature development (GO:0001944) (Figure 5A).

Meanwhile, the biological pathways inhibited by LPS were as follows: regulation of defense response (GO:0031347), regulation of cytokine production (GO:0001817), regulation of response to biotic stimulus (GO:0002831), cytokine-mediated signaling pathway (GO:0019221), response to virus (GO:0009615), response to interferon-beta (GO:0035456), negative regulation of immune system process (GO:0002683), response to interferon-gamma (GO:0034341), response to molecule of bacterial origin (GO:0002237), regulation of immune effector process (GO:0002697), and regulation of cell activation (GO:0050865) (Figure 5B).

Only one LPS-inhibited biological pathway was inhibited by TAK 242: biological adhesion (GO:0022610) (Figure 5C). In contrast, TAK 242 activated an LPS-mediated biological process: blood vessel development (GO:0001568) (Figure 5D). The above-enriched pathways were aggregated and summarized into different network diagrams (Figure 5E–H).

### 2.5. Enrichment Pathway of DEGs-PANTHER

**Figure 6:**
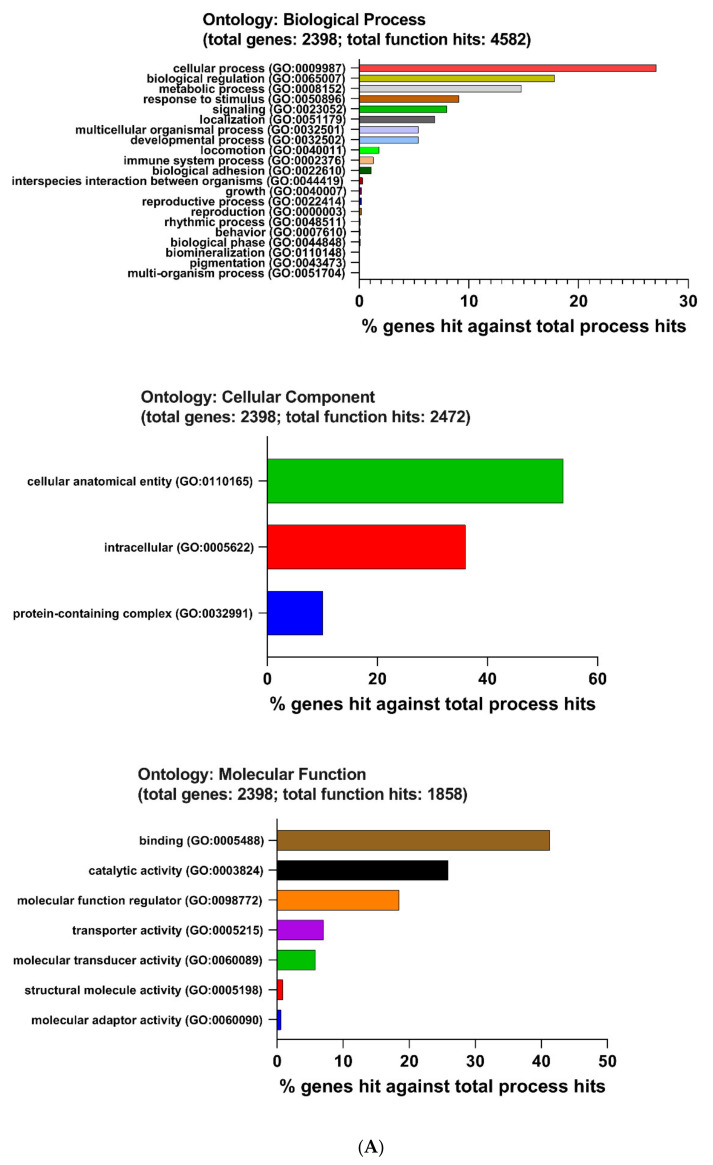

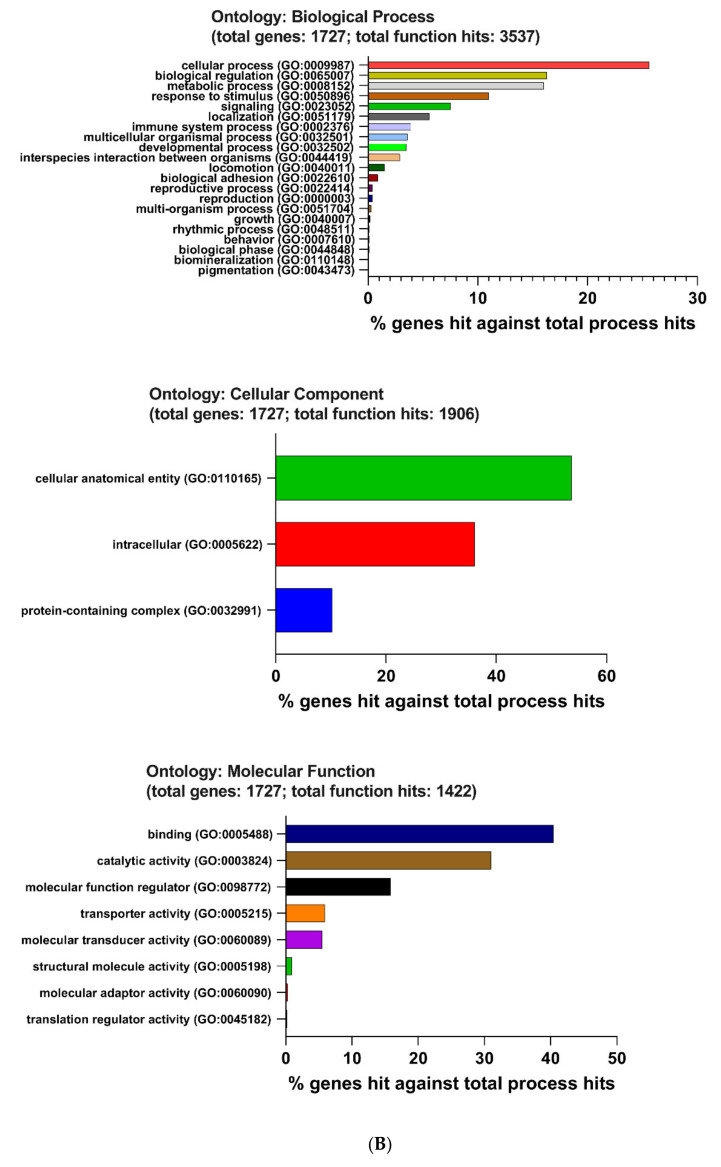

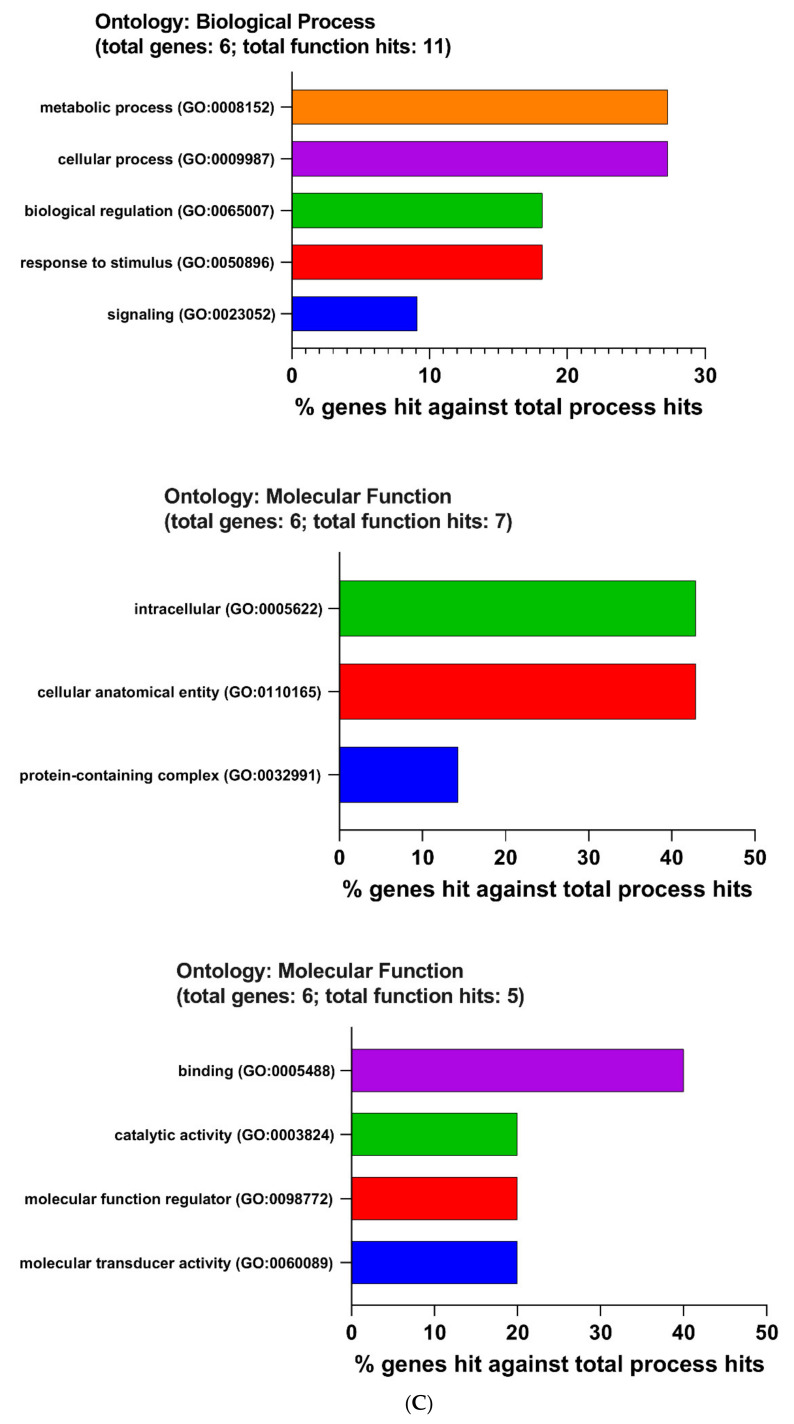

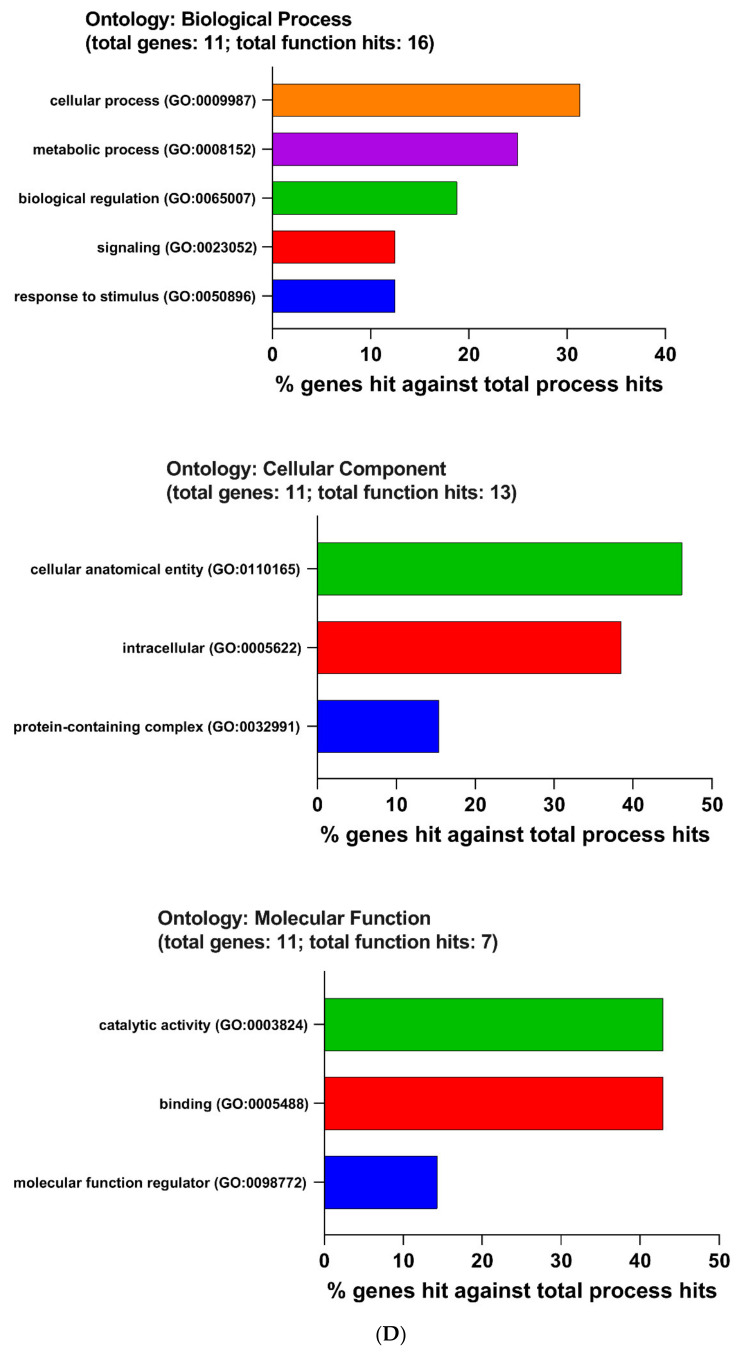
The enrichment analysis results of DEGs by using PANTHER. (**A**,**B**) Bar plot of enriched biological pathways for down-regulated (**A**) and up-regulated DEGs (**B**) in group 1 (LPS vs. Control). (**C**,**D**) Bar plot of enriched biological pathways for down-regulated (**C**) and up-regulated DEGs (**D**) in group 2 (TAK vs. LPS).

DEGs-mediated biology has been enriched by using the online tool PANTHER (Figure 6). The following biological pathways were inhibited by LPS. Biological process (BP): cellular process (GO:0009987, 27.10%), biological regulation (GO:0065007, 17.80%), metabolic process (GO:0008152, 14.80%), response to stimulus (GO:0050896, 9.10%), signaling (GO:0023052, 8.00%), localization (GO:0051179, 6.90%), developmental process (GO:0032502, 5.40%), multicellular organismal process (GO:0032501, 5.40%), locomotion (GO:0040011, 1.80%), immune system process (GO:0002376, 1.30%), and biological adhesion (GO:0022610, 1.10%). Molecular function (MF): binding (GO:0005488, 41.30%), catalytic activity (GO:0003824, 25.90%), molecular function regulator (GO:0098772, 18.50%), transporter activity (GO:0005215, 7.00%), molecular transducer activity (GO:0060089, 5.80%), structural molecule activity (GO:0005198, 0.90%), and molecular adaptor activity (GO:0060090, 0.60%). Cellular compartment (CC): cellular anatomical entity (GO:0110165, 53.80%), intracellular (GO:0005622, 36.00%), and protein-containing complex (GO:0032991, 10.10%).

LPS activated the following biological pathways. Biological process (BP): cellular process (GO:0009987, 25.60%), biological regulation (GO:0065007, 16.30%), metabolic process (GO:0008152, 16.00%), response to stimulus (GO:0050896, 11.00%), signaling (GO:0023052, 7.50%), localization (GO:0051179, 5.60%), immune system process (GO:0002376, 3.90%), multicellular organismal process (GO:0032501, 3.60%), developmental process (GO:0032502, 3.50%), interspecies interaction between organisms (GO:0044419, 2.90%), and locomotion (GO:0040011, 1.50%). Molecular function (MF): binding (GO:0005488, 40.40%), catalytic activity (GO:0003824, 31.00%), molecular function regulator (GO:0098772, 15.80%), transporter activity (GO:0005215, 5.90%), molecular transducer activity (GO:0060089, 5.50%), structural molecule activity (GO:0005198, 0.90%), molecular adaptor activity (GO:0060090, 0.30%), and translation regulator activity (GO:0045182, 0.20%). Cellular compartment (CC): cellular anatomical entity (GO:0110165, 53.70%), intracellular (GO:0005622, 36.10%), and protein-containing complex (GO:0032991, 10.20%).

These following pathways were suppressed by the TAK 242. Biological process (BP): cellular process (GO:0009987, 27.30%, metabolic process (GO:0008152, 27.30%), response to stimulus (GO:0050896, 18.20%), biological regulation (GO:0065007, 18.20%), signaling (GO:0023052, 9.10%). Molecular function (MF): binding (GO:0005488, 40.00%), molecular transducer activity (GO:0060089, 20.00%), molecular function regulator (GO:0098772, 20.00%), and catalytic activity (GO:0003824, 20.00%). Cellular compartment (CC): cellular anatomical entity (GO:0110165, 42.90%), intracellular (GO:0005622, 42.90%), and protein-containing complex (GO:0032991, 14.30%).

TAK 242 stimulated the following LPS-induced biological pathways. Biological process (BP): cellular process (GO:0009987, 31.30%), metabolic process (GO:0008152, 25.00%), biological regulation (GO:0065007, 18.80%), response to stimulus (GO:0050896, 12.50%), and signaling (GO:0023052, 12.50%). Molecular function (MF): binding (GO:0005488, 42.90%), catalytic activity (GO:0003824, 42.90%), and molecular function regulator (GO:0098772, 14.30%). Cellular compartment (CC): cellular anatomical entity (GO:0110165, 46.20%), intracellular (GO:0005622, 38.50%), and protein-containing complex (GO:0032991, 15.40%).

### 2.6. The Enrichment Pathway of DEGs-Cluego

By using the tool Cluego, DEGs-mediated biological pathways were enriched.

**Figure 7:**
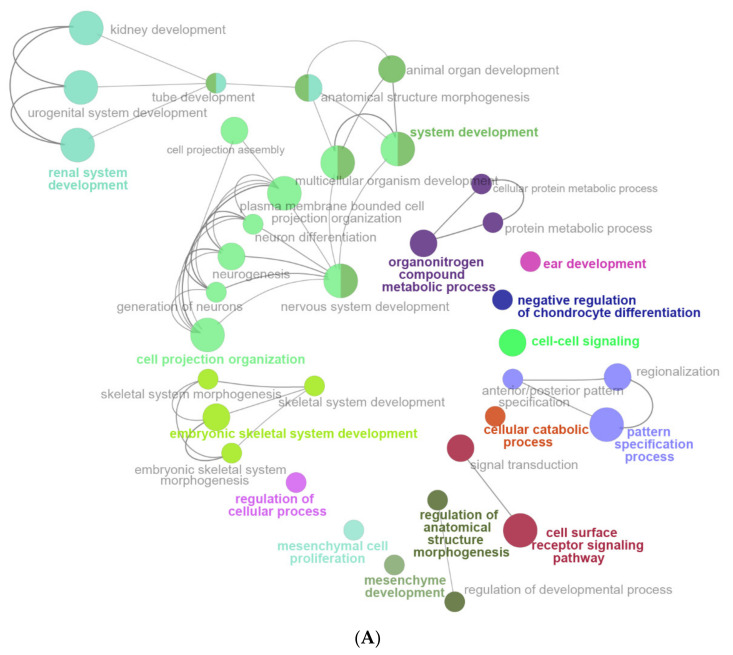

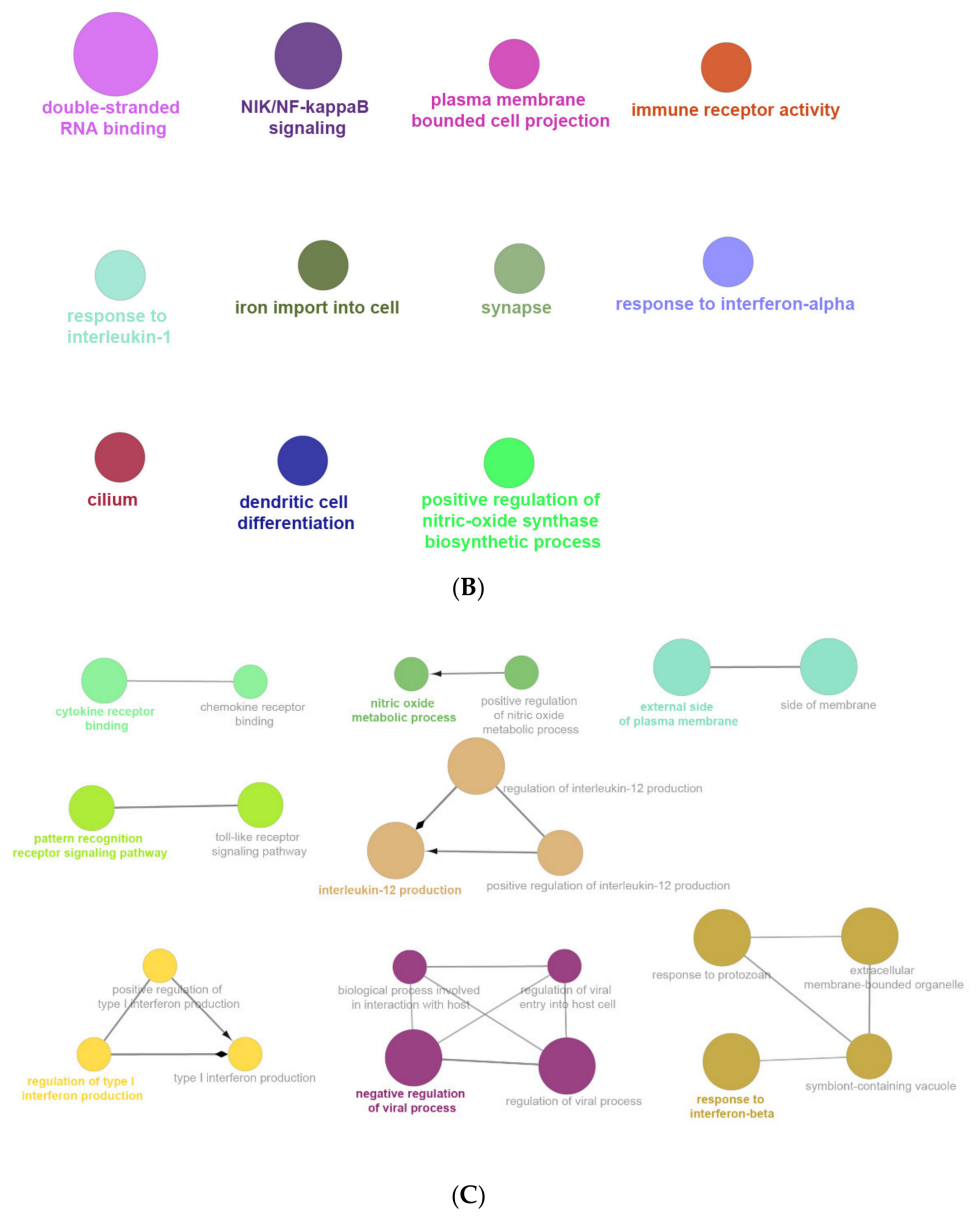

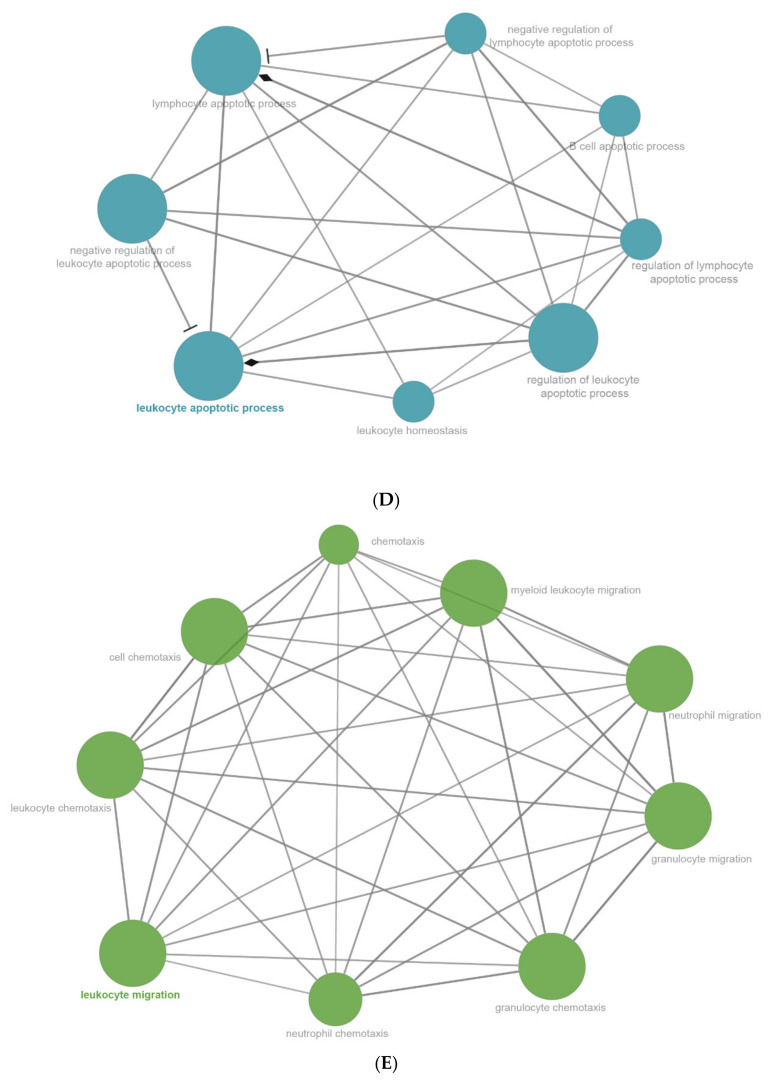

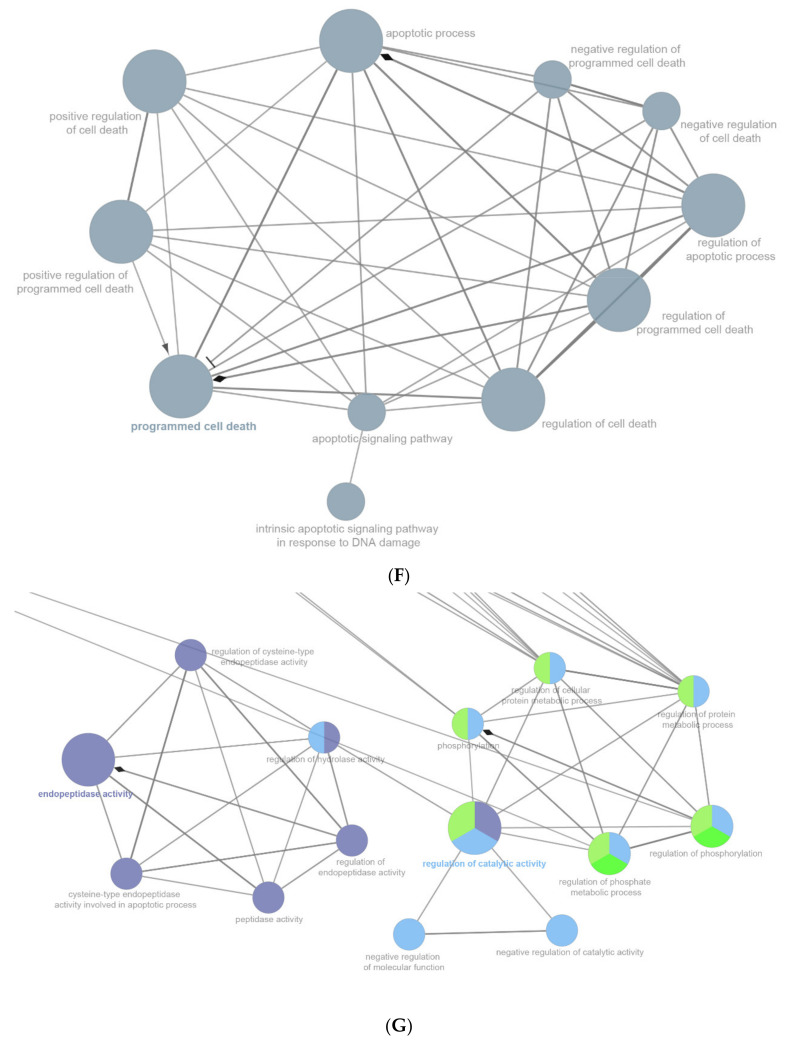

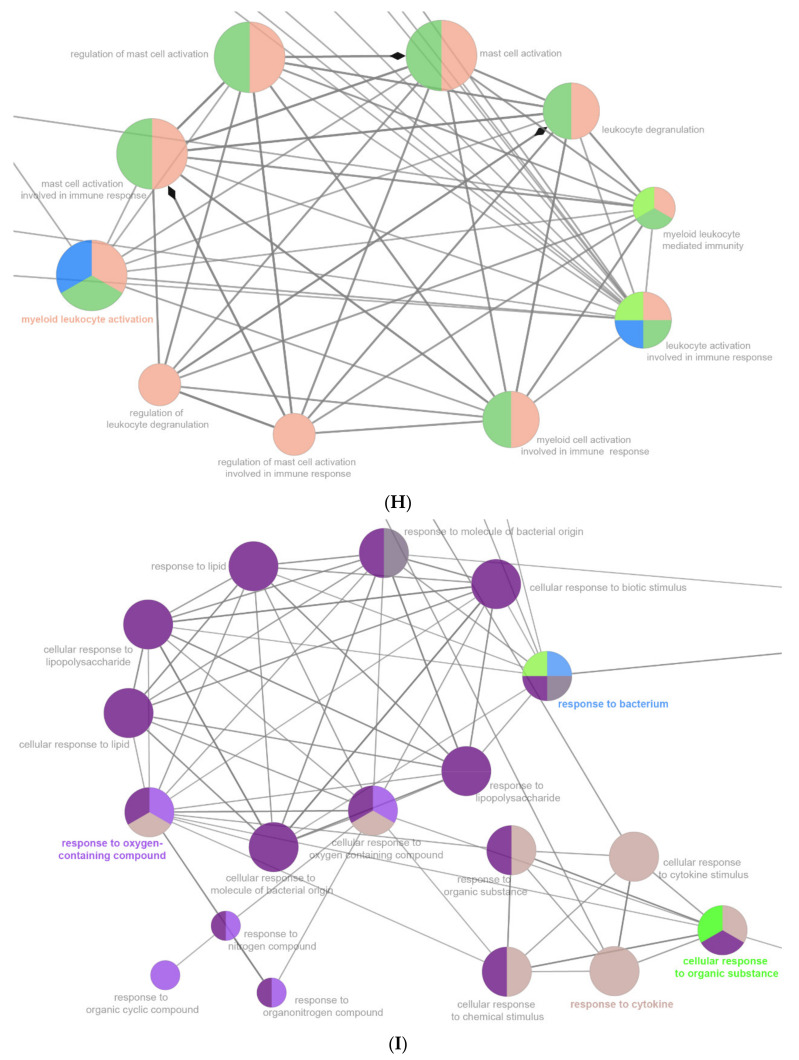

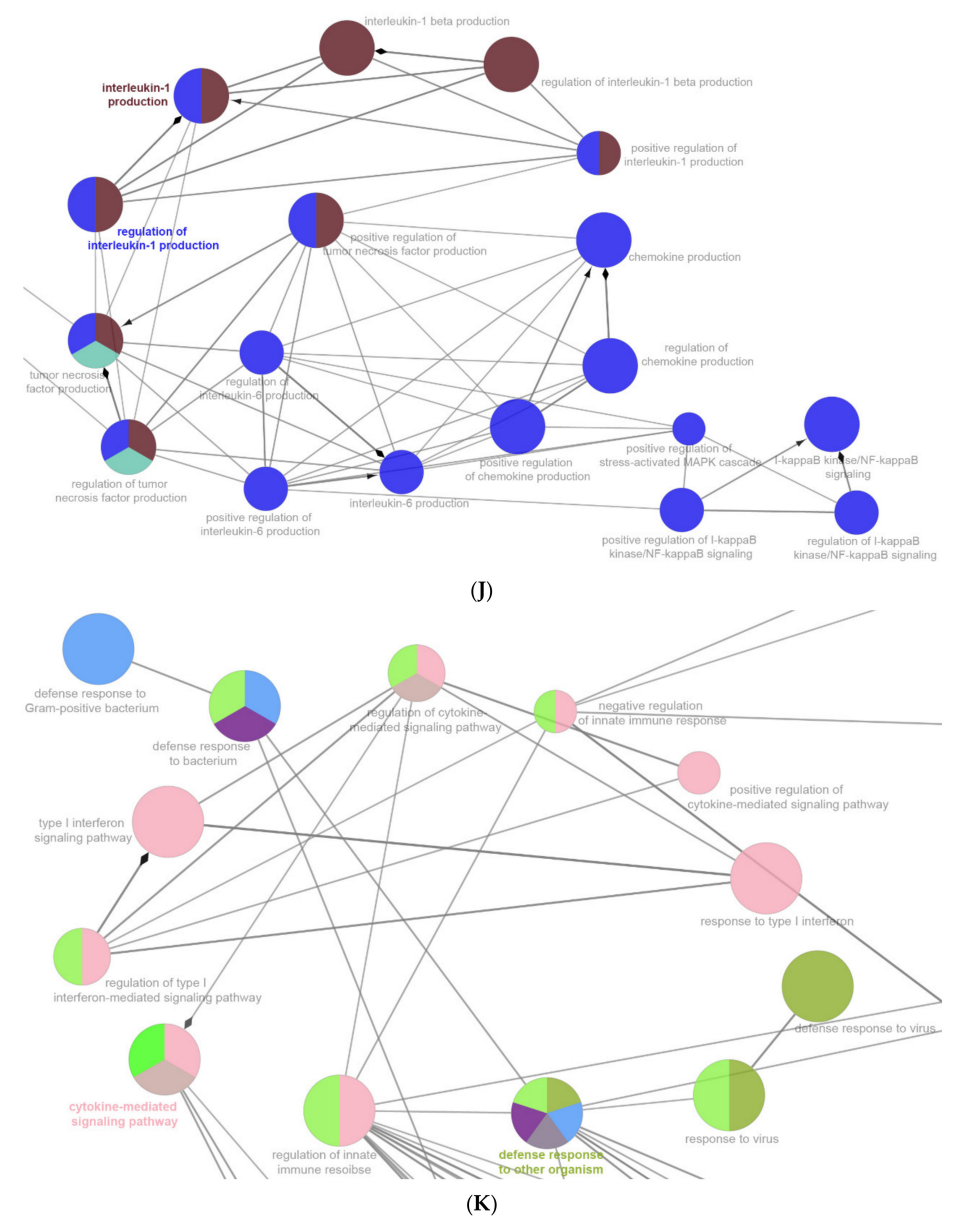

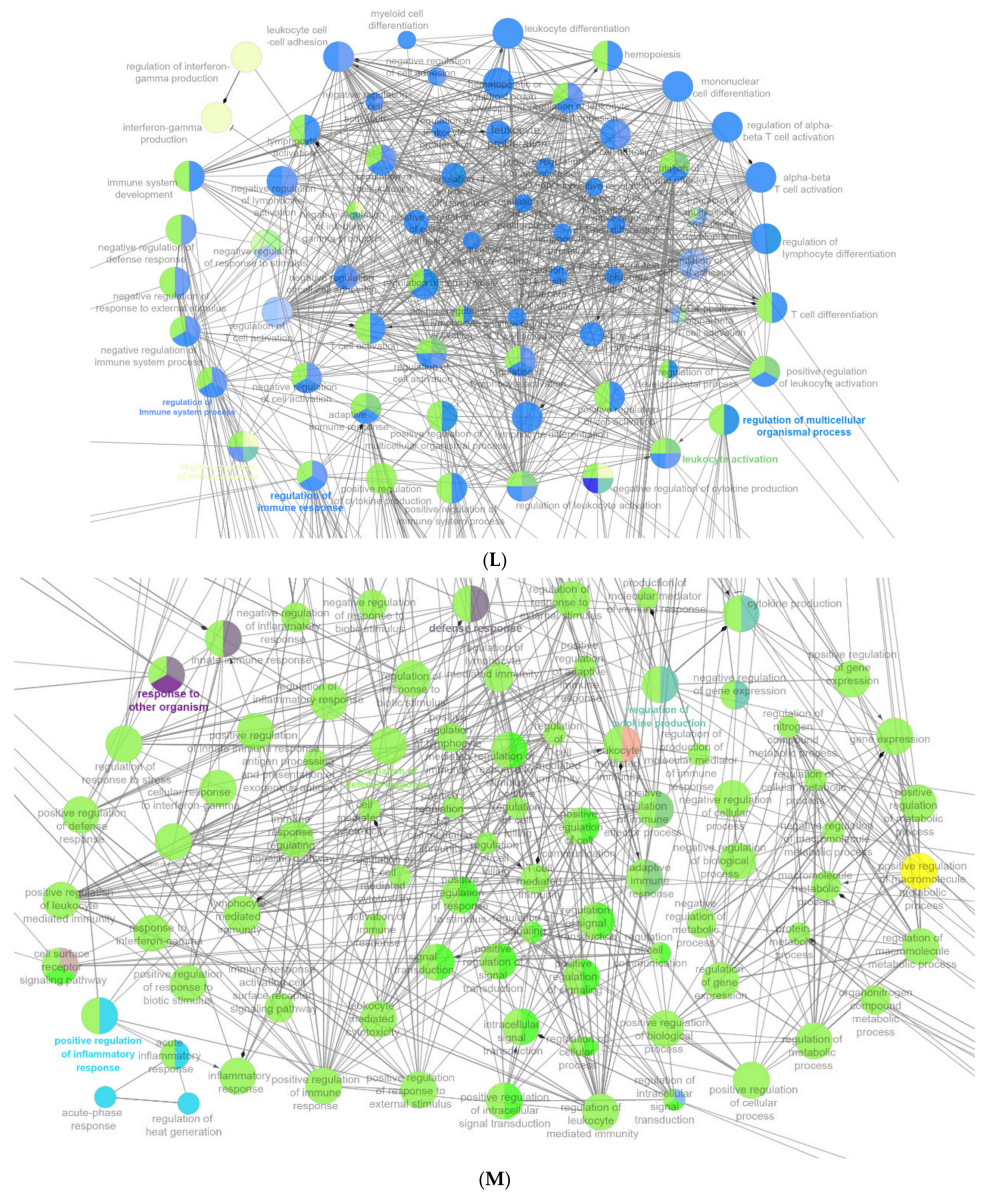
The enrichment analysis results of DEGs by using ClueGO. (**A**) Enriched biological pathways of down-regulated DEGs in group 1 (LPS vs. Control), including system development, organonitrogen compound metabolic process, negative regulation of chondrocyte differentiation, cell–cell signaling, cellular catabolic process, pattern specification process, cell surface receptor signaling pathway, regulation of cellular process, mesenchymal cell proliferation, and mesenchymal development. (**B**–**M**) Enriched biological pathways of up-regulated DEGs in group 2 (TAK vs. LPS). (**B**) Double-stranded RNA binding, NIK/NF-kappaB signaling plasma membrane-bounded cell projection, plasma membrane-bounded cell projection, immune receptor activity, response to interleukin-1, iron import into cell, synapse, response to interferon-alpha, cilium, dendritic cell differentiation, and positive regulation of nitric-oxide synthase biosynthetic process. (**C**) Cytokine receptor binding, nitric oxide metabolic process, external side of plasma membrane, pattern recognition receptor signaling pathway, interleukin-12 production, regulation of type 1 interferon production, negative regulation of viral process, and response to interferon-beta. (**D**) Leukocyte apoptotic process. (**E**) Leukocyte migration. (**F**) Programmed cell death. (**G**) Endopeptidase activity. (**H**) regulation of catalytic activity. (**H**) Myeloid leukocyte activation. (**I**) Response to bacterium, response to oxygen-containing compound, and cellular response to organic substance. (**J**) Interleukin-1 production and regulation of interleukin-1 production. (**K**) Cytokine-mediated signaling pathway and defense response to the organism. (**L**) Regulation of multicellular organismal process, regulation of immune response, regulation of immune system process, leukocyte activation, and negative regulation of immune response. (**M**) Positive regulation of inflammatory response, response to other organisms, response to other organisms, and regulation of cytokine production.

The following biological pathways were inhibited by LPS: system development, organonitrogen compound metabolic process, negative regulation of chondrocyte differentiation, cell–cell signaling, cellular catabolic process, pattern specification process, cell surface receptor signaling pathway, regulation of cellular process, mesenchymal cell proliferation, and mesenchymal development (Figure 7A).

LPS activated the following biological pathways: double-stranded RNA binding, NIK/NF-kappaB signaling plasma membrane-bounded cell projection, plasma membrane-bounded cell projection, immune receptor activity, response to interleukin-1, iron import into cell, synapse, response to interferon-alpha, cilium, dendritic cell differentiation, and positive regulation of nitric-oxide synthase biosynthetic process (Figure 7B). Cytokine receptor binding, nitric oxide metabolic process, external side of plasma membrane, pattern recognition receptor signaling pathway, interleukin-12 production, regulation of type 1 interferon production, negative regulation of viral process, and response to interferon-beta (Figure 7C). Leukocyte apoptotic process (Figure 7D). Leukocyte migration (Figure 7E). Programmed cell death (Figure 7F). Endopeptidase activity (Figure 7G). regulation of catalytic activity (Figure 7H). Myeloid leukocyte activation (Figure 7H). Response to bacterium, response to oxygen-containing compound, and cellular response to organic substance (Figure 7I). Interleukin-1 production and regulation of interleukin-1 production (Figure 7J). Cytokine-mediated signaling pathway and defense response to the organism (Figure 7K). Regulation of multicellular organismal process, regulation of immune response, regulation of immune system process, leukocyte activation, and negative regulation of immune response (Figure 7L). Positive regulation of inflammatory response, response to other organisms, response to other organisms, and regulation of cytokine production (Figure 7M).

**Figure 8:**
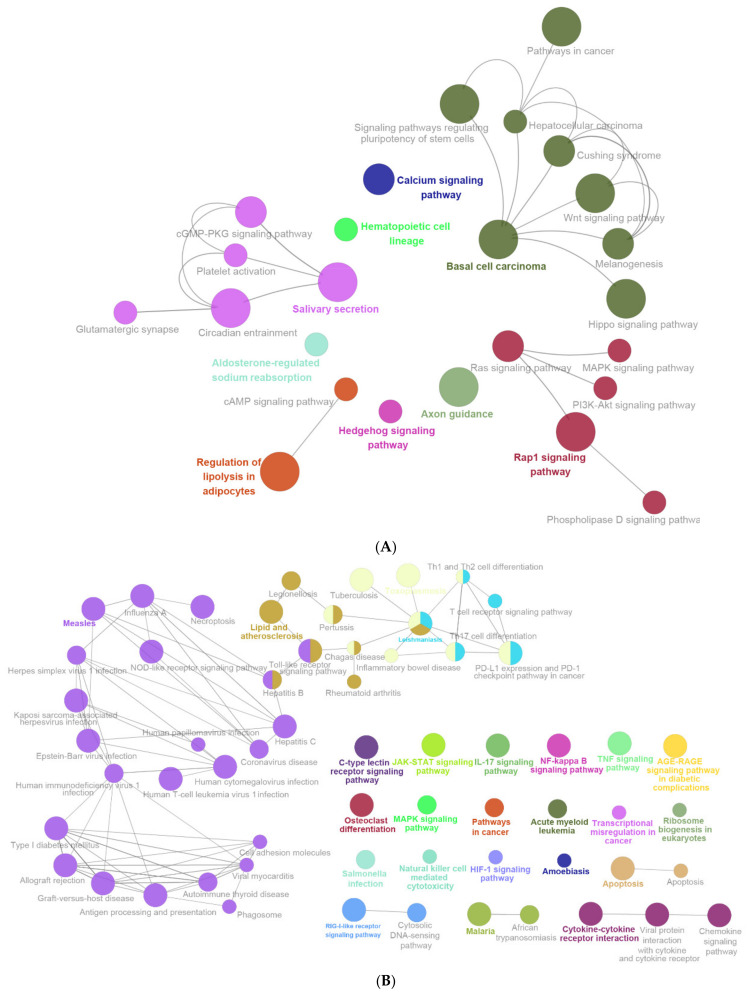
The enrichment analysis results of DEGs by using ClueGO (KEGG database). (**A**) Enriched biological pathways of down-regulated DEGs in group 1 (LPS vs. Control), including calcium signaling pathway, hematopoietic cell lineage, basal cell carcinoma, axon guidance, rap1 signaling, hedgehog signaling pathway, and regulation of lipolysis in adipocytes. (**B**) Enriched biological pathways of up-regulated DEGs in group 1 (LPS vs. Control), including C-type lectin receptor signaling pathway, JAK-STAT signaling pathway, IL-17 signaling pathway, NF-kappaB signaling pathway, TNF signaling pathway, AGE-RAGE signaling pathway in diabetic complications, osteoclast differentiation, MAPK signaling pathway, pathways in cancer, acute myeloid leukemia, transcriptional misregulation in cancer, ribosome biogenesis in eukaryotes, salmonella infection natural killer cell-mediated cytotoxicity, HIF-1 signaling pathway, amoebiasis, apoptosis, RIG-1-like receptor signaling pathway, malaria, and cytokine–cytokine receptor interaction.

The following biological pathways were suppressed by LPS (KEGG database): calcium signaling pathway, hematopoietic cell lineage, basal cell carcinoma, axon guidance, rap1 signaling, hedgehog signaling pathway, and regulation of lipolysis in adipocytes (Figure 8A).

LPS stimulated the following biological pathways (KEGG database): C-type lectin receptor signaling pathway, JAK-STAT signaling pathway, IL-17 signaling pathway, NF-kappaB signaling pathway, TNF signaling pathway, AGE-RAGE signaling pathway in diabetic complications, osteoclast differentiation, MAPK signaling pathway, pathways in cancer, acute myeloid leukemia, transcriptional misregulation in cancer, ribosome biogenesis in eukaryotes, salmonella infection natural killer cell-mediated cytotoxicity HIF-1 signaling pathway, amoebiasis, apoptosis, RIG-1-like receptor signaling pathway, malaria, and cytokine-cytokine receptor interaction (Figure 8A). Besides, the following pathways were enriched by the up-regulated DEGs in group 1 (LPS vs. Control): C-type lectin receptor signaling pathway, JAK-STAT signaling pathway, IL-17 signaling pathway, NF-kappaB signaling pathway, TNF signaling pathway, AGE-RAGE signaling pathway in diabetic complications, osteoclast differentiation, MAPK signaling pathway, pathways in cancer, acute myeloid leukemia, transcriptional misregulation in cancer, ribosome biogenesis in eukaryotes, salmonella infection natural killer cell-mediated cytotoxicity, HIF-1 signaling pathway, amoebiasis, apoptosis, RIG-1-like receptor signaling pathway, malaria, and cytokine-cytokine receptor interaction (Figure 8B).

### 2.7. Transcriptional Factors Tied to DEGs

With the help of the online tool TRRUST (Version 2) (https://www.grnpedia.org/trrust/, accessed on 29 August 2021), the transcription factor pathway represented by DEGs was analyzed. LPS inhibited a series of transcription factor pathways in mouse peritoneal tissue, including *Rbpj, Isl1, Tbx1, Nkx2-5, Gata2, Snai1, Mybl2, Nfatc1, Gata6*, and *Mecp2* (Table S15). In contrast, some transcription factors were activated by LPS, such as *Nfkb1, Stat1, Rela, Jun, Irf1, Rel, Sp1, Ikbkb, Trp53*, and *Irf8* (Supplementary Table S16). In addition, TAK 242 causes too few DEGs to obtain transcription factors.

### 2.8. Protein-Protein Interaction (PPI) Network of DEGs

**Figure 9:**
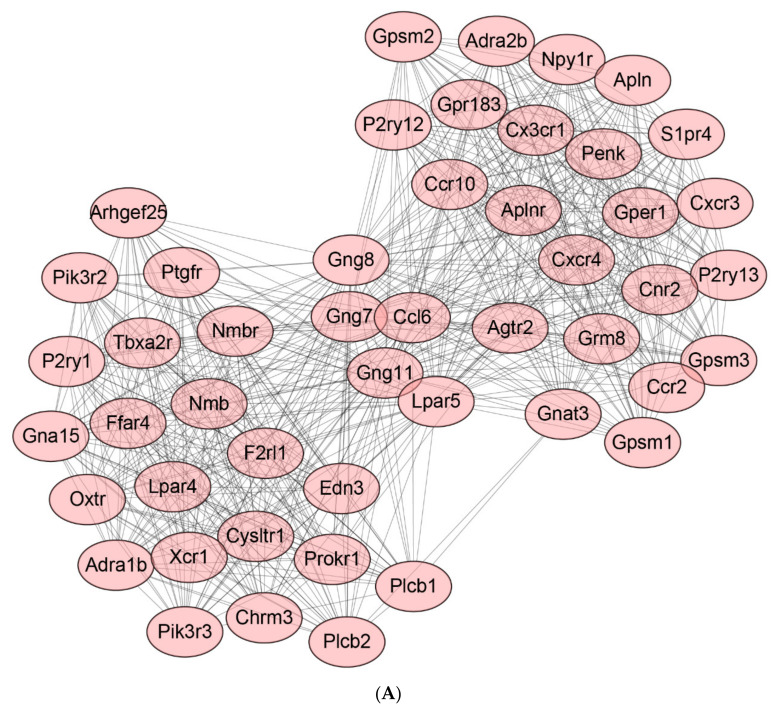

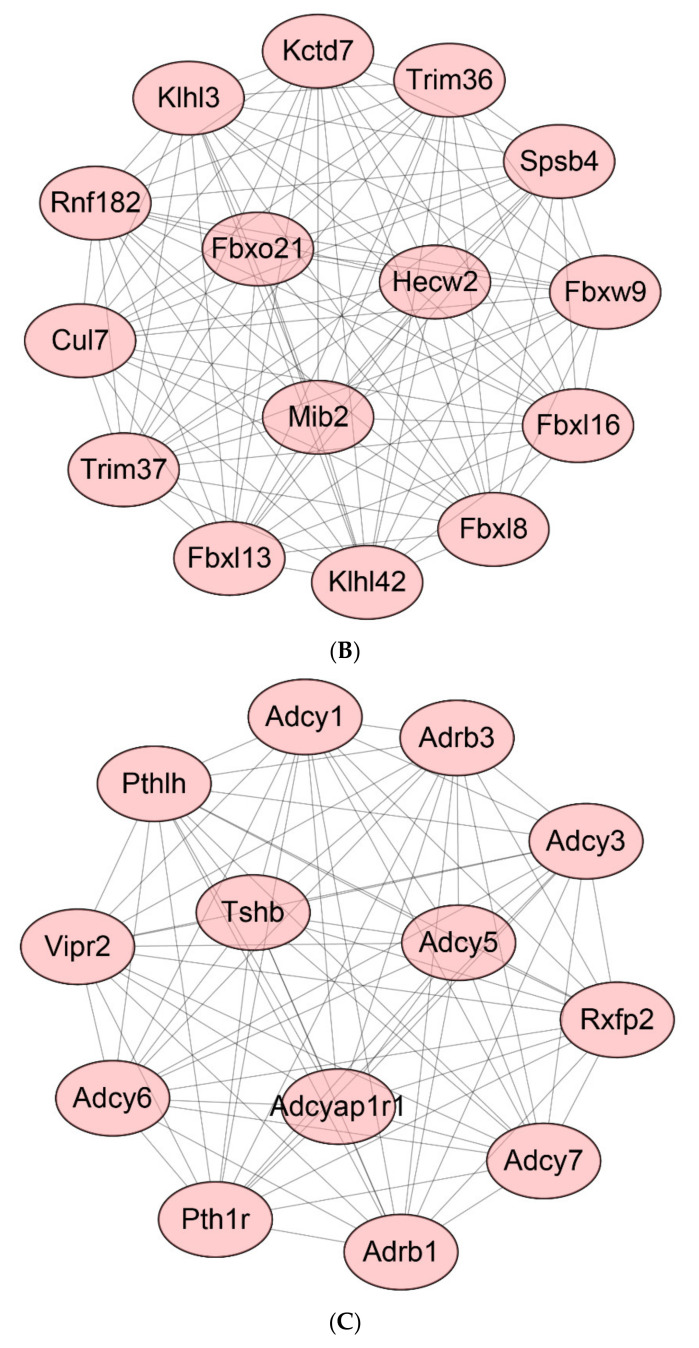

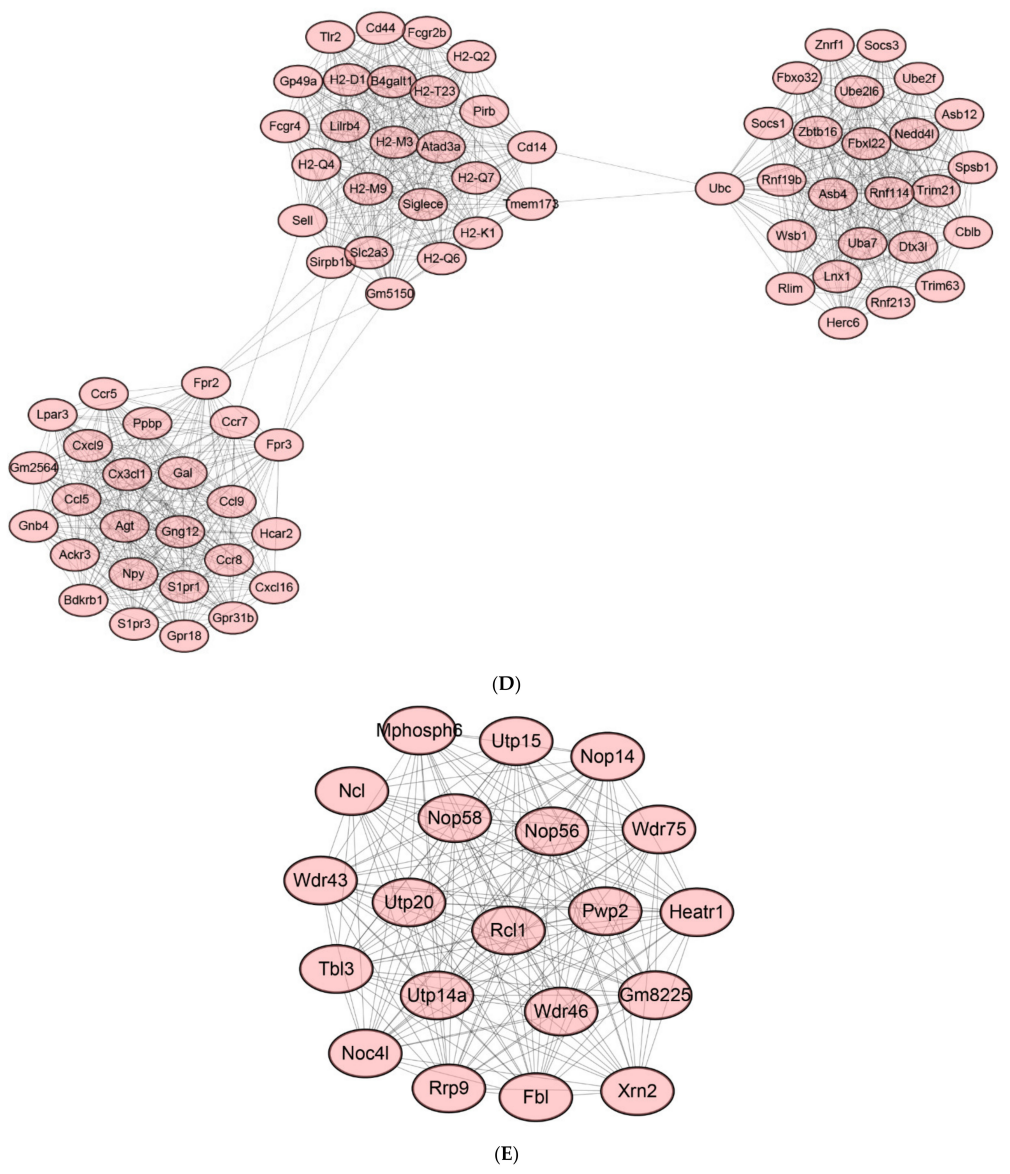

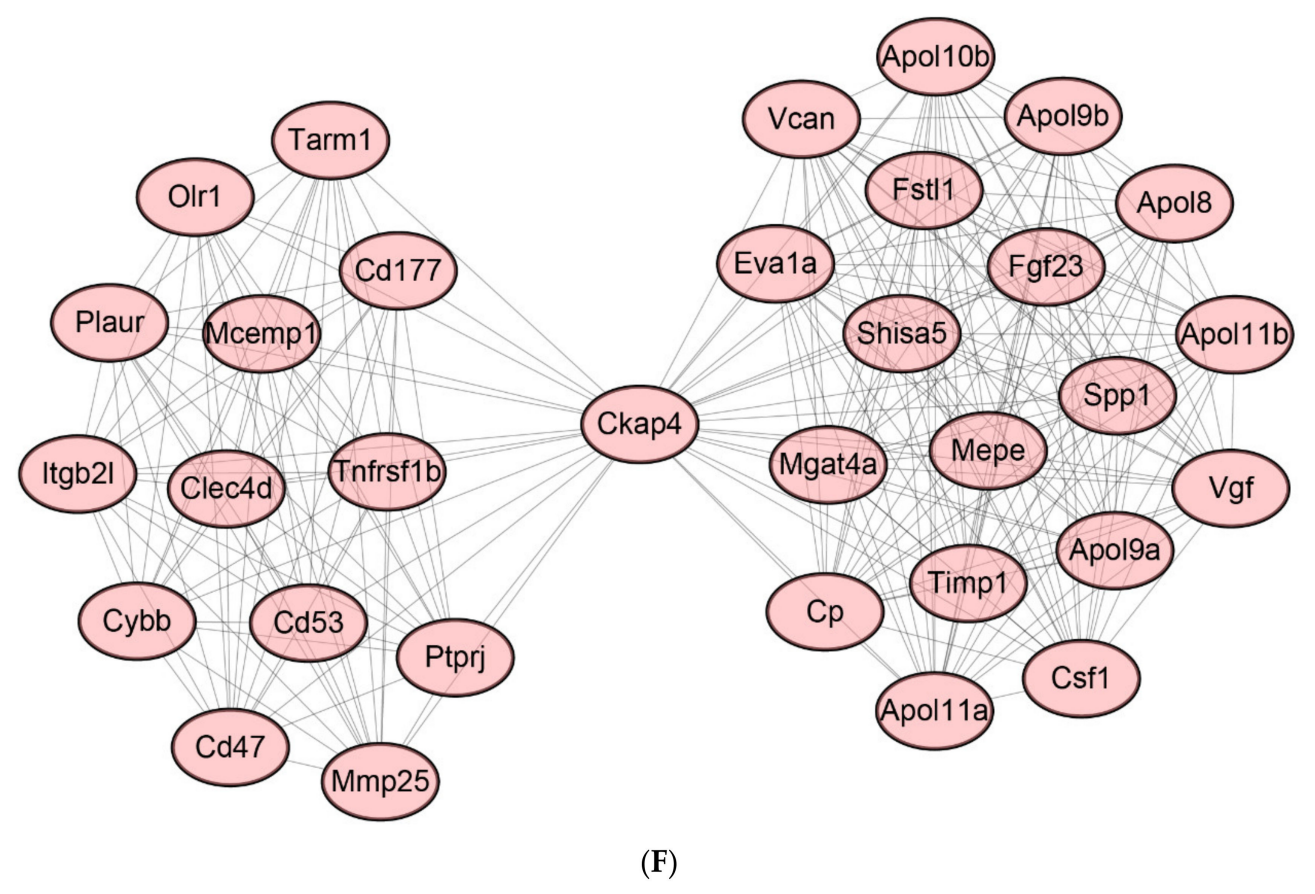
The protein–protein interaction analysis of DEGs by using the MCODE plugin of Cytoscape. (**A**–**C**) Protein–protein interaction networks of down-regulated DEGs in group 1 (LPS vs. Control). (**D**–**F**) Protein–protein interaction networks of up-regulated DEGs in group 1 (LPS vs. Control).

The protein–protein interaction (PPI) network was used to analyze the LPS-induced protein interaction network in the mouse peritoneum (Figure 9). Based on the DEGs suppressed by LPS, 3 PPI networks were obtained, with scores of 28.043, 15, and 13, respectively. At the same time, LPS inhibited the three DEGs gene networks with scores of 24.568, 19.684, and 16.903, respectively.

### 2.9. Identification of Hub Genes

A total of 4201 genes were changed by LPS (2442 down-regulated DEGs and 1759 up-regulated DEGs) (Figure 3). Interestingly, TAK 242 treatment only changed the expression of a few DEGs, which suggested that the proteins encoded by these genes may interact with TAK 242. Therefore, DEGs affected by TAK 242 were listed, including some down-regulated DEGs (*Sele, 9930111J21Rik2, Gzmb*, and *Lpar3*) and up-regulated DEGs (*Card10, Plekhh3, Higd1b, Gm12689, Plxdc1*, and *Pkn3*). All hub genes were protein-coding genes, and their functions were systematically investigated (Supplementary Tables S17 and S18).

### 2.10. qPCR Detection of Hub Genes

**Figure 10:**
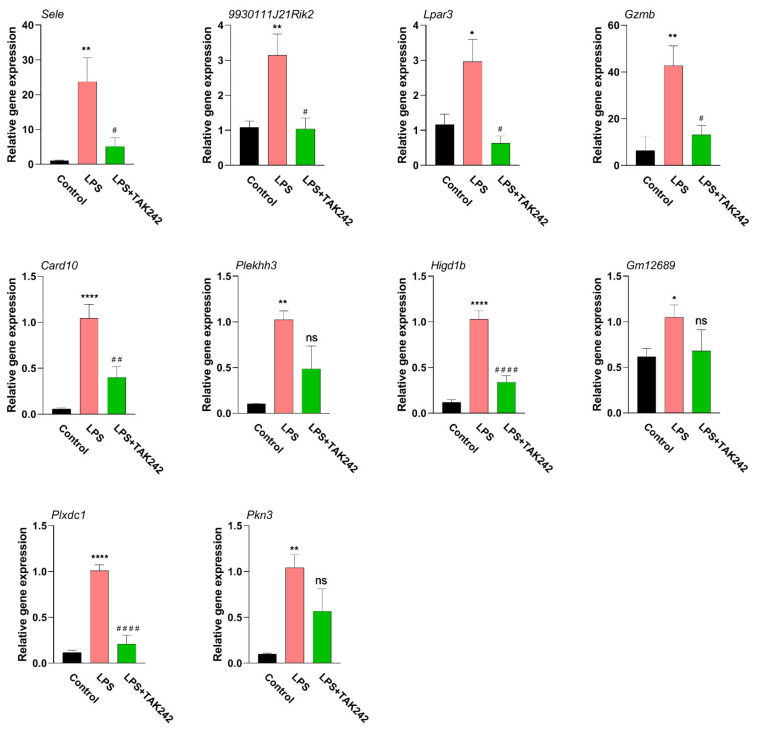
The verification of gene expression of hub genes by qPCR. Total RNA was isolated, followed by a qPCR examination. The gene expression was normalized by GAPDH (*n* = 6). *, significantly different from the control group; * *p* < 0.05; ** *p* < 0.01; **** *p* < 0.0001. #, significantly different from the LPS group; # *p* < 0.05; ## *p* < 0.01; #### *p* < 0.0001. The data were demonstrated as the means ± S.E.M. Statistical significance interpretation was carried out by using the one-way ANOVA followed by the Tukey post hoc tests.

The expression level of hub genes was verified by using qPCR (Figure 10 and Supplementary Table S21). The expression levels of four hub genes were suppressed by TAK 242, including *Sele, 9930111J21Rik2, Lpar3*, *Gzmb*, *Card10, Higd1b,* and *Plxdc1*.

### 2.11. Modeling of Hub Proteins

**Figure 11:**
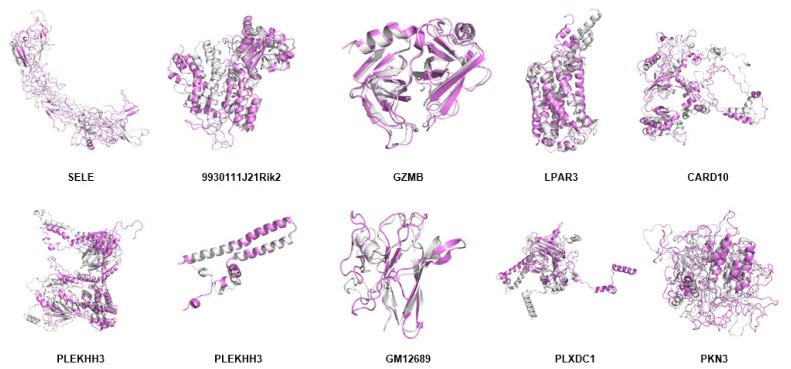
Superposition of the modeled structure (gray) and MD-optimized protein structure (violet) of hub proteins, including SELE, 9930111J21RIK2, GZMB, LPAR3, CARD10, PLEKHH3, HIGD1B, GM12689, PLXDC1, and PKN3. Figures were generated using the Pymol software.

By using the modeling tool Modeller (9v23), the three-dimensional structure of hub proteins was constructed. In total, 1000 models were generated for each protein, which has the lowest DOPE score (Sele: −39,299.40625; 9930111J21Rik2: −49,131.28516; Gzmb: −25,279.35156; Lpar3: −42,574.39453; Pkn3: −61,594.79297). However, there were five proteins that could not be modeled because there were too few homologous templates. Then, the following five protein models were constructed with the help of an online tool-RobeTTAFold (Card10, Plekhh3, Higd1b, Gm12689, and Plxdc1) (Figure 11 and Supplementary Table S19).

**Figure 12:**
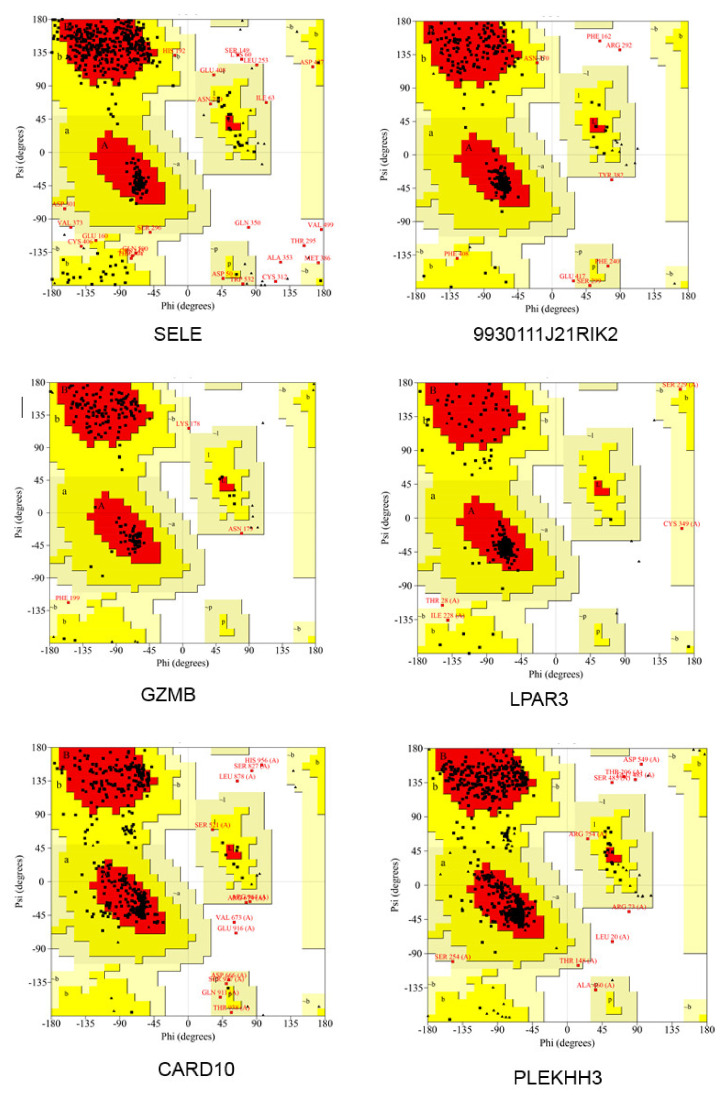

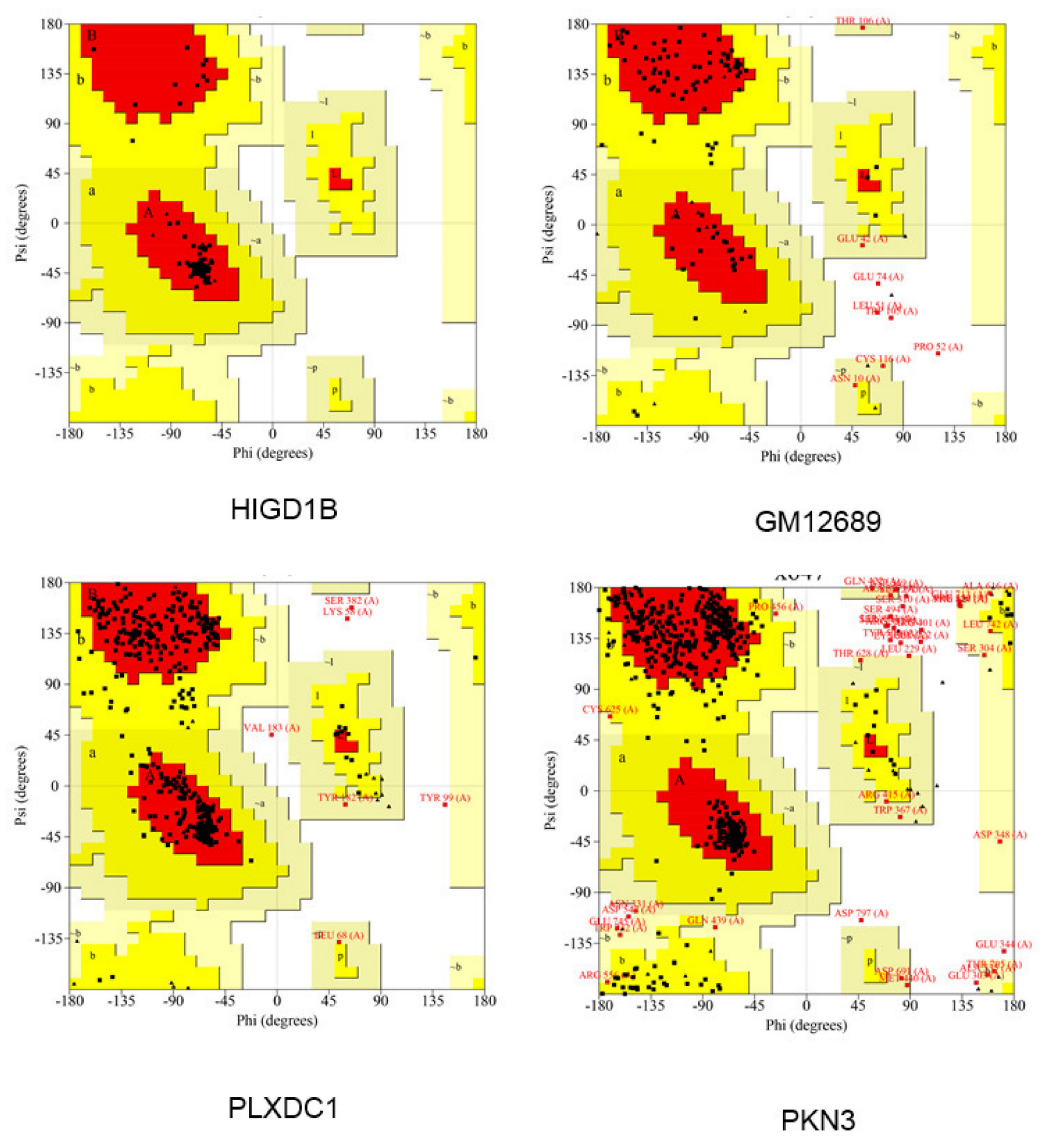
Ramachandran plot of the modeled 3D protein structures of hub proteins. The different colored areas demonstrate ‘generously allowed’ (yellow), ‘disallowed’ (beige), and ‘most favored (red)’ regions.

Next, the quality of the protein model was evaluated using an online tool PROCHECK Ramachandran plots. As shown in Figure 12, the number of bases distributed in the outlier region ranged from 0.0% to 2.5% (Sele: 1.9%; 9930111J21Rik2: 1.0%; Gzmb: 0.0%; Lpar3: 0.0%; Card10: 1.3%; Plekhh3: 1.0%; Higd1b: 0.0%; Gm12689: 3.4%; Plxdc1: 1.0%; Pkn3: 2.5%) (Supplementary Table S20), suggesting that the quality of these templates was acceptable for the subsequent study.

### 2.12. Molecular Dynamics Simulation of Hub Proteins

**Figure 13:**
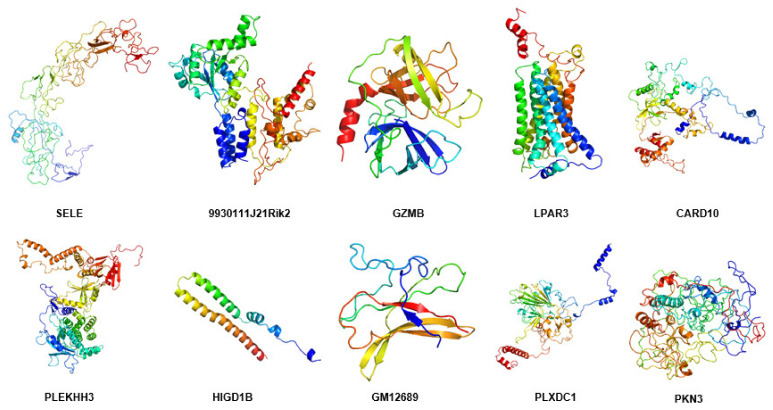
The 3-D view of hub proteins. After molecular dynamics optimization, the three-dimensional view of the hub proteins was demonstrated, including SELE, 9930111J21RIK2, GZMB, LPAR3, CARD10, PLEKHH3, HIGD1B, GM12689, PLXDC1, and PKN3. Figures were generated by using the Pymol software.

**Figure 14:**
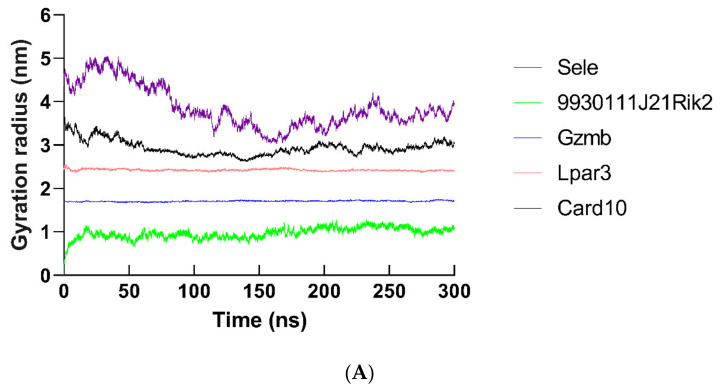

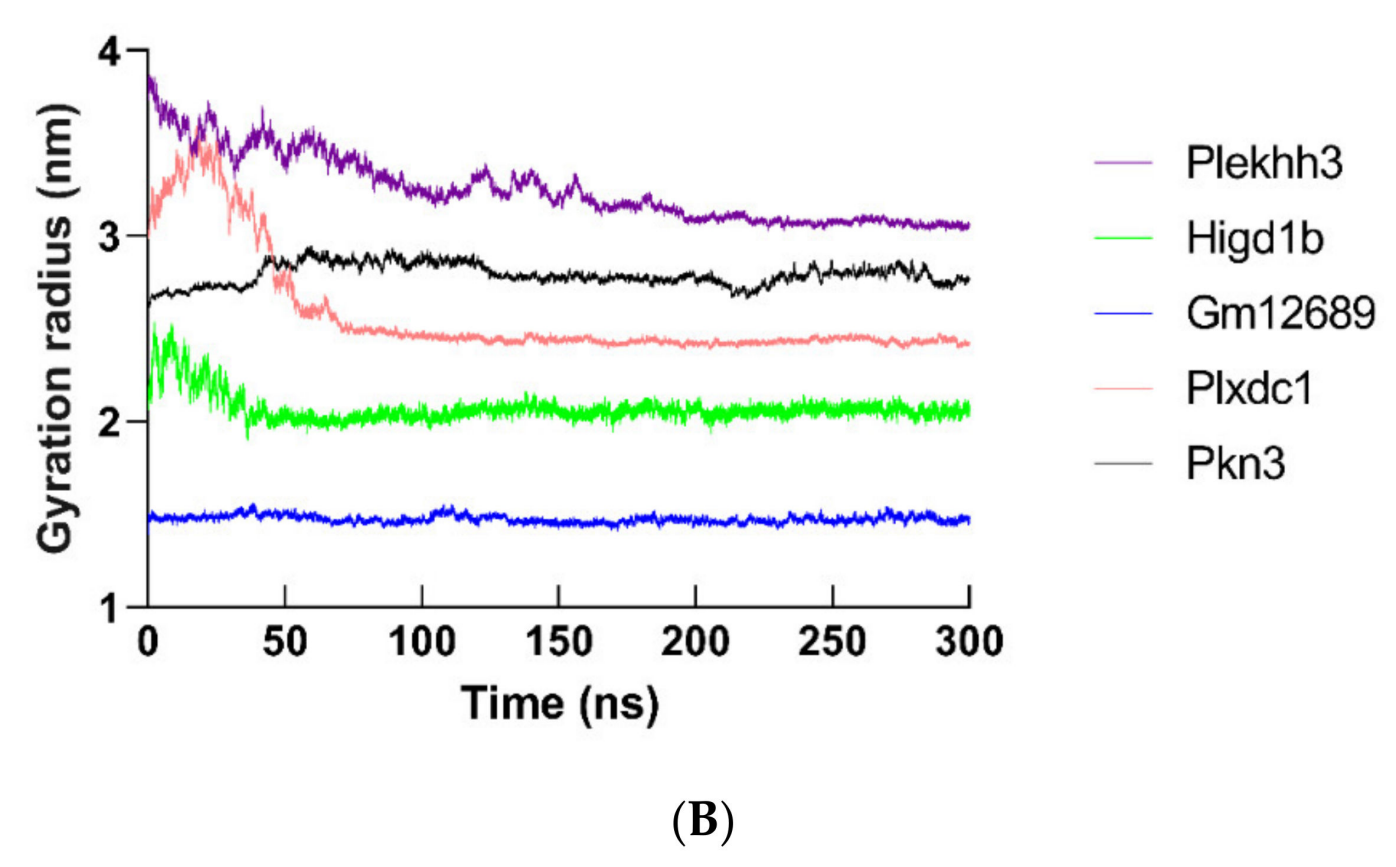
Gyration radius results of hub proteins in molecular dynamics simulation (300 ns). The gyration radius of (**A**)SELE, 9930111J21RIK2, GZMB, LPAR3, CARD10, (**B**) PLEKHH3, HIGD1B, GM12689, PLXDC1, and PKN3 was demonstrated.

**Figure 15:**
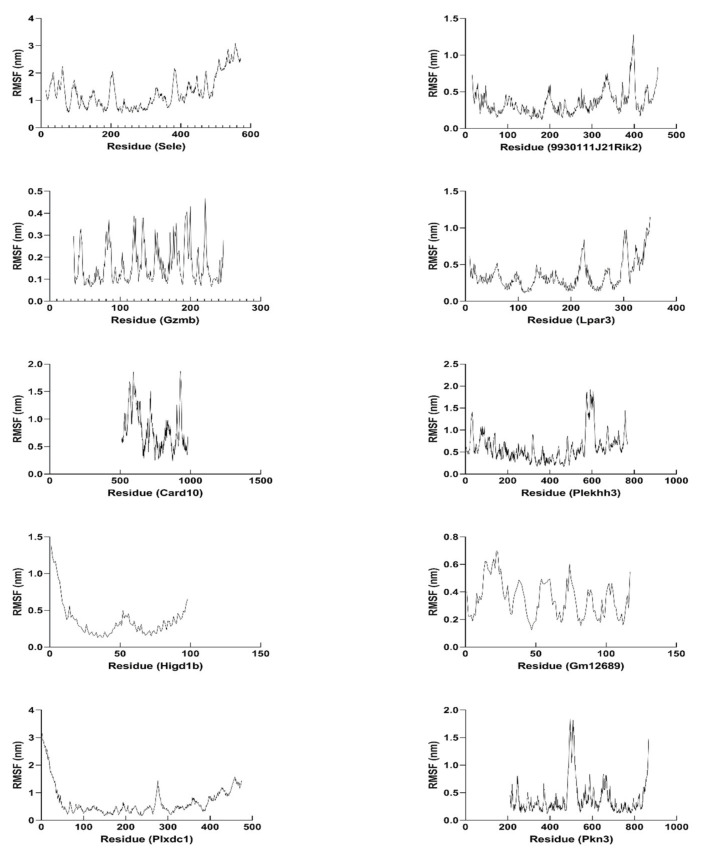
RMSF plots of hub proteins in molecular dynamics simulation (300 ns). The RMSF of SELE, 9930111J21RIK2, GZMB, LPAR3, CARD10, PLEKHH3, HIGD1B, GM12689, PLXDC1, and PKN3 were demonstrated.

**Figure 16:**
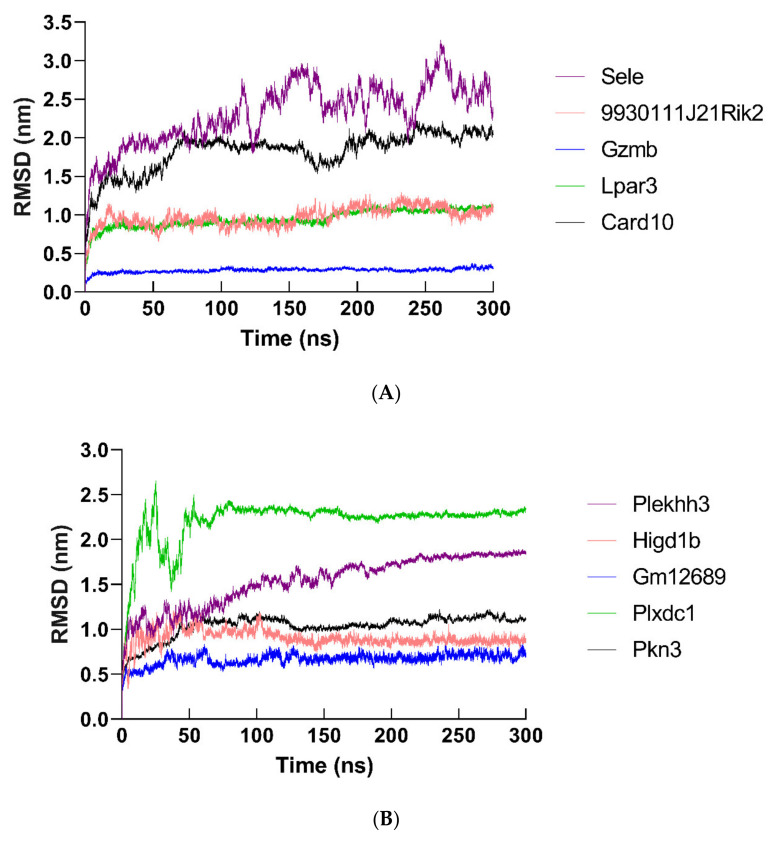
RMSD data of backbone Cα atoms in molecular dynamics simulation (300 ns). RMSD plots of hub proteins were shown, including (**A**) SELE, 9930111J21RIK2, GZMB, LPAR3, CARD10, (**B**) PLEKHH3, HIGD1B, GM12689, PLXDC1, and PKN3.

By using molecular dynamics simulation, the optimized three-dimensional structure of the hub protein was obtained. All proteins were subjected to 300 ns molecular dynamics simulation, and the optimized three-dimensional structure of the protein was finally obtained (Figure 13). Subsequently, a series of parameters related to hub proteins were analyzed, including gyrate (Figure 14), RMSF (Figure 15), and RMSD (Figure 16 and Supplementary Table S23).

The radius of gyration (Rg) is a parameter that measures the tightness of the protein backbone. The increase in Rg indicates that the stability of the protein backbone is enhanced. In our study, the average value of Hub proteins is between 0.9725 and 3.861 (SELE: 3.861; 9930111J21RIK2: 0.9725; GZMB: 1.707; LPAR3: 2.425; CARD10: 2.937; PLEKHH3: 3.253; HIGD1B: 2.070; GM12689: 1.474; PLXDC1: 2.580; PKN3: 2.791) (Figure 14A,B and Supplementary Table S23).

RMSF is a parameter used to evaluate the structural flexibility of hub proteins. The larger the RMSF value of the protein residues, the greater the flexibility. The low RMSF value means that the fluctuation between the residues and the average value is minimal. In our simulation system, the average value of hub proteins fluctuates between 0.3518 and 1.298 (SELE: 1.298; 9930111J21RIK2: 0.3550; GZMB: 0.1658; LPAR3: 0.3541; CARD10: 0.8103; PLEKHH3: 0.6020; HIGD1B: 0.3621; GM12689: 0.3518; PLXDC1: 0.6737; PKN3: 0.3919) (Figure 15 and Supplementary Table S23).

Next, RMSD was analyzed to investigate the structure and dynamics of the hub proteins. The skeleton atoms reach equilibrium from the initial state between 2.54 ns and 6.44 ns (SELE: 4.49 ns; 9930111J21RIK2: 6.44 ns; GZMB: 3.49 ns; LPAR3: 2.45 ns; CARD10: 3.41 ns; PLEKHH3: 4.29 ns; HIGD1B: 2.54ns; GM12689: 2.62 ns; PLXDC1: 2.74 ns; PKN3: 3.68 ns). Subsequently, the structure of the protein began to accumulate at different time points (SELE: 6.81 ns; 9930111J21RIK2: 11.23 ns; GZMB: 7.56 ns; LPAR3: 4.7 ns; CARD10: 9.46 ns; PLEKHH3: 8.69 ns; HIGD1B: 7.22 ns; GM12689: 6.34 ns; PLXDC1: 6.32 ns; PKN3: 9.47 ns). In the end, the structure of these hub proteins remained in a stable state until the molecular dynamics simulation process was completely completed (SELE: 2.34–3.08; 9930111J21RIK2: 1.74–2.17; GZMB: 0.28–0.33; LPAR3: 0.88–1.12; CARD10: 1.62–2.15; PLEKHH3: 1.53–1.85; HIGD1B: 0.94–1.25; GM12689: 0.55–0.75; PLXDC1: 2.06–2.35; PKN3: 1.01–1.28) (Figure 16).

### 2.13. TAK 242-Hub Protein Docking

**Figure 17:**
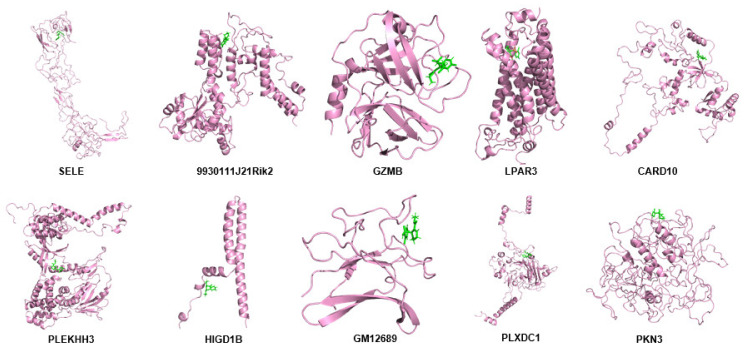
The 3-D view of TAK 242-hub proteins. The three-dimensional view of the TAK242-protein complex was demonstrated, including SELE, 9930111J21RIK2, GZMB, LPAR3, CARD10, PLEKHH3, HIGD1B, GM12689, PLXDC1, and PKN3. TAK 242 was colored in green. Figures were generated by using the Pymol software.

Finally, an online tool, SwissDock, was used to analyze the possible interaction sites between TAK 242 and hub proteins. TAK 242 bound to different sites of the hub protein, where TAK 242 binds to the inside of the cavity of the target proteins (Sele, 9930111J21Rik2, Lpar3, and Card10) or the outside of the target proteins (Gzmb, Plekhh3, Higd1b, Gm12689, Plxdc1, and Pkn3) (Figure 17).

## 3. Discussion

### 3.1. Transcriptomics Study of LPS-Induced Peritonitis

As a clinical challenge that often causes organ damage and death, LPS-induced peritonitis is associated with factors such as peritoneal dialysis, sepsis, and pathogenic microbial infections [2,3,4]. Although some encouraging progress has been achieved, its mechanism of action is still not fully elucidated [1].

Transcriptomics study plays a fundamental role in exploring the mechanism of disease [26], which helps screen candidate genes from a large number of changed genes. To the best of our knowledge, the transcriptomics profiles of LPS-mediated mouse peritonitis have not been reported yet. In the present study, we analyzed the transcriptomic data of mouse peritonitis, which provides clues and an experimental basis for the study of LPS peritonitis.

In this study, LPS triggered 4201 DEGs, including 2442 down-regulated DEGs and 1759 up-regulated DEGs (Figure 3G and Supplementary_1 DEGs_all). Moreover, TLR4 inhibitor TAK 242 was used to investigate the effect of TLR4 on the transcriptomic profile of the peritoneum of LPS-induced peritonitis mice. A total of 30 DEGs were changed by TAK 242, including eight down-regulated DEGs and 22 up-regulated DEGs (Figure 3G and Supplementary_1).

### 3.2. Transcription Factors for Peritonitis Caused by LPS

Transcription factors play a crucial role in regulating various physiological or pathological processes [27]. Hence, the LPS-mediated transcription factor signaling pathway was analyzed in our study. LPS inhibited a series of transcription factor pathways in mouse peritoneal tissue, including *RBPJ, ISL1, TBX1, NKX2-5, GATA2, SNAI1, MYBL2, NFATC1, GATA6*, and *MECP2* (Supplementary Table S15). In contrast, some transcription factor pathways were activated by LPS, such as *NFKB1, STAT1, RELA, JUN, IRF1, REL, SP1, IKBKB, TRP53,* and *IRF8* (Table S16). In our experimental system, the Nfkb transcription factor pathway was significantly activated. This is consistent with previous reports as the Nfkb transcription factor pathway is also activated in the LPS-mediated peritonitis model [14,28]. Hence, the Nfkb transcription factor pathway may play an essential role in the occurrence of LPS-mediated peritonitis.

### 3.3. Hub Genes

In this study, a total of 10 hub genes were obtained, including down-regulated DEGs (*Sele, 9930111J21Rik2, Gzmb*, and *Lpar3*) and up-regulated DEGs (*Card10, Plekhh3, Higd1b, Gm12689, Plxdc1*, and *Pkn3*) (Supplementary Tables S17 and S18).

Sele (Selectin E) is 2912 bp in length and is located on chromosome 1. Sele encodes a protein responsible for mediating the aggregation of white blood cells in inflammation sites, which is a member of the selectin family in the field of cell adhesion molecules. Published reports indicate that Sele is involved in modulating inflammation such as blood leukocytes accumulation, cell adhesion, and endothelium adhesion. It is also anticipated in the disorders of African Tick-Bite Fever, atherosclerosis, and Rheumatoid Vasculitis [29].

The location of 9930111J21Rik2 (RIKEN cDNA 9930111J21 gene 2) is onchromosome 11 and it has a length of 2403 bp. To the best of our knowledge, there is no research on the function of 9930111J21Rik2 yet.

Gzmb (granzyme B) is 1528 bp in length and is located on chromosome 14, which encodes a protein member of the granzyme superfamily. The protein encoded by Gzmb is secreted by cytotoxic T lymphocytes and natural killer cells. Current data show that Gzmb is involved in wound healing and chronic inflammation and is anticipated in the pathology of diseases such as Aggressive Nk-Cell Leukemia and Peripheral T-Cell Lymphoma [30].

Lpar3 (lysophosphatidic acid receptor 3) is located on chromosome 3 and has a length of 2494 bp. Lpar3 encodes a member of the G protein-coupled receptor family. LPAR3 acts as a receptor for lysophosphatidic acid and regulates calcium mobilization. The protein is involved in the GPCR signaling and peptide ligand-binding receptor pathway. Furthermore, LPAR3 is associated with Tracheal cancer and Trachea Adenoid Cystic Carcinoma [31].

Card10 (caspase recruitment domain family, member 10) is 4748 bp and is located on chromosome 15. The protein encoded by Card10 belongs to the membrane-associated guanylate kinase (MAGUK) family, which contains a caspase recruitment domain. Card10 is involved in the apoptosis signaling pathway and is associated with diseases such as Primary Open Angle, Open-Angle Glaucoma, and Glaucoma [32].

Plekhh3 (pleckstrin homology domain-containing family H [with MyTH4 domain] member 3) is located on chromosome 11, with a length of 3007 bp. Existing data show that Plekhh3 plays a role in the process of endothelial tight junction [33].

Higd1b (HIG1 domain family, member 1B) has a length of 735 bp, which is located on chromosome 11. The protein encoded by Higd1b is a member of the hypoxia-inducible gene 1 domain family, naturally expressed on the cell membrane. Higd1b is anticipated in the progression of pituitary adenomas and tumorigenesis [34].

Gm12689 (predicted gene 12689) is a predicted gene with a length of 792 bp. Gm12689 is located on chromosome 4. So far, there is no report regarding the function of Gm12689.

Plxdc1 (plexin domain containing 1) is located on chromosome 11 and has a length of 2922 bp. Plxdc1 is involved in endothelial cell capillary morphogenesis and diseases such as Osteogenic Sarcoma and Mulchandani–Bhoj–Conlin Syndrome [35].

Pkn3 (protein kinase N3) is 2999 bp in length and is located on chromosome 2. Pkn3 contributes to disorders such as Pachyonychia Congenita 1, malignant prostate cancer, and Gastrointestinal Neuroendocrine Tumor [36].

In this study, utilizing bioinformatics and molecular simulation, the possible binding mode between TAK 242 and the above hub proteins was predicted, which is helpful for exploring the anti-LPS-challenged mouse peritonitis of TAK 242. However, in our experimental system, we have difficulties verifying the binding of TKA 242-hub proteins, which requires a series of biochemical experiments to verify.

### 3.4. Anti-Peritonitis Activity of TAK 242 (TLR4 Inhibitor)

TAK 242 (resatorvid) is a specific TLR4 inhibitor that has potent anti-inflammatory activity in a variety of inflammatory diseases [20,21,22,23,24,25], including endotoxemia-induced skeletal muscle wasting [37], sepsis [20,22,24,38], acute kidney injury [39], neuroinflammation [40], acute cerebral ischemia [41], cancer [42], and rheumatoid arthritis [43].

However, to the best of our knowledge, studies on the effect of TAK 242 on the transcriptomics of mouse peritonitis have not been reported yet. In the present study, TAK 242 showed the potency to inhibit the LPS-guided mouse peritonitis model. TAK 242 (5 mg/kg) significantly reduced the levels of inflammatory cytokines in the peritoneal lavage fluid of LPS-induced mice, including IL-6, IFN-γ, IL-1β, NO, and TNF-α. Moreover, TAK 242 changed the expression level of 30 genes in mouse peritoneal tissue at the transcriptome level, including eight down-regulated DEGs and 22 up-regulated DEGs (Figure 3G and Supplementary_1). Furthermore, TAK 242 inhibited LPS-mediated biological adhesion (GO:0022610) (Figure 5C). Therefore, our data provide direct evidence that TAK 242 suppresses LPS-induced mouse peritonitis, which may provide experimental data for the anti-peritonitis activity of TAK 242. In the future, the use of multiple omics methods may provide more clues for in-depth understanding of LPS-induced peritonitis.

Limited by our laboratory resources, we do have difficulties in experimentally verifying the direct interaction between TKA242 and 10 hub proteins. In our study, the molecular docking between TAK 242-hub protein being carried out and the mode of interaction between TAK 242 and hub proteins was initially explored. However, experimental biological data is indispensable in fully revealing the mechanism of TAK 242.

To the best of our knowledge, the study on TAK 242 treatment of peritonitis has not been reported yet. In the present study, TAK 242 significantly inhibited cytokine levels in LPS-induced peritonitis in mice, which provided direct evidence for the anti-inflammatory activity of TAK 242 for peritonitis therapy. Therefore, our data suggest that TAK 242 may be a promising small molecule for treating LPS-triggered peritonitis mice models, which expands the therapeutic indications of TAK 242. Overall, our work sheds light on the exploration of therapeutic approaches that can be used for LPS-induced peritonitis therapy.

## 4. Materials and Methods

### 4.1. Ethical Declaration

All animal protocols were approved by the Animal Experimental Ethical Inspection Council of Northwestern Polytechnical University. All animal experiments were conducted following the guidelines of the European Community guidelines (2010/63/EU).

### 4.2. Mice

Male C57BL/6 mice (6–8 weeks) were acquired from the Animal Experimental Centre of Xi’an Jiaotong University. Mice were housed at 21 ± 2 °C with a 12 h light/dark cycle. Mice have free access to food and water.

### 4.3. Reagents

TRIzol was obtained from Invitrogen™ (Thermo Fisher Scientific, Inc., Waltham, MA, USA). QiaQuick PCR extraction kit was obtained from Qiagen (Venlo, The Netherlands). PrimeScript^TM^ 1st Strand cDNA Synthesis Kit and SYBR^®^ Premix Ex Taq TM II kit were purchased from TaKaRa (Dalian, China). Lipopolysaccharide (LPS) derived from *Escherichia coli* (serotype 0111:B4) was acquired from Sigma (St. Louis, MO, USA). All other reagents were purchased from commercial sources.

### 4.4. LPS-induced Mouse Peritonitis Model

C57BL/6 male mice were divided into three groups (6 mice/group): control group (saline, i.p.), LPS group (LPS 10 mg/kg, i.p.), and TAK 242 + LPS group (TAK 242 5 mg/kg + LPS 10 mg/kg, i.p.). Six hours later, the mice were sacrificed by using carbon dioxide anesthesia. Saline (2 mL/mouse) was injected intraperitoneally into the abdominal cavity of the mouse before the mouse was sacrificed. Subsequently, the mice were placed in a sterile ultra-clean bench for subsequent operations. The peritoneal lavage fluid was aspirated from the abdominal cavity of the mouse, followed by centrifugation (800 rpm, 5 min). Mouse peritoneal tissues were obtained using sterile tissue scissors, followed by liquid nitrogen quick freezing and Trizol treatment for RNA-seq.

### 4.5. ELISA Assay

ELISA assay was conducted as previously described [44]. The cytokine concentration of cytokines (IL-6, IFN-γ, IL-1β, nitric oxide [NO], and TNF-α) in the peritoneal flushing fluid was measured using the mouse ELISA Kit (Beyotime, Shanghai, China) under the manufacturer’s protocol. The mouse peritoneal lavage fluid was collected in a 96-well plate, followed by an ELISA assay, and the OD value at 540/450 nm was measured with a SYNERGY-HT multiwell plate reader (Synergy HT, Bio-Tek Instruments, Winooski, VT, USA). Several standard curves were used to calculate the concentration of cytokines.

### 4.6. Mouse Peritoneal Sample Preparation for RNA-Seq

After the mouse was sacrificed, the mouse’s abdomen was dissected on a sterile, clean bench. The mouse’s peritoneum was cut, followed by liquid nitrogen quick freezing and Tizol soaking. Subsequently, the mouse peritoneal tissue was subjected to RNA-seq.

### 4.7. RNA-Seq

RNA sequencing was performed as previously described [45,46,47]. RNA integrity was detected by using the RNA Nano 6000 Assay Kit (Agilent Technologies, Santa Clara, CA, USA). Libraries for sequencing were constructed by using a NEBNext^®^ UltraTM RNA Library Prep Kit for Illumina^®^ (NEB, Ipswich, MA, USA). The RNA-seq analysis was carried out by Novogene Co Ltd. (Bejing, China).

### 4.8. Bioinformatics Analysis of RNA-Seq Data

#### 4.8.1. Differential Expression Analysis

Differential expression genes (DEGs) were investigated by using the DESeq2 R package (1.16.1). The *p*-values were adjusted with Benjamini and Hochberg’s method to assess the false discovery rate. The criteria for DEGs were adjusted *p*-value < 0.05.

#### 4.8.2. Enrichment Interpretation

The ClusterProfiler R package [48] was employed to investigate the GO enrichment pathways of DEGs (criteria: *p* < 0.05). Also, the DEGs were subjected to other enrichment online tools to explore the biological meaning of the DEGs, including Metascape (http://metascape.org/ accessed on 29 August 2021) [49], PANTHER (http://www.pantherdb.org/ accessed on 29 August 2021) [50], and KEGG (http://www.genome.jp/kegg/ accessed on 29 August 2021).

#### 4.8.3. Protein-Protein Interaction (PPI) Analysis

Subsequently, DEGs were subjected to the STRING database (http://string-db.org accessed on 29 August 2021) [51] to investigate the protein-protein interaction. The criteria were experimentally verified interactions with a combined score above 0.9. Subsequently, the PPI networks were generated by the Cytoscape software (Version 3.7.2). With the help of a Cytoscape plug-in Molecular Complex Detection (MCODE), the modules of the PPI network were acquired.

Two plug-ins, the clueGO (http://apps.cytoscape.org/apps/cluego accessed on 29 August 2021) [52] and Cluepedia (http://apps.cytoscape.org/apps/cluepedia accessed on 29 August 2021) [53], were employed to enrich the biological pathways of DEGs (criteria: *p* < 0.05 and kappa coefficient > 0.9).

### 4.9. Prediction of Transcriptional Factors for DEGs

An online tool TRRUST (Version 2) (https://www.grnpedia.org/trrust/ accessed on 29 August 2021) was employed to predict the transcription factor pathways of DEGs.

### 4.10. Real-Time Quantitative PCR (qPCR) Detection

Total RNA was extracted from mouse peritoneal samples as previously described [54,55]. The single-stranded cDNA was transcribed by using a PrimeScript^TM^ 1st Strand cDNA Synthesis Kit. Then, qPCR was conducted to test the gene expression of *Sele, 9930111J21Rik2, Gzmb, Lpar3, Card10, Plekhh3, Higd1b, Gm12689, Plxdc1*, and *Pkn3* with the SYBR^®^ Premix Ex Taq TM II system (Sigma Aldrich, St. Louis, MO, USA). The qPCR primers were displayed in Supplementary Table S21. Gene level was normalized to a reference gene (GAPDH). Data were determined by the comparative 2^−ΔΔ^CT approach.

### 4.11. Modeling of Hub Proteins

Modeller (9v23) [56] was employed to generate the three-dimensional structures of Sele, 9930111J21Rik2, Gzmb, Lpar3, and Pkn3. Briefly, protein models were retrieved from the PDB database [57] (Supplementary Table S19). For each protein, three templates with query cover above 35% were used for homology modeling. Next, modeling modules (Salign, Align2d, and Model) were employed to construct protein models. For every hub protein, a total of 1000 models were generated, and the model with the lowest DOPE (discrete optimized protein energy) score was selected for subsequent study.

Due to the lack of sufficient protein templates for homology modeling, an online tool, RobeTTAFold [58], was used to construct the three-dimensional structures of the following five proteins, including Card10, Plekhh3, Higd1b, Gm12689, and Plxdc1.

An online PROCHECK Ramachandran plots (http://www.ebi.ac.uk/thornton-srv/databases/pdbsum/Generate.html accessed on 8 September 2021) [58] was used to examine the quality of the models.

### 4.12. Molecular Dynamics Simulation

Molecular dynamics simulation was carried out by using Gromacs 2018.12 [59]. The CHARMM36 force field was used [60]. After the protein was solvated with TIP3P water models, Na^+^ or Cl^−^ ions were used to neutralize the whole system. Periodic boundary conditions (PBC) were employed in all directions. At the energy minimization step, the steepest descent (50,000 steps) with the max force less than 100 KJ/mol was introduced in the system. Then, NVT (298.15 K, 100 ps) and NPT (298.15 K, 1.0 bar, 200 ps) were performed, respectively. For each hub protein, molecular dynamics was conducted for 300 ns.

Molecular dynamics trajectory was analyzed by Gromacs utilities to acquire a series of parameters such as RMSF (root mean square fluctuation), gyration (Rg), and RMSD (root mean square deviation). All protein structures were drawn with the Pymol software (Delano, W.L. The Pymol Molecular Graphics System (2002) DeLano Scientific, San Carlos, CA, USA. http://www.pymol.org accessed on 24 August 2021).

### 4.13. TAK 242-hub Protein Docking

The 3-D structure of TAK 242 was retrieved from the PubChem (NCBI) website (https://pubchem.ncbi.nlm.nih.gov/ accessed on 29 July 2021). The 3-D structure of hub proteins acquired from molecular dynamics was used in the docking study. Both TAK 242 structure and hub proteins were subjected to the online tool SwissDock (http://www.swissdock.ch/ accessed on 1 August 2021) for virtual docking [61,62].

### 4.14. Statistical Interpretation

Statistical analysis was conducted by the GraphPad Prism Software Version 8.0 (San Diego, CA, USA). The data were demonstrated as means ± standard error of the mean (S.E.M) after being analyzed by normality distribution. Data were interpreted with one-ANOVA followed by the post hoc tests (Tukey). *p* < 0.05 were considered statistically significant.

## 5. Conclusions

Our data investigated the transcriptomic profiles of the peritoneum of mice with LPS-induced peritonitis. Moreover, the anti-inflammatory activity and transcriptomics profile of the TLR4 inhibitor TAK 242 against LPS-induced peritonitis were also studied. Our data provide clues for understanding the pathogenesis of LPS-induced peritonitis in mice.

## Data Availability

Not applicable.

## Supplementary Materials

The following are available online at https://www.mdpi.com/article/10.3390/ijms222313008/s1.
