# Supplementary material for: Transcriptomics Changes in the Peritoneum of Mice with Lipopolysaccharide-Induced Peritonitis
Source: Int J Mol Sci. 2021 Nov 30;22(23):13008. doi: 10.3390/ijms222313008 (PMC8657704; doi:10.3390/ijms222313008)
Supplement: Supplementary file 1 [file ijms-22-13008-s001.zip › ijms-1473149-supplementary/Supplementary_10_Table S9. Details of DEGs-lincRNA (TAK vs. LPS).pdf]

International Journal of
Molecular Sciences

MDPI

Table S9. Details of DEGs-lincRNA (TAK vs. LPS)

Gene_symbol	log2FoldChange	Padj	Gene_chrosome	Change
Gm49539	3.01685556	0.040316569	15	Up
