# Supplementary material for: Transcriptomics Changes in the Peritoneum of Mice with Lipopolysaccharide-Induced Peritonitis
Source: Int J Mol Sci. 2021 Nov 30;22(23):13008. doi: 10.3390/ijms222313008 (PMC8657704; doi:10.3390/ijms222313008)
Supplement: Supplementary file 1 [file ijms-22-13008-s001.zip › ijms-1473149-supplementary/Supplementary_11_Table S10. Details of DEGs-miRNA genes (LPS vs. Ctrl).pdf]

International Journal of
Molecular Sciences

MDPI

Table S10. Details of DEGs-miRNA genes (LPS vs. Ctrl)

Gene_symbol	log2FoldChange	Padj	Gene_chrosome	Change
Gm24084	-4.216698883	0.007277862	10	Down
Mir196a-1	-3.524444858	0.018465223	11	Down
Mir6929	-3.399847395	0.006950872	11	Down
Gm22991	-3.323259379	0.048348057	4	Down
Mir3091	4.471300483	0.016301898	2	Up
Gm44340	4.2355963	0.007140473	14	Up
Snord43	4.204019183	0.038263299	15	Up
Gm25911	3.981174504	5.71E-14	1	Up
Gm24017	3.550623839	0.00010253	13	Up
Mir6386	3.496899719	0.015770162	9	Up
Gm27514	2.89202697	0.002592878	6	Up
Mir7678	2.272326192	0.002359836	2	Up
Mir1898	2.20486487	0.013002107	15	Up
Mir7-1	1.65010275	0.000213484	13	Up
