# Supplementary material for: Transcriptomics Changes in the Peritoneum of Mice with Lipopolysaccharide-Induced Peritonitis
Source: Int J Mol Sci. 2021 Nov 30;22(23):13008. doi: 10.3390/ijms222313008 (PMC8657704; doi:10.3390/ijms222313008)
Supplement: Supplementary file 1 [file ijms-22-13008-s001.zip › ijms-1473149-supplementary/Supplementary_12_Table S11. Details of DEGs-antisense genes (LPS vs. Ctrl).pdf]

International Journal of
Molecular Sciences

MDPI

Table S11. Details of DEGs-antisense genes (LPS vs. Ctrl)

Gene_symbol	log2FoldChange	Padj	Gene_chrosome	Change
B630019A10Rik	-5.062036513	0.000510177	16	Down
Gm12963	-5.053940861	2.94E-07	4	Down
Kcnd3os	-5.035819698	4.15E-05	3	Down
B430119L08Rik	-4.817589499	1.68E-07	12	Down
Gm13111	-4.733873261	6.85E-05	4	Down
4933417O13Rik	-4.680011015	0.000189867	7	Down
Gm45449	-4.631372621	0.00089975	8	Down
9530036M11Rik	-4.543142343	4.98E-05	6	Down
Gm13919	-4.381290958	0.000456952	2	Down
Gm671	-4.252130865	0.000295912	15	Down
Gm30270	-4.175550012	0.002018414	6	Down
Gm17025	-4.109591842	0.000366515	15	Down
Gm12426	-4.035209586	0.003038629	4	Down
Gm11342	-4.023241356	0.032704175	13	Down
Gm48495	-3.977477379	0.001021169	10	Down
Gm4419	-3.909101063	0.002023171	12	Down
Gm11767	-3.552910521	0.014548001	11	Down
Gm12002	-3.499703234	2.20E-16	11	Down
A530020G20Rik	-3.357419415	1.23E-14	3	Down
Sox5os4	-3.330881977	0.03273576	6	Down
Gm30329	-3.305404983	0.007800475	8	Down
Bloc1s6os	-3.233655168	0.031705121	2	Down
Gm45447	-3.231022841	0.000856674	2	Down
Chn1os3	-3.225886898	0.016711252	2	Down
Gm28932	-3.220616953	0.010790775	14	Down
A230107N01Rik	-3.207934724	0.040609297	13	Down
Gm10561	-3.159591053	1.27E-05	1	Down
B430010I23Rik	-3.079410097	1.36E-06	8	Down
Gm14964	-3.05506457	6.31E-12	19	Down
Gm12454	-3.027397493	0.005980354	4	Down
Gm45774	8.861796087	9.23E-42	8	Up
Gm11738	7.554591172	2.11E-14	11	Up
A630012P03Rik	7.166987503	1.69E-09	X	Up
4930430E12Rik	7.007404235	7.68E-11	4	Up
Gm16685	6.134628324	4.11E-27	3	Up
Gm29292	5.828014128	3.22E-08	11	Up
Gm12530	5.392644388	6.28E-06	4	Up
Gm47595	5.378389245	1.54E-06	10	Up
Gm15556	5.268146447	7.83E-06	17	Up
Gm44672	5.027995094	6.82E-09	7	Up
Gm12868	4.737500788	0.000295006	4	Up
1700025N23Rik	4.66592184	3.43E-08	6	Up

Gm20496	4.650021446	0.000217919	17	Up
1700071M16Rik	4.637553526	0.000130721	17	Up
Gm14236	4.540800685	0.002326701	2	Up
Gm27040	4.529016409	0.000386633	7	Up
Gm15731	4.508144122	0.002136502	5	Up
Gm16015	4.40863417	0.000324143	5	Up
Gm13822	4.377428724	9.16E-21	5	Up
Gm42809	4.374457278	1.40E-24	3	Up
AC127341.3	4.257404136	1.31E-10	17	Up
Gm17173	4.127266438	0.018647401	14	Up
Gm47118	4.11414052	0.007954635	13	Up
Gm15943	4.018130432	1.09E-25	15	Up
BC018473	3.937895954	0.048788303	11	Up
Gm16098	3.862464113	3.16E-05	2	Up
Gm20275	3.829720408	0.02694002	9	Up
Gm11626	3.750672028	0.017472883	11	Up
Gm44851	3.627010143	0.021608123	7	Up
Gm17089	3.564468914	0.000315954	6	Up

Note: down-regulated and up-regulated 30 antisense genes were displayed.
