# Supplementary material for: Transcriptomics Changes in the Peritoneum of Mice with Lipopolysaccharide-Induced Peritonitis
Source: Int J Mol Sci. 2021 Nov 30;22(23):13008. doi: 10.3390/ijms222313008 (PMC8657704; doi:10.3390/ijms222313008)
Supplement: Supplementary file 1 [file ijms-22-13008-s001.zip › ijms-1473149-supplementary/Supplementary_13_Table S12. KEGG enrichment pathways of down-regulated DEGs (LPS vs. Control).pdf]

International Journal of
Molecular Sciences

MDPI

Table S12. KEGG enrichment pathways of down-regulated DEGs (LPS vs. Control)

	Pathway ID	Name	Gene count	PValue	Genes
1	mmu05200	Pathways in cancer	159	0.000184052	*Gstt3, Pik3r2, Calm2, Adcy7, Fzd7, Mapk3, Gstt2, Rasgrp2, Fzd2, Plekhg5, Plcg1, Rasgrp4, Epor, Gli1, Gli2, E2f1, Fgf18, Pparg, Hey1, Bbc3, Runx1t1, Fgf10, Gng11, Kitl, Pdgfb, Flt3l, Tgfb3, Gstt1, Csf1r, Adcy6, Cebpa, Rasgrp3, Plcb2, Tcf7l1, Heyl, Bmp4, Fgf16, Adcy5, Plcg2, Dvl2, Wnt9a, Cdkn1b, Axin2, Bad, Ptch2, Jag2, Gna11, Smo, Ccnd3, Fgfr1, Gng7, Col4a4, Tcf7l2, Ret, Wnt6, Gna12, Prkcb, Fgfr4, Gng8, Pik3cb, Casp9, Lpar4, Wnt5b, Apc2, Igf1, Esr1, Ddb2, Cxcr4, E2f2, Plcb3, Pik3r3, Rara, Brca2, Cxcl12, Fzd10, Gsta3, Tgfb1, Cks1b, Mmp9, Itga6, Wnt7b, Hhip, Rarb, Ptch1, Adcy1, Traf5, Lrp6, Msh2, Flt3, Gnb5, Itga2b, Ccne2, Lpar5, Arhgef1, Il4, Gsta4, Agtr1a, Gng2, Kif7, Plcb1, Rxra, Cdk2, Bdkrb2, Mgst2, Calm3, Il7, Fgfr2, Fgf7, Kras, Gngt2, Col4a3, Wnt2, Adcy3, Gstm1, Dvl3, Wnt2b, Prkacb, Calml4, Gsto2, Col4a6, Ar, Gnai1, Gnb2, Rac3, Ctbp2, Gstm6, Tert, Traf4, Rps6ka5, Mlh1, Il12a, Skp2, Dapk2, Wnt10b, Fzd3, Peg12, Ccnd1, Rb1, Wnt11, Gnai2, Jag1, Dll4, Abl1, Pik3r1, Rad51, Lamb3, Gnb3, Ncoa3, Gsta2, Notch3, Akt1, Polk, Hes1, Mapk9, Mmp2, Wnt16, Fgf4, Fgf8, Lamc3*
2	mmu05165	Human papillomavirus infection	99	0.024043548	*Pik3r2, Fzd7, Mapk3, Mfng, Fzd2, E2f1, Tyk2, Itga11, Hey1, Ppp2r5a, Hdac5, Atp6v0a4, Tcf7l1, Heyl, Lfng, Hes6, Irf3, Dvl2, Wnt9a, Cdkn1b, Axin2, Itgb5, Bad, Prkcz, Itga8, Hdac8, Maml3, Itgb7, Ccnd3, Col4a4, Atp6v0d2, Tcf7l2, Prkci, Wnt6, Ppp2r2b, Pik3cb, Wnt5b, Apc2, Itgb4, Hdac11, Creb1, Thbs2, Pik3r3, Fzd10, Col6a6, Itga6, Wnt7b, Itga2b, Ccne2, Dlg2, Tada3, Hdac10, Ppp2r5d, Dlg3, Cdk2, Creb3l3, Rbl2, Kras, Hdac7, Col4a3, Wnt2, Rfng, Hes2, Tradd, Tnxb, Maml2, Dvl3, Wnt2b, Atp6v1a, Prkacb, Col4a6, Magi1, Crb3, Chd4, Tert, Col6a3, Ppp2r5c, Tubg2, Vtn, Wnt10b, Fzd3, Ccnd1, Chad, Col9a1, Rb1, Patj, Wnt11, Jag1, Pik3r1, Lamb3, Pard6a, Creb5, Notch3, Akt1, Hes1, Wnt16, Scrib, Lamc3, Ppp2r5b*

3	mmu04360	Axon guidance	75	3.99E-07	*Pik3r2, Dpysl2, Mapk3, Plcg1, Ngef, Efna1, Ephb3, Nfatc2, Plxna3, Tbc1d24, Ephb4, Efna2, Rras, Plcg2, Nfatc4, Ablim1, Prkcz, Smo, Fyn, Epha1, Efna4, Lrrc4c, Pik3cb, Wnt5b, Nrp1, Efnb1, Cxcr4, Pik3r3, Rgs3, Ryk, Cxcl12, Ntn4, Boc, Dpysl5, Ephb1, Ptch1, Ephb6, Sema6a, Bmp7, Sema4a, Epha4, Limk1, Robo1, Efnb3, Slit2, Sema6c, Plxna4, Plxnc1, Rasa1, Plxnb1, Ntng1, Nfatc3, Kras, Trpc3, Pak1, Gnai1, Rac3, Cdk5, Sema3c, Sema3b, Srgap2, Sema3g, Plxnb2, Fzd3, Ablim3, Nck1, Sema3d, Gnai2, Abl1, Pik3r1, Rhod, Fes, Pard6a, Unc5b, L1cam*
4	mmu04014	Ras signaling pathway	73	0.006641821	*Pik3r2, Calm2, Mapk3, Rasgrp2, Rasgrf2, Plcg1, Rasgrp4, Fgf18, Ntf3, Pla2g2d, Efna1, Fgf10, Gng11, Kitl, Pdgfb, Flt3l, Gab1, Csf1r, Efna2, Kdr, Rasgrp3, Fgf16, Angpt4, Rras, Plcg2, Bad, Syngap1, Efna4, Bdnf, Fgfr1, Gng7, Rgl2, Prkcb, Fgfr4, Gng8, Pik3cb, Pld2, Igf1, Pik3r3, Tek, Pdgfd, Flt3, Gnb5, Rasal3, Grin2a, Gng2, Rasa1, Rasal2, Calm3, Fgfr2, Fgf7, Kras, Gngt2, Prkacb, Calml4, Pak1, Gnb2, Rac3, Pla2g12b, Ngfr, Pla2g2e, Rab5b, Abl1, Plce1, Pik3r1, Pla2g4b, Gnb3, Foxo4, Akt1, Mapk9, Rin1, Fgf4, Fgf8*
5	mmu04015	Rap1 signaling pathway	70	0.002937796	*Id1, Pik3r2, Calm2, Adcy7, Mapk3, Rasgrp2, Plcg1, Fgf18, Efna1, Fgf10, Kitl, Pdgfb, Csf1r, Adcy6, Efna2, Kdr, Rasgrp3, Plcb2, Fgf16, Adcy5, Angpt4, Rras, Prkcz, P2ry1, Efna4, Fgfr1, Prkci, Prkcb, Fgfr4, Pik3cb, Lpar4, Igf1, Plcb3, Pik3r3, Tek, Rapgef3, Prkd3, Prkd1, Adcy1, Sipa1, Pdgfd, Itga2b, Lpar5, Grin2a, Prkd2, Plcb1, Calm3, Fgfr2, Fgf7, Kras, Adcy3, Calml4, Magi1, Gnai1, Rac3, Rgs14, Vasp, Ngfr, Rapgef4, Gnai2, Plce1, Pik3r1, Rap1gap, Magi3, Pard6a, Tln1, Akt1, Itgb2, Fgf4, Fgf8*
6	mmu04024	cAMP signaling pathway	67	0.000936278	*Pik3r2, Hcar1, Calm2, Adcy7, Mapk3, Gli1, Npy1r, Oxtr, Adcy6, Pde3b, Lipe, Atp1b1, Adcy5, Rras, Cftr, Adcyap1r1, Bad, Adrb1, Nfatc1, Myl9, Bdnf, Fxyd2, Acox3, Pik3cb, Pld2, Atp1a3, Sucnr1, Creb1, Cngb1, Pik3r3, Vipr2, Rapgef3, Hhip, Ptch1, Adcy1, Gria3, Atp2b2, Grin2a, Creb3l3, Vav3, Pde4c, Calm3, Vav2, Adcy3, Prkacb, Calml4, Pak1, Gnai1, Rac3, Grin2d, Hcn2, Gabbr1, Ryr2, Pde4a, Rapgef4, Cnga4, Gnai2, Plce1, Pik3r1, Gria4, Creb5, Akt1, Atp2b1, Npr1, Mapk9, Ffar2, Grin3a*
7	mmu04022	cGMP-PKG signaling pathway	64	0.000129365	*Irs3, Adrb3, Calm2, Adcy7, Mapk3, Gucy1b1, Nfatc2, Adcy6, Pde3b, Pde5a, Plcb2, Atp1b1, Adcy5, Gucy1a1, Irs1, Adra2b, Mylk3, Nfatc4, Kcnj8, Bad, Adrb1, Nfatc1,*

					Myl9, Gna11, Fxyd2, Gna12, Gtf2i, Atp1a3, Creb1, Cngb1, Plcb3, Adcy1, Mrvi1, Atp2b2, Atp2a3, Npr2, Prkg2, Agtr1a, Nos3, Itpr3, Adra1b, Plcb1, Creb3l3, Bdkrb2, Gucy1a2, Calm3, Nfatc3, Mylk4, Adcy3, Gtf2ird1, Calml4, Pik3cg, Gnai1, Itpr1, Vasp, Gnai2, Creb5, Akt1, Atp2b1, Npr1, Adra2c, Adra1a, Srf, Gata4
8	mmu05225	Hepatocellular carcinoma	58	0.006641821	*Gstt3, Pik3r2, Fzd7, Mapk3, Gstt2, Fzd2, Plcg1, E2f1, Smarca2, Tgfb3, Gab1, Gstt1, Tcf7l1, Plcg2, Dvl2, Wnt9a, Smarcd2, Axin2, Bad, Smarcc2, Tcf7l2, Wnt6, Prkcb, Pik3cb, Wnt5b, Apc2, Ddb2, E2f2, Pik3r3, Fzd10, Gsta3, Tgfb1, Wnt7b, Lrp6, Gsta4, Mgst2, Kras, Wnt2, Gstm1, Dvl3, Arid1a, Wnt2b, Gsto2, Gstm6, Tert, Dpf1, Wnt10b, Fzd3, Peg12, Ccnd1, Rb1, Wnt11, Pik3r1, Gsta2, Arid2, Akt1, Polk, Wnt16*
9	mmu04934	Cushing syndrome	58	0.000471319	*Adcy7, Kcnk2, Fzd7, Mapk3, Fzd2, Cdkn2c, E2f1, Pbx1, Adcy6, Star, Plcb2, Tcf7l1, Kcnk3, Adcy5, Dvl2, Wnt9a, Cdkn1b, Axin2, Gna11, Scarb1, Tcf7l2, Wnt6, Pde8a, Wnt5b, Apc2, Wdr5b, Creb1, E2f2, Plcb3, Cacna1h, Fzd10, Wnt7b, Adcy1, Men1, Ccne2, Agtr1a, Itpr3, Plcb1, Cdk2, Creb3l3, Wnt2, Adcy3, Dvl3, Wnt2b, Prkacb, Gnai1, Pde8b, Itpr1, Wnt10b, Fzd3, Ccnd1, Rb1, Wnt11, Gnai2, Nceh1, Creb5, Wnt16, Cacna1g*
10	mmu05224	Breast cancer	52	0.003664137	*Pik3r2, Fzd7, Mapk3, Fzd2, E2f1, Fgf18, Hey1, Fgf10, Tcf7l1, Heyl, Fgf16, Dvl2, Wnt9a, Axin2, Jag2, Fgfr1, Tcf7l2, Wnt6, Pik3cb, Wnt5b, Apc2, Igf1, Esr1, Ddb2, E2f2, Pik3r3, Brca2, Fzd10, Wnt7b, Lrp6, Fgf7, Kras, Wnt2, Dvl3, Wnt2b, Wnt10b, Fzd3, Peg12, Ccnd1, Rb1, Wnt11, Jag1, Dll4, Pik3r1, Ncoa3, Notch3, Akt1, Polk, Hes1, Wnt16, Fgf4, Fgf8*
11	mmu05226	Gastric cancer	51	0.006641821	*Pik3r2, Fzd7, Mapk3, Fzd2, E2f1, Fgf18, Fgf10, Tgfb3, Gab1, Tcf7l1, Fgf16, Dvl2, Wnt9a, Cdkn1b, Axin2, Tcf7l2, Wnt6, Pik3cb, Wnt5b, Apc2, Ddb2, E2f2, Pik3r3, Fzd10, Tgfb1, Wnt7b, Rarb, Lrp6, Ccne2, Rxra, Cdk2, Fgfr2, Fgf7, Kras, Wnt2, Dvl3, Wnt2b, Tert, Mlh1, Wnt10b, Fzd3, Peg12, Ccnd1, Rb1, Wnt11, Pik3r1, Akt1, Polk, Wnt16, Fgf4, Fgf8*
12	mmu04390	Hippo signaling pathway	50	0.021493881	*Id1, Fzd7, Fzd2, Gli2, Nkd1, Bbc3, Tgfb3, Smad7, Tcf7l1, Bmp4, Tead2, Dlg4, Dvl2, Frmd6, Ajuba, Wnt9a, Axin2, Prkcz, Ccnd3, Bmp5, Tcf7l2, Prkci, Wnt6, Ppp2r2b, Wnt5b, Apc2, Snai2, Fzd10, Tgfb1, Wnt7b, Dlg2, Bmp7, Dlg3, Bmpr1a, Rassf6,*

					Crb2, Wnt2, Wtip, Dvl3, Wnt2b, Lats2, Wnt10b, Fzd3, Ccnd1, Patj, Wnt11, Pard6a, Wnt16, Itgb2, Scrib
13	mmu04310	Wnt signaling pathway	50	0.005462865	*Fzd7, Fzd2, Dkk2, Nkd1, Ctnnbip1, Nfatc2, Invs, Plcb2, Tcf7l1, Nfatc4, Dvl2, Wnt9a, Axin2, Nfatc1, Ccnd3, Tcf7l2, Vangl2, Wnt6, Prkcb, Wnt5b, Apc2, Plcb3, Fzd10, Vangl1, Wnt7b, Sfrp2, Lrp6, Cxxc4, Nkd2, Sfrp4, Plcb1, Nfatc3, Wnt2, Dvl3, Wnt2b, Prkacb, Bambi, Rac3, Nlk, Ctbp2, Wisp1, Wnt10b, Fzd3, Peg12, Ccnd1, Wnt11, Daam2, Sfrp1, Mapk9, Wnt16*
14	mmu04921	Oxytocin signaling pathway	48	0.033303204	*Calm2, Adcy7, Mapk3, Gucy1b1, Nfatc2, Oxtr, Cacnb4, Camk1d, Adcy6, Plcb2, Adcy5, Gucy1a1, Camk1, Mylk3, Nfatc4, Nfatc1, Myl9, Prkcb, Plcb3, Adcy1, Npr2, Nos3, Itpr3, Eef2k, Plcb1, Gucy1a2, Calm3, Ppp1r12c, Nfatc3, Kras, Mylk4, Adcy3, Cacnb3, Prkacb, Calml4, Pik3cg, Gnai1, Ryr2, Itpr1, Kcnj14, Ccnd1, Gnai2, Prkag2, Pla2g4b, Npr1, Camk1g, Prkaa1, Kcnj4*
15	mmu04371	Apelin signaling pathway	48	0.008721473	*Calm2, Adcy7, Mapk3, Plin1, Gng11, Hdac5, Aplnr, Adcy6, Pde3b, Lipe, Plcb2, Adcy5, Rras, Mylk3, Gng7, Gng8, Apln, Plcb3, Adcy1, Gnb5, Agtr1a, Nos3, Gng2, Itpr3, Plcb1, Calm3, Kras, Mylk4, Gngt2, Adcy3, Prkacb, Calml4, Pik3cg, Gnai1, Gnb2, Ryr2, Klf2, Itpr1, Ccnd1, Gnai2, Jag1, Prkag2, Gnb3, Notch3, Akt1, Pik3r4, Prkaa1, Acta2*
16	mmu04550	Signaling pathways regulating pluripotency of stem cells	47	0.006238091	*Id1, Pik3r2, Acvr1c, Fzd7, Mapk3, Id4, Fzd2, Pcgf2, Tcf3, Meis1, Bmp4, Dvl2, Wnt9a, Acvr1, Axin2, Smad9, Fgfr1, Wnt6, Fgfr4, Pik3cb, Wnt5b, Apc2, Igf1, Pik3r3, Lifr, Fzd10, Isl1, Myf5, Wnt7b, Klf4, Lefty2, Bmpr1a, Inhba, Fgfr2, Kras, Wnt2, Dvl3, Wnt2b, Wnt10b, Fzd3, Jarid2, Wnt11, Pik3r1, Akt1, Bmi1, Wnt16, Acvr2b*
17	mmu04142	Lysosome	46	0.003664137	*Galc, Dnase2a, Pla2g15, Atp6v0a4, Lipa, Gga2, Gusb, Laptm5, Hgsnat, Atp6v0d2, Arsg, Asah1, Ctns, Ap3m2, Nagpa, Ids, Slc17a5, Acp2, Sgsh, Hyal1, Arsa, Abcb9, Hexa, Sumf1, Abca2, Ctsf, Ctsk, Manba, Cln3, Idua, Ppt1, Gm2a, Ctso, Naglu, Psap, Clta, Ctsh, Ctsa, Fuca1, Glb1, Ap1s1, Ap3b2, Igf2r, Tpp1, Galns, Ap3s1*
18	mmu04926	Relaxin signaling pathway	44	0.016968382	*Pik3r2, Adcy7, Mapk3, Arrb2, Gng11, Gna15, Rxfp2, Adcy6, Plcb2, Adcy5, Prkcz, Gng7, Col4a4, Gng8, Pik3cb, Creb1, Plcb3, Pik3r3, Tgfb1, Mmp9, Adcy1, Gnb5, Arrb1, Nos3, Gng2, Plcb1, Creb3l3, Kras, Gngt2, Col4a3, Adcy3, Rxfp1, Prkacb,*

					Col4a6, Gnai1, Gnb2, Gnai2, Pik3r1, Gnb3, Creb5, Akt1, Mapk9, Mmp2, Acta2
19	mmu04611	Platelet activation	42	0.019702044	*Pik3r2, Adcy7, Mapk3, Rasgrp2, Gucy1b1, P2ry12, Adcy6, Plcb2, Adcy5, Gucy1a1, Tbxa2r, Plcg2, Mylk3, Prkcz, P2ry1, Fyn, Prkci, Pik3cb, Tbxas1, Plcb3, Pik3r3, Myl12b, Adcy1, Itga2b, Arhgef1, Prkg2, Nos3, Itpr3, Plcb1, Gucy1a2, Mylk4, Adcy3, Prkacb, Pik3cg, Gnai1, Itpr1, Vasp, Gnai2, Pik3r1, Pla2g4b, Tln1, Akt1*
20	mmu04713	Circadian entrainment	42	0.000129365	*Calm2, Adcy7, Mapk3, Per3, Gucy1b1, Gng11, Adcy6, Plcb2, Adcy5, Gucy1a1, Adcyap1r1, Gng7, Prkcb, Gng8, Creb1, Plcb3, Cacna1h, Per2, Adcy1, Gnb5, Gria3, Prkg2, Grin2a, Gng2, Itpr3, Plcb1, Gucy1a2, Calm3, Gngt2, Adcy3, Prkacb, Calml4, Gnai1, Gnb2, Grin2d, Ryr2, Itpr1, Rps6ka5, Gnai2, Gria4, Gnb3, Cacna1g*
21	mmu04270	Vascular smooth muscle contraction	41	0.024043548	*Calm2, Adcy7, Mapk3, Pla2g2d, Gucy1b1, Ppp1r14a, Adcy6, Plcb2, Adcy5, Gucy1a1, Mylk3, Myl9, Gna11, Gna12, Prkcb, Plcb3, Adcy1, Mrvi1, Npr2, Arhgef1, Calcrl, Agtr1a, Itpr3, Adra1b, Plcb1, Gucy1a2, Calm3, Ppp1r12c, Ramp2, Mylk4, Adcy3, Prkacb, Calml4, Pla2g12b, Itpr1, Pla2g2e, Pla2g4b, Myh11, Npr1, Adra1a, Acta2*
22	mmu04725	Cholinergic synapse	40	0.008802048	*Pik3r2, Adcy7, Mapk3, Gng11, Adcy6, Plcb2, Adcy5, Kcnq1, Cacna1a, Gna11, Fyn, Gng7, Prkcb, Gng8, Pik3cb, Creb1, Plcb3, Pik3r3, Adcy1, Chrm3, Gnb5, Gng2, Itpr3, Plcb1, Creb3l3, Kras, Gngt2, Adcy3, Prkacb, Pik3cg, Gnai1, Gnb2, Itpr1, Kcnj14, Gnai2, Pik3r1, Gnb3, Creb5, Akt1, Kcnj4*
23	mmu04925	Aldosterone synthesis and secretion	40	0.000477998	*Calm2, Adcy7, Camk1d, Adcy6, Lipe, Star, Plcb2, Kcnk3, Atp1b1, Adcy5, Camk1, Gna11, Scarb1, Prkcb, Atp1a3, Creb1, Plcb3, Cacna1h, Prkd3, Prkd1, Adcy1, Daglb, Dagla, Atp2b2, Agtr1a, Itpr3, Prkd2, Plcb1, Creb3l3, Calm3, Adcy3, Prkacb, Calml4, Itpr1, Nr4a2, Creb5, Atp2b1, Npr1, Camk1g, Cacna1g*
24	mmu04724	Glutamatergic synapse	39	0.015477592	*Adcy7, Mapk3, Gng11, Adcy6, Plcb2, Grik5, Adcy5, Dlg4, Cacna1a, Gng7, Shank2, Homer3, Prkcb, Gng8, Pld2, Plcb3, Adcy1, Gnb5, Gria3, Grin2a, Gng2, Itpr3, Plcb1, Gngt2, Adcy3, Prkacb, Grm8, Gnai1, Slc1a6, Gnb2, Grin2d, Itpr1, Slc17a8, Gnai2, Pla2g4b, Gria4, Gnb3, Grin3a, Grm3*
25	mmu04916	Melanogenesis	37	0.006935061	*Calm2, Adcy7, Fzd7, Mapk3, Fzd2, Kitl, Adcy6, Plcb2, Tcf7l1, Adcy5, Dvl2, Wnt9a, Tcf7l2, Wnt6, Prkcb, Wnt5b, Creb1, Plcb3, Fzd10, Wnt7b, Adcy1, Plcb1, Creb3l3,*

					Calm3, Kras, Wnt2, Adcy3, Dvl3, Wnt2b, Prkacb, Calml4, Gnai1, Wnt10b, Fzd3, Wnt11, Gnai2, Wnt16
26	mmu04928	Parathyroid hormone synthesis, secretion and action	36	0.014101168	*Adcy7, Mapk3, Pthlh, Arrb2, Adcy6, Pth1r, Plcb2, Adcy5, Gna11, Fgfr1, Gna12, Prkcb, Pld2, Creb1, Plcb3, Jund, Adcy1, Lrp6, Arrb1, Arhgef1, Itpr3, Plcb1, Rxra, Creb3l3, Pde4c, Mmp15, Adcy3, Mmp16, Prkacb, Gnai1, Itpr1, Pde4a, Mmp17, Gnai2, Nr4a2, Creb5*
27	mmu04970	Salivary secretion	35	0.000184052	*Adrb3, Calm2, Adcy7, Gucy1b1, Adcy6, Plcb2, Atp1b1, Adcy5, Gucy1a1, Kcnn4, Adrb1, Fxyd2, Prkcb, Atp1a3, Plcb3, Lyz2, Slc4a2, Adcy1, Chrm3, Atp2b2, Prkg2, Itpr3, Adra1b, Plcb1, Gucy1a2, Calm3, Lyz1, Adcy3, Trpv6, Prkacb, Calml4, Itpr1, Slc12a2, Atp2b1, Adra1a*
28	mmu01522	Endocrine resistance	32	0.048455747	*Pik3r2, Adcy7, Mapk3, Cdkn2c, E2f1, Adcy6, Adcy5, Cdkn1b, Bad, Jag2, Pik3cb, Igf1, Esr1, E2f2, Pik3r3, Mmp9, Adcy1, Kras, Adcy3, Gper1, Prkacb, Bik, Ccnd1, Rb1, Jag1, Dll4, Pik3r1, Ncoa3, Notch3, Akt1, Mapk9, Mmp2*
29	mmu04640	Hematopoietic cell lineage	32	0.024043548	*Anpep, Epor, H2-DMa, H2-DMb2, Kitl, Flt3l, H2-DMb1, Cd2, Csf1r, Siglech, H2-Ob, Mme, Itga6, Cd37, Flt3, Itga2b, Il4, Cd36, Il7, H2-Eb1, Cd34, Cd4, Ms4a1, Il11ra1, H2-Aa, H2-Ab1, Cd19, Cr2, Cd55, H2-Oa, Cd22, Cd1d1*
30	mmu05032	Morphine addiction	32	0.015045169	*Adcy7, Arrb2, Gng11, Adcy6, Pde3b, Adcy5, Cacna1a, Gng7, Prkcb, Gng8, Pde8a, Pde10a, Adcy1, Gnb5, Arrb1, Gng2, Pde4c, Gngt2, Adcy3, Grk4, Prkacb, Gnai1, Gnb2, Pde8b, Gabbr1, Pde4a, Gabra3, Gabrr1, Gabra1, Gnai2, Gnb3, Gabrg3*
31	mmu00983	Drug metabolism - other enzymes	32	0.008721473	*Gstt3, Gstt2, Gstt1, Gusb, Cyp2e1, Uck1, Upb1, Tk2, Nme4, Nme7, Gsta3, Dut, Ces1f, Gsta4, Upp2, Mgst2, Tymp, Tk1, Tpmt, Gstm1, Cda, Rrm1, Gsto2, Nat1, Gstm6, Dpyd, Ces1d, Ces2e, Gsta2, Ugt1a6b, Ugt1a6a, Rrm2b*
32	mmu04972	Pancreatic secretion	29	0.038202564	*Adcy7, Pla2g2d, Adcy6, Plcb2, Atp1b1, Adcy5, Slc26a3, Cftr, Kcnq1, Fxyd2, Prkcb, Atp1a3, Rab3d, Plcb3, Slc4a2, Adcy1, Chrm3, Atp2b2, Atp2a3, Itpr3, Plcb1, Adcy3, Pla2g12b, Ryr2, Itpr1, Pla2g2e, Slc12a2, Rab27b, Atp2b1*
33	mmu05217	Basal cell carcinoma	29	0.000590565	*Fzd7, Fzd2, Gli1, Gli2, Tcf7l1, Bmp4, Dvl2, Wnt9a, Axin2, Ptch2, Smo, Tcf7l2, Wnt6, Wnt5b, Apc2, Ddb2, Fzd10, Wnt7b, Hhip, Ptch1, Kif7, Wnt2, Dvl3, Wnt2b, Wnt10b, Fzd3, Wnt11, Polk, Wnt16*

34	mmu00980	Metabolism of xenobiotics by cytochrome P450	27	0.003664137	*Gstt3, Gstt2, Ephx1, Hpgds, Gstt1, Adh1, Cyp2e1, Aldh3b1, Cyp1a1, Adh7, Cyp2s1, Gsta3, Cbr3, Aldh3b2, Cbr1, Gsta4, Mgst2, Gstm1, Gsto2, Dhdh, Gstm6, Cyp2f2, Hsd11b1, Cbr2, Gsta2, Ugt1a6b, Ugt1a6a*
35	mmu04923	Regulation of lipolysis in adipocytes	27	0.000471319	*Pik3r2, Irs3, Adrb3, Adcy7, Aqp7, Npy1r, Plin1, Adcy6, Pde3b, Lipe, Adcy5, Irs1, Adrb1, Pik3cb, Pik3r3, Mgll, Adcy1, Prkg2, Fabp4, Adcy3, Prkacb, Gnai1, Tshb, Gnai2, Pik3r1, Akt1, Npr1*
36	mmu04971	Gastric acid secretion	26	0.039249691	*Calm2, Adcy7, Kcnk2, Adcy6, Plcb2, Atp1b1, Adcy5, Cftr, Mylk3, Kcnq1, Prkcb, Atp1a3, Plcb3, Slc4a2, Adcy1, Chrm3, Itpr3, Plcb1, Calm3, Mylk4, Adcy3, Prkacb, Calml4, Gnai1, Itpr1, Gnai2*
37	mmu04662	B cell receptor signaling pathway	26	0.033624415	*Pik3r2, Mapk3, Nfatc2, Cd81, Rasgrp3, Cd79b, Plcg2, Blnk, Nfatc1, Prkcb, Pik3cb, Pik3r3, Inpp5d, Ptpn6, Cd79a, Vav3, Nfatc3, Vav2, Kras, Rac3, Cd19, Cd72, Cr2, Pik3r1, Akt1, Cd22*
38	mmu04927	Cortisol synthesis and secretion	26	0.008802048	*Adcy7, Kcnk2, Pbx1, Adcy6, Star, Plcb2, Kcnk3, Adcy5, Gna11, Scarb1, Pde8a, Creb1, Plcb3, Cacna1h, Adcy1, Agtr1a, Itpr3, Plcb1, Creb3l3, Adcy3, Prkacb, Pde8b, Itpr1, Nceh1, Creb5, Cacna1g*
39	mmu04924	Renin secretion	25	0.024942879	*Adrb3, Calm2, Gucy1b1, Adcy6, Pde3b, Plcb2, Adcy5, Gucy1a1, Adcyap1r1, Adrb1, Creb1, Plcb3, Prkg2, Agtr1a, Itpr3, Plcb1, Gucy1a2, Calm3, Prkacb, Calml4, Gnai1, Itpr1, Aqp1, Gnai2, Npr1*
40	mmu00982	Drug metabolism - cytochrome P450	24	0.032629792	*Gstt3, Gstt2, Hpgds, Fmo5, Fmo3, Gstt1, Adh1, Cyp2e1, Aldh3b1, Aox3, Fmo2, Adh7, Gsta3, Aldh3b2, Gsta4, Mgst2, Gstm1, Gsto2, Gstm6, Gsta2, Fmo6, Ugt1a6b, Maoa, Ugt1a6a*
41	mmu02010	ABC transporters	21	0.006641821	*Abca8a, Abca6, Abca9, Abca8b, Abcg3, Abcd1, Cftr, Abcg8, Abcc10, Abcg5, Abcc6, Abcd2, Abca4, Abcg1, Abcg4, Abca3, Abcd4, Abcb9, Abca2, Abca5, Abca7*
42	mmu04340	Hedgehog signaling pathway	21	0.005631562	*Arrb2, Gli1, Evc, Gli2, Gas1, Cdon, Kif3a, Ptch2, Smo, Csnk1g2, Boc, Hhip, Ptch1, Arrb1, Kif7, Prkacb, Ccnd1, Evc2, Lrp2, Smurf1, Csnk1g1*
43	mmu03460	Fanconi anemia pathway	20	0.048455747	*Rpa3, Slx1b, Fancg, Poli, Fancb, Brca2, Telo2, Poln, Rmi1, Fancl, Palb2, Mlh1, Rad51, Polh, Rev1, Fancc, Polk, Hes1, Fancm, Fancd2*
44	mmu04672	Intestinal immune	20	0.004007585	*H2-DMa, H2-DMb2, H2-DMb1, Tnfrsf17, Tnfsf13, Itgb7, H2-Ob, Cxcr4, Cxcl12,*

		network for IgA production			*Tgfb1, Il4, Tnfrsf13c, H2-Eb1, Cd28, H2-Aa, H2-Ab1, Tnfrsf13b, Ccr10, H2-Oa, Tnfsf13b*
45	mmu05030	Cocaine addiction	19	0.026830876	*Gpsm1, Adcy5, Dlg4, Bdnf, Creb1, Slc18a2, Grin2a, Creb3l3, Fosb, Prkacb, Gnai1, Cdk5, Grin2d, Gnai2, Creb5, Maoa, Ddc, Grin3a, Grm3*
46	mmu04960	Aldosterone-regulated sodium reabsorption	17	0.009528724	*Slc9a3r2, Pik3r2, Mapk3, Atp1b1, Irs1, Scnn1a, Fxyd2, Prkcb, Pik3cb, Nr3c2, Igf1, Atp1a3, Pik3r3, Scnn1b, Kras, Hsd11b2, Pik3r1*
47	mmu00515	Mannose type O-glycan biosynthesis	11	0.037854303	*Chst10, B4galt2, Pomk, Fktn, B4gat1, Pomgnt2, B3galnt2, Pomt1, Mgat5b, Pomt2, Large2*
48	mmu00531	Glycosaminoglycan degradation	11	0.008146317	*Hyal2, Gusb, Hgsnat, Ids, Sgsh, Hyal1, Hexa, Idua, Naglu, Glb1, Galns*
49	mmu05310	Asthma	10	0.024043548	*H2-DMa, H2-DMb2, H2-DMb1, H2-Ob, Il4, H2-Eb1, Ms4a2, H2-Aa, H2-Ab1, H2-Oa*
