# Supplementary material for: Transcriptomics Changes in the Peritoneum of Mice with Lipopolysaccharide-Induced Peritonitis
Source: Int J Mol Sci. 2021 Nov 30;22(23):13008. doi: 10.3390/ijms222313008 (PMC8657704; doi:10.3390/ijms222313008)
Supplement: Supplementary file 1 [file ijms-22-13008-s001.zip › ijms-1473149-supplementary/Supplementary_14_Table S13. KEGG enrichment pathways of up-regulated DEGs (LPS vs. Control).pdf]

International Journal of *Molecular Sciences*

MDPI

Table S13. KEGG enrichment pathways of up-regulated DEGs (LPS vs. Control)

	Pathway ID	Name	Gene count	PValue	Genes
1	mmu04060	Cytokine-cytokine receptor interaction	110	2.41E-07	*Cxcl1, Cxcl9, Il1rn, Il6, Cxcl2, Ccl2, Ccl5, Ccl7, Ccl22, Cxcl10, Il15ra, Csf3r, Cd40, Il1b, Fas, Csf3, Tnfrsf9, Ccl4, Ccl3, Il4ra, Ngf, Ccl17, Cxcl5, Osmr, Tnfsf9, Cxcl3, Ccr7, Il17a, Il22, Tnf, Il2rg, Il17f, Il19, Ccl12, Il18rap, Il13ra1, Lif, Cxcr2, Il1a, Cxcl16, Il20rb, Ackr3, Acvr1b, Il1r1, Ccl9, Il17ra, Csf2rb, Il1f9, Cxcl14, Il27, Csf2, Tnfsf10, Il10, Bmpr1b, Il1rl1, Tnfrsf12a, Inhbb, Ccl11, Il2ra, Ifng, Csf2rb2, Tnfrsf8, Bmp2, Cx3cl1, Tnfrsf10b, Ifngr1, Il7r, Il3ra, Tnfrsf1b, Il2rb, Tnfrsf1a, Il10ra, Crlf2, Osm, Ccr5, Csf1, Ltbr, Tnfrsf11a, Il13ra2, Tnfsf11, Cntfr, Tnfsf8, Thpo, Tnfrsf4, Il15, Ccl24, Ccr8, Gdf15, Ppbp, Fasl, Il1rl2, Ifnar2, Edar, Ccl19, Gm2564, Il33, Tgfb2, Tnfsf14, Il20ra, Bmp8a, Il6st, Il1r2, Tnfrsf21, Bmpr2, Xcl1, Relt, Ccr1, Eda2r, Tnfrsf18, Ifnar1*
2	mmu05169	Epstein-Barr virus infection	104	4.94E-12	*Il6, Tnfaip3, Isg15, Cdkn1a, Ikbke, Tap1, Nfkbia, Cxcl10, Cd40, Fas, Irf7, Oas1g, H2-Q4, Myc, Stat2, Nfkbib, Myd88, Relb, Oas3, Tbk1, Gadd45a, Irf9, Stat1, Cd44, Nfkbie, Tnf, Nfkb2, H2-Q6, Icam1, Oas1a, Oas2, Stat3, Bcl2l11, Ddx58, Apaf1, Gadd45b, Tap2, H2-Q7, H2-T22, Tlr2, Rela, Runx3, Psmd11, Eif2ak2, Tapbp, Psmd3, H2-K1, H2-M3, Lyn, Oas1b, Sap30, B2m, H2-T23, Chuk, Jak1, Nfkb1, Map2k4, Psmc4, Gadd45g, Ripk1, Casp8, Adrm1, Bid, Traf2, H2-T24, Psmc2, Rac1, Psmd14, Bax, H2-D1, Usp7, Trp53, Psmd4, Psmd8, Psmd7, Map2k3, Psmd2, H2-T-ps, Jak3, Psmd1, Psmc5, Bak1, Mdm2, Map2k7, Ifnar2, Traf6, E2f3, Psmc1, H2-Q2, Jun, Tab2, Ikbkb, -, Ncor2, Entpd3, H2-M9, Psmd6, Psmd12, Ccne1, Psmc3, Cycs, Ifnar1, Calr, H2-T3*
3	mmu05168	Herpes simplex infection	96	4.94E-12	*Il6, Ccl2, Ccl5, Ikbke, Tap1, Socs3, Nfkbia, Il1b, Fas, Irf7, Oas1g, Ifit1, H2-Q4, Stat2, Nfkbib, Myd88, Oas3, Tbk1, Daxx, Irf9, Stat1, Traf1, Tlr3, Tnf, H2-Q6, Oas1a, Ccl12, Oas2, Taf4b, Ddx58, Ifih1, Srsf3, Sp100, Tap2, H2-Q7, Pml, H2-T22, Fos, Tlr2, Rela, Hnrnpk, Srsf5, Eif2ak2, Eif2s1, H2-K1, Arntl, H2-M3, Ifit1bl1, Oas1b, Alyref, Ifng, H2-T23, Chuk, Jak1, Nfkb1, Srpk1, Jak2, Per1, Ifngr1, Srsf7, C1qbp, Casp8, Srsf2, Tnfrsf1a, Taf5l, Srsf6, Traf2, H2-T24, Csnk2a2, H2-D1, Usp7, Trp53, Mcrs1, H2-T-*

					ps, Il15, Srsf1, Eif2ak1, Fasl, Cul1, Ifnar2, Traf6, Eif2ak4, Crebbp, H2-Q2, Jun, Eef1d, Tab2, Ikbkb, Ppp1cc, Tnfsf14, Med8, H2-M9, Csnk2b, Cycs, Ifnar1, H2-T3
4	mmu05166	Human T-cell leukemia virus 1 infection	91	0.019580468	*Il6, Zfp36, Cdkn1a, Nfkbia, Il15ra, Cd40, Slc2a1, Ets2, H2-Q4, Myc, Relb, Stat5a, Fosl1, Tnf, Il2rg, Nfkb2, H2-Q6, Icam1, Vcam1, Atf3, Smad3, Il1r1, Ranbp1, H2-Q7, H2-T22, Fos, Egr1, Rela, Csf2, Atf4, Bcl2l1, Slc25a5, H2-K1, Elk4, H2-M3, Ran, Pcna, Il2ra, H2-T23, Chuk, Jak1, Nfkb1, Mybl2, Map2k4, Trp53inp1, Nfyb, Crtc2, Xbp1, Nras, Crem, Lck, Ets1, Il2rb, Tnfrsf1a, Adcy2, H2-T24, Bax, H2-D1, Ltbr, Trp53, Tspo, Kat5, H2-T-ps, Il15, Jak3, Ranbp3, Bub3, Spi1, Fzd4, Atf1, Pttg1, Anapc7, Itgb2l, Egr2, Pole4, E2f3, Crebbp, H2-Q2, Pdgfrb, Jun, Ikbkb, Tgfb2, H2-M9, Il1r2, Vdac2, Ppp3cc, Canx, Slc25a4, Pold2, Calr, H2-T3*
5	mmu03013	RNA transport	86	4.94E-12	*Seh1l, Eif5, Elac2, Nup93, Eif1a, Nmd3, Eif4a1, Ddx39b, Nup210, Eif3b, Rnps1, Eif4a3, Pop1, Eif2s1, Eif2b2, Eif3d, Ran, Eif4ebp1, Rangap1, Alyref, Eif4g2, Nup98, Nup62, Strap, Xpo5, Nup54, Eif4e2, Prmt5, Rbm8a, Thoc5, Rae1, Gm49336, Magoh, Ncbp1, Eif2s2, Senp2, Sec13, Thoc1, Eif2b3, Eif3c, Nup205, Eif2b1, Eif3g, Srrm1, Eif3e, Thoc3, Nupl2, Nup188, Pym1, Clns1a, Pabpc1, Eif3i, Magohb, Ube2i, Trnt1, Smn1, Eif4e, Eif3a, Pabpc1l, Eif4g3, Eif3j1, Gemin5, Rpp38, Tgs1, Casc3, Eif3j2, Eef1a2, Sap18b, Nup88, Nup210l, Eif1, Pom121, Nup153, Rpp40, Thoc6, Eif2s3y, Eif2s3x, Eif4g1, Nup50, Pnn, Sap18, Eif2b5, Ranbp2, Eif1ax, Rpp30, Kpnb1*
6	mmu05163	Human cytomegalovirus infection	83	0.005404622	*Il6, Ccl2, Cdkn1a, Ccl5, Tap1, Nfkbia, Il1b, Fas, Ccl4, Ccl3, H2-Q4, Myc, Tbk1, Tnf, H2-Q6, Ccl12, Ptgs2, Stat3, Cxcr2, Il1r1, Tap2, H2-Q7, H2-T22, Gna13, Rela, Atf4, Gng12, Map2k1, Tapbp, H2-K1, H2-M3, Tmem173, B2m, Eif4ebp1, H2-T23, Chuk, Egfr, Jak1, Nfkb1, Cx3cl1, Nras, Ripk1, Rock2, Casp8, Bid, Tnfrsf1a, Adcy2, Traf2, Il10ra, H2-T24, Rac2, Gnb4, Rac1, Mb21d1, Ccr5, Bax, H2-D1, Trp53, Ptk2, Creb3l1, H2-T-ps, Pxn, Bak1, Ptger4, Mdm2, Fasl, Mapk1, E2f3, Arhgef12, H2-Q2, Plcb4, Ikbkb, Creb3l4, Rheb, H2-M9, Grb2, Ppp3cc, Ccr1, Mtor, Cycs, Calr, H2-T3, Crkl*
7	mmu03040	Spliceosome	81	2.36E-15	*Hspa8, U2af1, Tcerg1, Hspa2, Srsf3, Hnrnpc, Hnrnpa1, Ddx39b, Hnrnpk, Srsf5, Eif4a3, Snrpd1, Hspa1a, Rbm17, Sf3b3, Smndc1, Alyref, Tra2a, Rbm25, Prpf31, Snrpa1, Srsf10, Sf3b1, Srsf7, Tra2b, Usp39, Hspa1b, U2af2, Rbm8a, Prpf38b, Wbp11, Dhx15, Srsf2, Hnrnpa3, Magoh, Srsf6, Ncbp1, Snu13, Ddx5, Thoc1, Prpf38a, Puf60, Snrpa, Eftud2, Ppie, Ddx23, Sf3a2, Dhx38, Thoc3, Plrg1, Rbm22,*

					Rbmxl1, Srsf1, Magohb, Snrpf, Prpf4, Snrnp40, Dhx8, Xab2, U2surp, Sf3b6, Zmat2, Isy1, Snrnp70, Gm16286, Pcbp1, Snrpb2, Snrpd3, Snrpb, Sart1, Ddx42, Prpf40a, Ddx46, Sf3a3, Hnrnpu, Prpf18, Snrpc, Snrpd2, Snrpe, Lsm4, Cwc15
8	mmu04621	NOD-like receptor signaling pathway	81	2.14E-10	*Cxcl1, Il6, Cxcl2, Ccl2, Tnfaip3, Ccl5, Ikbke, Nfkbia, Il1b, Gbp5, Irf7, Birc3, Oas1g, Stat2, Nfkbib, Myd88, Gbp3, Oas3, Tbk1, Nlrp3, Ifi207, Irf9, Cxcl3, Casp4, Gbp2, Birc2, Nampt, Ifi204, Stat1, Tnf, Oas1a, Ccl12, Gbp7, Oas2, Mefv, Hsp90ab1, Txn1, Rnf31, Camp, Ripk2, Tank, Nod2, Panx1, Rela, Cybb, Bcl2l1, Oas1b, Tmem173, Txnip, Hsp90aa1, Chuk, Jak1, Nod1, Prkcd, Nfkb1, Gsdmd, Casp12, Nek7, Ripk1, Ripk3, Casp8, Traf2, Erbin, Casp1, Casr, Mapk1, Mcu, Ifnar2, Traf6, Brcc3, Atg12, Dhx33, Jun, Tab2, Plcb4, Ikbkb, Aim2, Dnm1l, Vdac2, Txn2, Ifnar1*
9	mmu05167	Kaposi sarcoma-associated herpesvirus infection	81	1.03E-05	*Cxcl1, Il6, Cxcl2, Zfp36, Cdkn1a, Ikbke, Nfkbia, Hif1a, Fas, Irf7, Pik3r5, H2-Q4, Myc, Stat2, Tbk1, Irf9, Cxcl3, Stat1, Tlr3, Rcan1, H2-Q6, Icam1, Hck, Ptgs2, Stat3, H2-Q7, H2-T22, Fos, Rela, Pik3r6, Mapkapk2, Csf2, Gng12, Eif2ak2, Map2k1, H2-K1, Ubb, H2-M3, Lyn, Angpt2, H2-T23, Chuk, Jak1, Nfkb1, Jak2, Map2k4, Ifngr1, Nras, Casp8, Bid, Tnfrsf1a, Traf2, H2-T24, Gnb4, Rac1, Ccr5, Bax, H2-D1, Trp53, H2-T-ps, Ccr8, Atg14, Bak1, Map2k7, Mapk1, Ifnar2, E2f3, Crebbp, H2-Q2, Jun, Ikbkb, H2-M9, Cd86, Becn1, Il6st, Ppp3cc, Ccr1, Mtor, Cycs, Ifnar1, H2-T3*
10	mmu05164	Influenza A	75	6.97E-09	*Il6, Ccl2, Ccl5, Ikbke, Rsad2, Mx2, Socs3, Nfkbia, Cxcl10, Il1b, Fas, Irf7, Oas1g, Stat2, Nfkbib, Myd88, Oas3, Tbk1, Nlrp3, Irf9, Stat1, Tlr3, Tnf, Icam1, Oas1a, Ccl12, Oas2, Hspa8, Ddx58, Il1a, Hspa2, Ifih1, Trim25, Pml, Ddx39b, Rela, Eif2ak2, Map2k1, Hspa1a, Eif2s1, Tnfsf10, Adar, Oas1b, Actb, Ifng, Jak1, Nup98, Nfkb1, Jak2, Tnfrsf10b, Map2k4, Ifngr1, Hspa1b, Rae1, Tnfrsf1a, Actg1, Casp1, Map2k3, Agfg1, Eif2ak1, Map2k7, Fasl, Furin, Mapk1, Ifnar2, Eif2ak4, Kpna1, Crebbp, Ivns1abp, Jun, Il33, Ikbkb, Tlr7, Cycs, Ifnar1*
11	mmu05170	Human immunodeficiency virus 1 infection	72	0.045837658	*Tap1, Nfkbia, Fas, Bst2, H2-Q4, Myd88, Trim30d, Tbk1, Tnf, H2-Q6, Apobec3, Samhd1, Tap2, H2-Q7, H2-T22, Fos, Tlr2, Rela, Gng12, Bcl2l1, Map2k1, Tapbp, H2-K1, H2-M3, Tmem173, B2m, Trim12c, H2-T23, Chuk, Nfkb1, Nras, Tnfrsf1b, Ripk1, Casp8, Bid, Tnfrsf1a, Traf2, H2-T24, Rac2, Gnb4, Rac1, Mb21d1, Ccr5, Bax, H2-D1, Map2k3, Ptk2, H2-T-ps, Pxn, Fbxw11, Bak1, Map2k7, Fasl, Mapk1, Cul1, Traf6, H2-Q2, Jun, Tab2, Ikbkb, Cfl1, H2-M9, Pak2, Ppp3cc, Cdc25c, Cul5, Pak6,*

					Mtor, Cycs, Calr, H2-T3, Crkl
12	mmu05152	Tuberculosis	68	0.000235258	*Sphk1, Il6, Clec4e, Il1b, Irak2, Myd88, Fcgr4, Fcgr1, Cd14, Stat1, Fcgr2b, Tnf, Tcirg1, Cebpb, Lbp, Il1a, Atp6v1h, Camp, Malt1, Apaf1, Ripk2, Cyp27b1, Nod2, Tlr2, Bcl10, Rela, Sphk2, Fcer1g, Nos2, Il10, Vdr, Rab7, Ifng, Cd209a, Jak1, Nfkb1, Hspa9, Jak2, Camk2d, Ifngr1, Nfyb, Hspd1, Casp8, Bid, Tnfrsf1a, Il10ra, Rab5c, Itgam, Bax, Atp6v0c, Tirap, Cd209c, Plk3, Itgb2l, Mapk1, Cd209e, Traf6, Lamp2, Crebbp, Arhgef12, Rfx5, Fcgr3, Tgfb2, Nfyc, Camk2b, Ppp3cc, Rab5a, Cycs*
13	mmu04062	Chemokine signaling pathway	68	0.014837014	*Cxcl1, Cxcl9, Cxcl2, Ccl2, Ccl5, Ccl7, Ccl22, Nfkbia, Cxcl10, Ccl4, Pik3r5, Ccl3, Stat2, Nfkbib, Ccl17, Cxcl5, Cxcl3, Ccr7, Stat1, Hck, Ccl12, Stat3, Fgr, Cxcr2, Cxcl16, Ccl9, Rela, Cxcl14, Pik3r6, Gng12, Map2k1, Lyn, Ccl11, Chuk, Prkcd, Nfkb1, Cx3cl1, Jak2, Nras, Vav1, Itk, Grk5, Rock2, Adcy2, Rac2, Gnb4, Rac1, Ccr5, Cdc42, Grk1, Ptk2, Pxn, Jak3, Ccl24, Ccr8, Grk2, Ppbp, Mapk1, Rap1b, Ccl19, Gm2564, Plcb4, Ikbkb, Grb2, Xcl1, Ccr1, Rap1a, Crkl*
14	mmu05162	Measles	66	6.97E-09	*Il6, Tnfaip3, Ikbke, Mx2, Nfkbia, Il1b, Fas, Irf7, Oas1g, Stat2, Nfkbib, Myd88, Slamf1, Oas3, Tbk1, Irf9, Stat5a, Stat1, Fcgr2b, Il2rg, Oas1a, Oas2, Hspa8, Stat3, Ddx58, Il1a, Cblb, Hspa2, Ifih1, Tlr2, Rela, Eif2ak2, Hspa1a, Eif2s1, Tnfsf10, Adar, Oas1b, Il2ra, Ifng, Cd209a, Chuk, Jak1, Nfkb1, Jak2, Tnfrsf10b, Ifngr1, Hspa1b, Il2rb, Dok1, Csnk2a2, Trp53, Cd209c, Jak3, Msn, Eif2ak1, Fasl, Ifnar2, Cd209e, Traf6, Eif2ak4, Tab2, Tlr7, Csnk2b, Ccne1, Rack1, Ifnar1*
15	mmu04141	Protein processing in endoplasmic reticulum	66	0.000531441	*Ppp1r15a, Hspa8, Hsp90ab1, Hspa2, Atf4, Eif2ak2, Hspa1a, Eif2s1, Sec24a, Nploc4, Ube2d3, Stt3b, Hsp90aa1, Preb, Hspa5, Plaa, Lman1l, Dnaja2, Ube2g2, Sar1a, Hspa1b, Casp12, Xbp1, Dnajb12, Traf2, Sec23b, Rad23b, Sec13, Bax, Dnajb11, Ufd1, Fbxo6, Ero1l, Ckap4, Nfe2l2, Atf6, Selenos, Cryab, Ssr1, Ssr2, Eif2ak1, Syvn1, Bak1, Wfs1, Map2k7, Dnajc5, Cul1, Ube2j2, Eif2ak4, Nsfl1c, Ssr3, Uggt1, Ube2j1, Amfr, Vcp, Rad23a, Hspbp1, Hsph1, Rrbp1, Canx, Mbtps2, Ubqln1, March6, Calr, Herpud1, Hyou1*
16	mmu04217	Necroptosis	65	8.01E-06	*Tnfaip3, Il1b, Fas, Birc3, Stat2, Zbp1, Mlkl, Nlrp3, Irf9, Birc2, Stat5a, Stat1, Tlr3, Tnf, Chmp4b, Stat3, Glul, Hsp90ab1, H2afy, Il1a, Rnf31, Cybb, Pla2g4c, Eif2ak2, Slc25a5, Tnfsf10, Parp3, Pla2g4e, Ifng, Hsp90aa1, Jak1, Jak2, Tnfrsf10b, Camk2d, Ifngr1, Fth1, Ripk1, Ripk3, Pygm, Casp8, Bid, Tnfrsf1a, Traf2, Cflar, Bax, Casp1, Jak3,*

					Chmp2a, Pgam5, Fasl, Pla2g4d, Ppid, Ifnar2, Il33, Chmp1b, Dnm1l, Stat4, Chmp1a, Ppia, Camk2b, Vdac2, Slc25a4, Sqstm1, Cyld, Ifnar1
17	mmu04668	TNF signaling pathway	64	1.47E-10	*Cxcl1, Il6, Cxcl2, Ccl2, Tnfaip3, Junb, Ccl5, Socs3, Nfkbia, Cxcl10, Il1b, Fas, Birc3, Irf1, Mmp3, Ifi47, Map3k8, Mlkl, Bcl3, Cxcl3, Birc2, Traf1, Tnf, Icam1, Vcam1, Ccl12, Cebpb, Ptgs2, Lif, Edn1, Nod2, Fos, Rela, Csf2, Atf4, Map2k1, Mmp14, Chuk, Nfkb1, Cx3cl1, Map2k4, Sele, Tnfrsf1b, Ripk1, Ripk3, Casp8, Casp7, Tnfrsf1a, Traf2, Cflar, Csf1, Dab2ip, Gm5431, Map2k3, Creb3l1, Il15, Pgam5, Map2k7, Mapk1, Jun, Tab2, Ikbkb, Dnm1l, Creb3l4*
18	mmu05202	Transcriptional misregulation in cancer	63	0.016349133	*Il6, Cdkn1a, Runx1, Cd40, Birc3, Mmp3, Myc, Jmjd1c, H3f3b, Bcl2a1b, Gadd45a, Fcgr1, Cd14, Nfkbiz, Bcl2a1d, Gzmb, Traf1, Bcl2a1a, Smad1, Prom1, Cebpb, Rel, Taf15, Etv6, Gadd45b, Pml, Foxo1, Rela, Nupr1, Nr4a3, Csf2, Bcl2l1, Elk4, Nfkb1, Max, Aff1, Dot1l, Gadd45g, Il2rb, Zbtb16, Ewsr1, Igfbp3, Ddx5, Itgam, Bax, Id2, Zbtb17, Trp53, Ptk2, Hpgd, Spi1, Atf1, Bak1, Mdm2, Slc45a3, Fus, Cdk9, Plat, Cd86, Ccnt1, Il1r2, Mitf, Pax5*
19	mmu04210	Apoptosis	61	1.59E-05	*Tuba1c, Nfkbia, Fas, Birc3, Ngf, Bcl2a1b, Gadd45a, Daxx, Birc2, Bcl2a1d, Gzmb, Traf1, Bcl2a1a, Tnf, Bcl2l11, Apaf1, Gadd45b, Fos, Csf2rb, Rela, Atf4, Bcl2l1, Map2k1, Mcl1, Eif2s1, Prf1, Tnfsf10, Parp3, Actb, Csf2rb2, Chuk, Nfkb1, Pmaip1, Tnfrsf10b, Il3ra, Htra2, Casp12, Nras, Gadd45g, Ripk1, Casp8, Casp7, Bid, Tnfrsf1a, Traf2, Cflar, Tuba8, Bax, Tuba4a, Actg1, Dab2ip, Trp53, Ctsl, Bak1, Fasl, Mapk1, Jun, Ikbkb, Tuba1b, Ctsz, Cycs*
20	mmu04145	Phagosome	60	0.009509006	*Tuba1c, Tap1, H2-Q4, Fcgr4, Fcgr1, Tubb6, Cd14, Fcgr2b, H2-Q6, Tcirg1, Stx12, Msr1, Olr1, Atp6v1h, Tap2, H2-Q7, H2-T22, Tlr2, Thbs1, Cybb, Atp6v1b2, Itga5, H2-K1, H2-M3, Actb, Rab7, H2-T23, Cd209a, Dync1li1, Tubb4b, Stx7, Tubb3, H2-T24, Rab5c, Tuba8, Itgam, Rac1, H2-D1, Tuba4a, Actg1, Atp6v0c, Ctsl, Cd209c, H2-T-ps, Sftpa1, Itgb2l, Cd209e, Lamp2, H2-Q2, Fcgr3, H2-M9, Pikfyve, Stx18, Tuba1b, Canx, M6pr, Tfrc, Rab5a, Calr, H2-T3*
21	mmu04218	Cellular senescence	60	0.039700493	*Il6, Cdkn1a, Serpine1, H2-Q4, Myc, Gadd45a, H2-Q6, Smad3, Il1a, Gadd45b, H2-Q7, H2-T22, Foxo1, Rela, Mapkapk2, Zfp36l1, Map2k1, Slc25a5, H2-K1, Cacna1d, H2-M3, Eif4ebp1, H2-T23, E2f4, Nfkb1, Mybl2, Nras, Gadd45g, Ets1, Igfbp3, H2-T24, H2-D1, Trp53, Map2k3, H2-T-ps, Traf3ip2, Fbxw11, Mdm2, Mapk1, Ppid,*

					E2f5, Trpv4, Mcu, E2f3, H2-Q2, Rbl1, Nbn, Tgfb2, Ppp1cc, Rheb, H2-M9, Hipk1, Vdac2, Ppp3cc, Ccne1, Slc25a4, Sqstm1, Mtor, H2-T3, Foxm1
22	mmu04630	JAK-STAT signaling pathway	57	0.003473429	*Il6, Ptpn2, Cdkn1a, Socs3, Il15ra, Csf3r, Csf3, Il4ra, Pim1, Myc, Stat2, Osmr, Irf9, Stat5a, Stat1, Il22, Il2rg, Il19, Stat3, Il13ra1, Lif, Socs1, Il20rb, Csf2rb, Csf2, Bcl2l1, Mcl1, Il10, Il2ra, Ifng, Csf2rb2, Egfr, Jak1, Jak2, Ifngr1, Il7r, Il3ra, Il2rb, Il10ra, Crlf2, Osm, Il13ra2, Cntfr, Thpo, Il15, Jak3, Socs2, Ifnar2, Crebbp, Pdgfrb, Il20ra, Stat4, Il6st, Grb2, Pias1, Mtor, Ifnar1*
23	mmu04380	Osteoclast differentiation	55	0.000335462	*Junb, Socs3, Nfkbia, Il1b, Stat2, Fcgr4, Relb, Fcgr1, Irf9, Fosl1, Stat1, Lcp2, Fcgr2b, Tnf, Nfkb2, Socs1, Il1a, Il1r1, Fos, Pirb, Rela, Lilrb4a, Map2k1, Ifng, Chuk, Jak1, Nfkb1, Ifngr1, Fosl2, Lck, Tnfrsf1a, Traf2, Rac1, Csf1, Sirpb1b, Tnfrsf11a, Tnfsf11, Spi1, Map2k7, Mapk1, Ifnar2, Traf6, Jun, Tab2, Calcr, Ikbkb, Fcgr3, Tgfb2, Grb2, Ppp3cc, Gab2, Mitf, Sqstm1, Cyld, Ifnar1*
24	mmu03008	Ribosome biogenesis in eukaryotes	54	6.21E-14	*Gnl3, Eif6, Nop56, Wdr43, Utp14a, Pwp2, Gtpbp4, Gar1, Tbl3, Fbl, Nhp2, Heatr1, Nmd3, Nat10, Lsg1, Utp4, Wdr75, Nop58, Spata5, Utp15, Gnl2, Pop1, Ran, Nob1, Mphosph10, Tcof1, Rcl1, Rrp7a, Riok1, Mdn1, Dkc1, Wdr36, Riok2, Utp18, Xrn2, Snu13, Fcf1, Csnk2a2, Wdr3, Efl1, Imp4, Nol6, Nvl, Utp6, Sbds, Rpp38, Imp3, Rexo1, Bms1, Rpp40, Csnk2b, Rpp30, Rexo2, Emg1*
25	mmu04064	NF-kappa B signaling pathway	53	2.22E-07	*Cxcl2, Tnfaip3, Nfkbia, Cd40, Il1b, Ccl4, Birc3, Myd88, Relb, Bcl2a1b, Birc2, Cd14, Bcl2a1d, Traf1, Bcl2a1a, Tnf, Nfkb2, Icam1, Vcam1, Card14, Ptgs2, Lbp, Ddx58, Malt1, Trim25, Il1r1, Gadd45b, Bcl10, Rela, Bcl2l1, Lyn, Chuk, Nfkb1, Ripk1, Zap70, Lck, Tnfrsf1a, Traf2, Cflar, Csnk2a2, Ltbr, Tnfrsf11a, Tnfsf11, Tirap, Ly96, Ube2i, Traf6, Ccl19, Gm2564, Tab2, Ikbkb, Tnfsf14, Csnk2b*
26	mmu05160	Hepatitis C	53	7.12E-05	*Cdkn1a, Ikbke, Socs3, Nfkbia, Irf7, Irf1, Oas1g, Ifit1, Stat2, Ldlr, Oas3, Tbk1, Irf9, Stat1, Tlr3, Tnf, Cldn5, Oas1a, Oas2, Stat3, Ddx58, Psme3, Rela, Eif2ak2, Eif2s1, Ifit1bl1, Ppp2r2d, Oas1b, Ppp2ca, Chuk, Egfr, Jak1, Nfkb1, Ppp2r2a, Nras, Ripk1, Tnfrsf1a, Traf2, Ppp2r1a, Trp53, Ppp2cb, Eif3e, Ppara, Eif2ak1, Mapk1, Ifnar2, Traf6, Eif2ak4, Cldn1, Ikbkb, Grb2, Pias1, Ifnar1*
27	mmu05161	Hepatitis B	51	0.008884785	*Il6, Cdkn1a, Ikbke, Nfkbia, Fas, Irf7, Myc, Stat2, Myd88, Tbk1, Stat5a, Stat1, Tlr3, Tnf, Stat3, Ddx58, Ifih1, Apaf1, Fos, Tlr2, Rela, Atf4, Map2k1, Pcna, Ddx3x, Chuk, Jak1, Nfkb1, Map2k4, Casp12, Nras, Casp8, Bax, Trp53, Creb3l1, Tirap, Ywhaz, Fasl,*

					Egr2, Mapk1, E2f3, Crebbp, Jun, Ikbkb, Tgfb2, Creb3l4, Stat4, Grb2, Ccne1, Cycs, Ifnar1
28	mmu04657	IL-17 signaling pathway	49	4.57E-08	*Lcn2, Cxcl1, Il6, Cxcl2, Ccl2, Tnfaip3, S100a8, S100a9, Ikbke, Ccl7, Nfkbia, Cxcl10, Il1b, Mmp13, Csf3, Mmp3, Ccl17, Cxcl5, Tbk1, Cxcl3, Fosl1, Il17a, Tnf, Il17f, Ccl12, Cebpb, Ptgs2, Hsp90ab1, Fos, Il17ra, Rela, Csf2, Ccl11, Ifng, Mapk6, Hsp90aa1, Chuk, Nfkb1, Casp8, Traf2, Usp25, Srsf1, Traf3ip2, Mapk1, Traf6, Jun, Tab2, Ikbkb, Elavl1*
29	mmu04620	Toll-like receptor signaling pathway	45	1.17E-05	*Cxcl9, Il6, Ccl5, Ikbke, Nfkbia, Cxcl10, Cd40, Il1b, Irf7, Ccl4, Ccl3, Myd88, Map3k8, Tbk1, Cd14, Stat1, Tlr3, Tnf, Lbp, Fos, Tlr2, Rela, Map2k1, Cd80, Chuk, Nfkb1, Map2k4, Ripk1, Casp8, Rac1, Map2k3, Tirap, Ly96, Tollip, Map2k7, Mapk1, Ifnar2, Traf6, Jun, Tab2, Ikbkb, Cd86, Spp1, Tlr7, Ifnar1*
30	mmu03015	mRNA surveillance pathway	45	1.59E-05	*Etf1, Smg7, Ddx39b, Rnps1, Eif4a3, Ppp2r2d, Alyref, Ppp2ca, Cpsf2, Ppp2r2a, Cstf1, Rbm8a, Magoh, Ncbp1, Ppp2r1a, Rnmt, Srrm1, Cpsf1, Ppp2cb, Pym1, Rngtt, Pabpc1, Cstf3, Cpsf7, Magohb, Clp1, Pabpc1l, Msi1, Gspt2, Fip1l1, Gspt1, Casc3, Wdr33, Pcf11, Sap18b, Ssu72, Ppp2r3c, Ppp1cc, Pelo, Gm2446, Nudt21, Cstf2, Pnn, Sap18, Sympk*
31	mmu05145	Toxoplasmosis	44	0.007438348	*Nfkbia, Cd40, Irgm1, Pik3r5, Birc3, Igtp, Ldlr, Nfkbib, Myd88, Birc2, Stat1, Tnf, Hspa8, Stat3, Socs1, Hspa2, Tlr2, Rela, Pik3r6, Lamc2, Bcl2l1, Hspa1a, Nos2, Il10, Ifng, Chuk, Jak1, Nfkb1, Jak2, Ifngr1, Hspa1b, Lama5, Casp8, Tnfrsf1a, Il10ra, Ccr5, Map2k3, Ly96, Mapk1, Traf6, Tab2, Ikbkb, Tgfb2, Cycs*
32	mmu04066	HIF-1 signaling pathway	43	0.006442061	*Il6, Cdkn1a, Hif1a, Slc2a1, Serpine1, Timp1, Stat3, Edn1, Pfkfb3, Rela, Cybb, Egln2, Map2k1, Nos2, Eif4ebp1, Angpt2, Pfkl, Ifng, Egfr, Nfkb1, Camk2d, Ifngr1, Eif4e2, Hmox1, Hk2, Egln3, Ltbr, Aldoa, Eno2, Rps6, Rps6-ps4, Ldha, Eif4e, Eno3, Mapk1, Arnt, Hk3, Crebbp, Pgk1, Eno1, Camk2b, Tfrc, Mtor*
33	mmu04625	C-type lectin receptor signaling pathway	43	0.021748277	*Il6, Ikbke, Ccl22, Nfkbia, Clec4e, Il1b, Irf1, Clec4d, Stat2, Ccl17, Relb, Nlrp3, Bcl3, Irf9, Stat1, Tnf, Nfkb2, Ptgs2, Cblb, Malt1, Bcl10, Rela, Mapkapk2, Fcer1g, Il10, Cd209a, Chuk, Prkcd, Nfkb1, Nras, Casp8, Casp1, Cd209c, Plk3, Mdm2, Egr2, Mapk1, Cd209e, Arhgef12, Jun, Ikbkb, Ppp3cc, Cyld*
34	mmu03050	Proteasome	39	6.21E-14	*Psmb8, Psmb10, Psmb9, Psme3, Psme2, Psme1, Psmd11, Psmd3, Psme2b, Ifng, Psmc4, Psma5, Adrm1, Psmc2, Psmd14, Psmb2, Psmd4, Psmd8, Psma2, Psmd7,*

					Psmd2, Psma7, Psmd1, Psma3, Psmf1, Psmb4, Psmc5, Psma6, Psmb7, Pomp, Psma4, Psmc1, Psmb3, Psmd6, Psmd12, Psmb11, Psmb6, Psmb1, Psmc3
35	mmu04612	Antigen processing and presentation	39	1.67E-05	*Tap1, H2-Q4, Tnf, H2-Q6, Hspa8, Hsp90ab1, Psme3, Psme2, Hspa2, Psme1, Tap2, H2-Q7, H2-T22, Hspa1a, Tapbp, H2-K1, H2-M3, B2m, Psme2b, Ifng, H2-T23, Hsp90aa1, Hspa5, Nfyb, Hspa1b, H2-T24, H2-D1, Ctsl, H2-T-ps, Hspa4, H2-Q2, Rfx5, Nfyc, H2-M9, Canx, Klrc1, Calr, H2-T3, Cd8a*
36	mmu05142	Chagas disease (American trypanosomiasis)	39	0.039456274	*Il6, Ccl2, Ccl5, Nfkbia, Il1b, Fas, Ccl3, Serpine1, Myd88, Tnf, Ccl12, Smad3, Fos, Tlr2, Rela, Nos2, Il10, Ppp2r2d, Ppp2ca, Ifng, Chuk, Nfkb1, Map2k4, Ifngr1, Ppp2r2a, Casp8, Tnfrsf1a, Cflar, Ppp2r1a, Ppp2cb, Fasl, Mapk1, Traf6, Jun, Plcb4, Ikbkb, Tgfb2, Gnal, Calr*
37	mmu05132	Salmonella infection	35	0.004219399	*Cxcl1, Il6, Cxcl2, Il1b, Ccl4, Ccl3, Myd88, Cxcl3, Cd14, Lbp, Il1a, Plekhm2, Fos, Rela, Csf2, Flnc, Nos2, Actb, Rab7, Ifng, Dync1li1, Nfkb1, Ifngr1, Arpc3, Rock2, Rac1, Cdc42, Actg1, Casp1, Rhog, Arpc5, Mapk1, Flnb, Jun, Arpc2*
38	mmu03018	RNA degradation	34	0.006442061	*Btg1, Btg2, Pnpt1, Exosc1, Exosc2, Mphosph6, Pfkl, Hspa9, Dis3, Cnot2, Hspd1, Exosc9, Btg3, Zcchc7, Xrn2, Cnot6, Dcp2, Eno2, Cnot7, Tent4a, Exosc10, Exosc3, Pabpc1, Dhx36, Skiv2l, Dcp1a, Pabpc1l, Eno3, Pfkp, Patl1, Ttc37, Eno1, Edc4, Lsm4*
39	mmu05134	Legionellosis	33	1.59E-05	*Cxcl1, Il6, Cxcl2, Nfkbia, Il1b, Myd88, Cxcl3, Cd14, Tnf, Nfkb2, Hspa8, Hspa2, Apaf1, Tlr2, Rela, Arf1, Hspa1a, Nfkb1, Sar1a, Hspa1b, Hspd1, Casp8, Casp7, Itgam, Casp1, Eef1g, Itgb2l, Bnip3, Hsf1, Eef1a2, Vcp, Rab1a, Cycs*
40	mmu05416	Viral myocarditis	32	0.0045993	*Cd40, H2-Q4, H2-Q6, Icam1, H2-Q7, H2-T22, Prf1, H2-K1, H2-M3, Actb, Cd80, H2-T23, Eif4g2, Casp8, Bid, H2-T24, Rac2, Rac1, H2-D1, Abl2, Actg1, H2-T-ps, Sgca, Eif4g3, Itgb2l, H2-Q2, H2-M9, Cd86, Eif4g1, Sgcb, Cycs, H2-T3*
41	mmu05323	Rheumatoid arthritis	31	0.039963171	*Il6, Ccl2, Ccl5, Il1b, Ccl3, Mmp3, Cxcl5, Il17a, Tnf, Icam1, Tcirg1, Ccl12, Il1a, Atp6v1h, Fos, Ctla4, Tlr2, Atp6v1b2, Csf2, Cd80, Ifng, Csf1, Tnfrsf11a, Atp6v0c, Tnfsf11, Ctsl, Il15, Itgb2l, Jun, Tgfb2, Cd86*
42	mmu05140	Leishmaniasis	30	0.006558166	*Nfkbia, Il1b, Marcksl1, Nfkbib, Myd88, Fcgr4, Fcgr1, Stat1, Tnf, Ptgs2, Il1a, Fos, Tlr2, Rela, Cybb, Nos2, Il10, Ifng, Jak1, Nfkb1, Jak2, Ifngr1, Itgam, Itgb2l, Mapk1, Traf6, Jun, Tab2, Fcgr3, Tgfb2*
43	mmu04623	Cytosolic DNA-sensing pathway	29	3.53E-05	*Il6, Ccl5, Ikbke, Nfkbia, Cxcl10, Il1b, Irf7, Ccl4, Nfkbib, Zbp1, Tbk1, Ddx58, Polr3d, Rela, Adar, Tmem173, Chuk, Nfkb1, Ripk1, Ripk3, Mb21d1, Casp1, Polr3h, Polr3a,*

					Polr3c, Polr3e, Il33, Ikbkb, Aim2
44	mmu04622	RIG-I-like receptor signaling pathway	28	0.000742773	*Isg15, Ikbke, Nfkbia, Cxcl10, Irf7, Nfkbib, Dhx58, Tbk1, Tnf, Ddx58, Ifih1, Trim25, Tank, Rela, Azi2, Rnf125, Tmem173, Ddx3x, Chuk, Nfkb1, Ripk1, Casp8, Traf2, Otud5, Traf6, Atg12, Ikbkb, Cyld*
45	mmu05221	Acute myeloid leukemia	28	0.032457495	*Runx1, Pim1, Myc, Bcl2a1b, Fcgr1, Cd14, Stat5a, Bcl2a1d, Bcl2a1a, Stat3, Pml, Rela, Csf2, Map2k1, Ppard, Eif4ebp1, Chuk, Nfkb1, Nras, Zbtb16, Itgam, Kit, Spi1, Pim2, Mapk1, Ikbkb, Grb2, Mtor*
46	mmu00970	Aminoacyl-tRNA biosynthesis	25	0.000392725	*Yars, Tars, Wars, Vars, Farsa, Kars, Gars, Nars, Rars, Iars, Cars, Eprs, Hars, Aars, Lars, Farsb, Yars2, Dars, Ears2, Sars, Mars, Rars2, Mtfmt, Qars, Qrsl1*
47	mmu05332	Graft-versus-host disease	25	0.003473429	*Il6, Il1b, Fas, H2-Q4, Gzmb, Tnf, H2-Q6, Il1a, H2-Q7, H2-T22, Prf1, H2-K1, H2-M3, Cd80, Ifng, H2-T23, H2-T24, H2-D1, H2-T-ps, Fasl, H2-Q2, H2-M9, Cd86, Klrc1, H2-T3*
48	mmu04940	Type I diabetes mellitus	25	0.00782946	*Il1b, Fas, H2-Q4, Gzmb, Tnf, H2-Q6, Il1a, H2-Q7, H2-T22, Prf1, H2-K1, H2-M3, Cd80, Ifng, H2-T23, Hspd1, H2-T24, H2-D1, H2-T-ps, Fasl, H2-Q2, H2-M9, Cd86, Cpe, H2-T3*
49	mmu05144	Malaria	24	0.004219399	*Il6, Ccl2, Cd40, Il1b, Csf3, Myd88, Ackr1, Tnf, Icam1, Vcam1, Ccl12, Selp, Tlr2, Thbs1, Il10, Ifng, Sele, Hba-a1, Gypc, Itgb2l, Hbb-bs, Hba-a2, Tgfb2, Hbb-bt*
50	mmu05330	Allograft rejection	23	0.006558166	*Cd40, Fas, H2-Q4, Gzmb, Tnf, H2-Q6, H2-Q7, H2-T22, Prf1, H2-K1, Il10, H2-M3, Cd80, Ifng, H2-T23, H2-T24, H2-D1, H2-T-ps, Fasl, H2-Q2, H2-M9, Cd86, H2-T3*
51	mmu05320	Autoimmune thyroid disease	23	0.012123369	*Cd40, Fas, H2-Q4, Gzmb, H2-Q6, H2-Q7, H2-T22, Ctla4, Prf1, H2-K1, Il10, H2-M3, Cd80, H2-T23, H2-T24, Tg, H2-D1, H2-T-ps, Fasl, H2-Q2, H2-M9, Cd86, H2-T3*
52	mmu04216	Ferroptosis	20	0.012259552	*Slc39a14, Slc11a2, Sat1, Slc7a11, Cp, Cybb, Acsl4, Slc3a2, Hmox1, Fth1, Map1lc3b, Gclm, Trp53, Slc39a8, Acsl5, Pcbp1, Gss, Pcbp2, Vdac2, Tfrc*
53	mmu05143	African trypanosomiasis	18	0.006558166	*Il6, Il1b, Fas, Ido1, Myd88, Tnf, Icam1, Vcam1, Il10, Ifng, Sele, Thop1, Hba-a1, Fasl, Hbb-bs, Hba-a2, Plcb4, Hbb-bt*
54	mmu04215	Apoptosis - multiple species	16	0.021227592	*Birc3, Birc2, Bcl2l11, Apaf1, Bcl2l1, Pmaip1, Htra2, Casp8, Casp7, Bid, Tnfrsf1a, Bax, Bak1, Birc6, Becn1, Cycs*
