# Supplementary material for: Transcriptomics Changes in the Peritoneum of Mice with Lipopolysaccharide-Induced Peritonitis
Source: Int J Mol Sci. 2021 Nov 30;22(23):13008. doi: 10.3390/ijms222313008 (PMC8657704; doi:10.3390/ijms222313008)
Supplement: Supplementary file 1 [file ijms-22-13008-s001.zip › ijms-1473149-supplementary/Supplementary_15_Table S14. KEGG enrichment pathways of down-regulated DEGs (TAK vs. LPS).pdf]

International Journal of Molecular Sciences

MDPI

Table S14. KEGG enrichment pathways of down-regulated DEGs (TAK vs. LPS)

	Pathway ID	Name	Gene count	PValue	Genes
1	mmu05200	Pathways in cancer	47	0.005475722	*Lpar3, Lrp5, Lama2, Il15ra, Nos2, Il4ra, Il2rb, Ralgds, Ncoa4, Gna13, Txnrd1, Gnb4, Wnt2b, Stat5a, Rassf5, Wnt10b, Il6, Ccnd1, Arnt2, Col4a2, Map2k1, Akt3, Notch2, Notch1, Frat2, Adcy9, Lama5, Col4a3, Frat1, Lamc1, Col4a1, Arhgef11, Casp8, Mtor, Egfr, Apaf1, Fos, Nfkbia, Nfkb1, Rassf1, Raf1, Egln3, Ets1, Fgf23, Stat3, Fn1, Pml*
2	mmu05165	Human papillomavirus infection	31	0.035578422	*Thbs1, Tnc, Lama2, Pard6g, H2-Q2, Itga9, Itgb3, Wnt2b, Wnt10b, Ccnd1, Col4a2, Map2k1, Akt3, Notch2, Notch1, Rbl1, Lama5, Col4a3, Lamc1, Col4a1, Atm, Maml1, Eif2ak2, Casp8, Mtor, Egfr, Nfkb1, Raf1, Col1a1, Tbk1, Fn1*
3	mmu05206	MicroRNAs in cancer	21	0.002679119	*Thbs1, Timp3, Tnc, Itgb3, Bmf, Ccnd1, Slc7a1, Map2k1, Notch2, Notch1, Atm, Cd44, Mtor, Egfr, Nfkb1, Rassf1, Raf1, Abcb1a, Shc1, Stat3, St14*
4	mmu04510	Focal adhesion	21	0.035578422	*Thbs1, Tnc, Lama2, Itga9, Itgb3, Ccnd1, Flnb, Col4a2, Map2k1, Akt3, Lama5, Col4a3, Lamc1, Arhgap35, Col4a1, Egfr, Zyx, Raf1, Col1a1, Shc1, Fn1*
5	mmu04668	TNF signaling pathway	20	0.000357786	*Sele, Icam1, Lif, Il6, Tnfaip3, Socs3, Map2k1, Map3k8, Akt3, Cxcl2, Cx3cl1, Pgam5, Csf1, Casp8, Cxcl1, Cflar, Fos, Nfkbia, Nfkb1, Cxcl10*
6	mmu05164	Influenza A	18	0.017932994	*Icam1, Furin, Il6, Ddx58, Socs3, Map2k1, Hspa1a, Akt3, Ifih1, Eif2ak2, Rsad2, Nfkbia, Nfkb1, Raf1, Cxcl10, Tbk1, Trim25, Pml*
7	mmu05162	Measles	16	0.019406701	*Il2rb, Stat5a, Il6, Ccnd1, Tnfaip3, Ddx58, Hspa1a, Akt3, Cd209a, Slamf1, Ifih1, Eif2ak2, Nfkbia, Nfkb1, Stat3, Tbk1*
8	mmu05224	Breast cancer	16	0.049134121	*Lrp5, Wnt2b, Wnt10b, Ccnd1, Map2k1, Akt3, Notch2, Notch1, Frat2, Frat1, Mtor, Egfr, Fos, Raf1, Fgf23, Shc1*
9	mmu04064	NF-kappa B signaling	15	0.007892187	*Malt1, Icam1, Ccl19, Il1r1, Tnfrsf11a, Tnfaip3, Ddx58, Cxcl2, Lck, Atm, Cflar,*

		pathway			*Nfkbia, Nfkb1, Trim25, Erc1*
10	mmu04926	Relaxin signaling pathway	15	0.048494976	*Nos2, Gnb4, Col4a2, Map2k1, Akt3, Adcy9, Col4a3, Col4a1, Egfr, Fos, Nfkbia, Nfkb1, Raf1, Col1a1, Shc1*
11	mmu04512	ECM-receptor interaction	14	0.005475722	*Thbs1, Tnc, Lama2, Itga9, Itgb3, Sv2c, Col4a2, Lama5, Col4a3, Lamc1, Col4a1, Cd44, Col1a1, Fn1*
12	mmu05146	Amoebiasis	14	0.013639723	*Lama2, Nos2, Il1r1, Serpinb9b, Il6, Col4a2, Lama5, Col4a3, Lamc1, Col4a1, Arg2, Nfkb1, Col1a1, Fn1*
13	mmu04933	AGE-RAGE signaling pathway in diabetic complications	14	0.019406701	*Sele, Icam1, Stat5a, Il6, Ccnd1, Col4a2, Akt3, Col4a3, Col4a1, Nfkb1, Serpine1, Col1a1, Stat3, Fn1*
14	mmu04657	IL-17 signaling pathway	13	0.017805442	*Mapk6, Il6, Tnfaip3, Cxcl2, Casp8, Cxcl1, Traf3ip2, Fos, Nfkbia, Nfkb1, Cxcl10, Tbk1, Il17ra*
15	mmu05222	Small cell lung cancer	13	0.020248587	*Lama2, Nos2, Ccnd1, Col4a2, Akt3, Lama5, Col4a3, Lamc1, Col4a1, Apaf1, Nfkbia, Nfkb1, Fn1*
16	mmu04917	Prolactin signaling pathway	12	0.007892187	*Tnfrsf11a, Stat5a, Ccnd1, Socs3, Map2k1, Akt3, Cish, Fos, Nfkb1, Raf1, Shc1, Stat3*
17	mmu04622	RIG-I-like receptor signaling pathway	10	0.013639723	*Rnf125, Ddx58, Azi2, Ifih1, Casp8, Nfkbia, Nfkb1, Cxcl10, Tbk1, Trim25*
18	mmu05134	Legionellosis	9	0.049134121	*Naip6, Il6, Hspa1a, Cxcl2, Casp8, Cxcl1, Apaf1, Nfkbia, Nfkb1*
19	mmu00532	Glycosaminoglycan biosynthesis - chondroitin sulfate , dermatan sulfate	7	0.005300043	*Csgalnact1, Chst15, Chsy3, Chpf2, Chst11, Xylt1, Chst7*
20	mmu00534	Glycosaminoglycan biosynthesis - heparan sulfate , heparin	7	0.005475722	*Hs6st1, Ndst1, Hs3st3b1, Xylt1, Ndst2, Extl3, Ext1*
