# Supplementary material for: Transcriptomics Changes in the Peritoneum of Mice with Lipopolysaccharide-Induced Peritonitis
Source: Int J Mol Sci. 2021 Nov 30;22(23):13008. doi: 10.3390/ijms222313008 (PMC8657704; doi:10.3390/ijms222313008)
Supplement: Supplementary file 1 [file ijms-22-13008-s001.zip › ijms-1473149-supplementary/Supplementary_16_Table S15. Transcriptional factors predicted from the down-regulated DEGs (LPS vs. Control).pdf]

International Journal of
Molecular Sciences

Table S15. Transcriptional factors predicted from the down-regulated DEGs (LPS vs. Control)

#	Key TF	Description	# of overlapped genes	P value	FDR
1	*Rbpj*	recombination signal binding protein for immunoglobulin kappa J region	3	0.00723	0.118
2	*Isl1*	ISL1 transcription factor, LIM/homeodomain	2	0.0099	0.118
3	*Tbx1*	T-box 1	2	0.013	0.118
4	*Nkx2-5*	NK2 homeobox 5	3	0.0182	0.118
5	*Gata2*	GATA binding protein 2	2	0.0203	0.118
6	*Snai1*	snail family zinc finger 1	3	0.027	0.118
7	*Mybl2*	myeloblastosis oncogene-like 2	2	0.0336	0.118
8	*Nfatc1*	nuclear factor of activated T cells, cytoplasmic, calcineurin dependent 1	2	0.0386	0.118
9	*Gata6*	GATA binding protein 6	2	0.0439	0.118
10	*Mecp2*	methyl CpG binding protein 2	2	0.0495	0.118

Note: data was obtained from TRRUST (version 2) (https://www.grnpedia.org/trrust/)
