# Supplementary material for: Transcriptomics Changes in the Peritoneum of Mice with Lipopolysaccharide-Induced Peritonitis
Source: Int J Mol Sci. 2021 Nov 30;22(23):13008. doi: 10.3390/ijms222313008 (PMC8657704; doi:10.3390/ijms222313008)
Supplement: Supplementary file 1 [file ijms-22-13008-s001.zip › ijms-1473149-supplementary/Supplementary_17_Table S16. Transcriptional factors predicted from the up-regulated DEGs (TAK vs. LPS).pdf]

International Journal of
Molecular Sciences

MDPI

Table S16. Transcriptional factors predicted from the up-regulated DEGs (TAK vs. LPS)

#	Key TF	Description	# of overlapped genes	P value	FDR
1	*Nfkb1*	nuclear factor of kappa light polypeptide gene enhancer in B cells 1, p105	35	2.53e-21	2.48e-19
2	*Stat1*	signal transducer and activator of transcription 1	15	1.9e-16	8.93e-15
3	*Rela*	v-rel reticuloendotheliosis viral oncogene homolog A (avian)	24	2.73e-16	8.93e-15
4	*Jun*	jun proto-oncogene	23	1.86e-14	4.56e-13
5	*Irf1*	interferon regulatory factor 1	12	5.8e-14	1.14e-12
6	*Rel*	reticuloendotheliosis oncogene	10	2.75e-10	4.49e-09
7	*Sp1*	trans-acting transcription factor 1	22	2.22e-07	3.1e-06
8	*Ikbkb*	inhibitor of kappaB kinase beta	5	3.93e-06	4.81e-05
9	*Trp53*	transformation related protein 53	15	5.85e-06	6.37e-05
10	*Irf8*	interferon regulatory factor 8	6	9.17e-06	8.98e-05
11	*Hdac2*	histone deacetylase 2	4	4.78e-05	0.000382
12	*Ets1*	E26 avian leukemia oncogene 1, 5' domain	8	5.01e-05	0.000382
13	*Nr3c1*	nuclear receptor subfamily 3, group C, member 1	5	5.06e-05	0.000382
14	*Nfatc2*	nuclear factor of activated T cells, cytoplasmic, calcineurin dependent 2	5	8.54e-05	0.000597
15	*Foxo1*	forkhead box O1	7	0.000106	0.000671
16	*Dnmt1*	DNA methyltransferase (cytosine-5) 1	3	0.00011	0.000671
17	*Egr1*	early growth response 1	8	0.00019	0.0011
18	*Bcl3*	B cell leukemia/lymphoma 3	4	0.000284	0.00155
19	*Cebpb*	CCAAT/enhancer binding protein (C/EBP), beta	6	0.000332	0.00171
20	*Hdac1*	histone deacetylase 1	5	0.000362	0.00177
21	*Ep300*	E1A binding protein p300	7	0.000385	0.00178
22	*Stat3*	signal transducer and activator of transcription 3	8	0.000399	0.00178

23	*Nr4a1*	nuclear receptor subfamily 4, group A, member 1	3	0.00121	0.00515
24	*Fos*	FBJ osteosarcoma oncogene	6	0.00158	0.00572
25	*Id1*	inhibitor of DNA binding 1	3	0.00163	0.00572
26	*Irf4*	interferon regulatory factor 4	3	0.00163	0.00572
27	*Klf5*	Kruppel-like factor 5	3	0.00163	0.00572
28	*Parp1*	poly (ADP-ribose) polymerase family, member 1	3	0.00163	0.00572
29	*Stat5a*	signal transducer and activator of transcription 5A	4	0.0022	0.00744
30	*Sp3*	trans-acting transcription factor 3	7	0.00266	0.0087
31	*Pax2*	paired box 2	3	0.00343	0.0108
32	*Ppara*	peroxisome proliferator activated receptor alpha	5	0.00363	0.0111
33	*Daxx*	Fas death domain-associated protein	2	0.00483	0.0144
34	*Ikzf1*	IKAROS family zinc finger 1	3	0.0051	0.0146
35	*Pparg*	peroxisome proliferator activated receptor gamma	5	0.00521	0.0146
36	*Myb*	myeloblastosis oncogene	3	0.00609	0.0157
37	*Nr1h3*	nuclear receptor subfamily 1, group H, member 3	3	0.00609	0.0157
38	*Pou2f2*	POU domain, class 2, transcription factor 2	3	0.00609	0.0157
39	*Mtf1*	metal response element binding transcription factor 1	2	0.00714	0.0172
40	*Tcf21*	transcription factor 21	2	0.00714	0.0172
41	*Bcl6*	B cell leukemia/lymphoma 6	3	0.00719	0.0172
42	*Klf4*	Kruppel-like factor 4 (gut)	3	0.0084	0.0187
43	*Nr0b2*	nuclear receptor subfamily 0, group B, member 2	3	0.0084	0.0187
44	*Tcf4*	transcription factor 4	3	0.0084	0.0187
45	*Pou2f1*	POU domain, class 2, transcription factor 1	3	0.00972	0.0201
46	*Rb1*	retinoblastoma 1	3	0.00972	0.0201
47	*Ctcf*	CCCTC-binding factor	2	0.00985	0.0201
48	*Stat4*	signal transducer and activator of transcription 4	2	0.00985	0.0201
49	*Hif1a*	hypoxia inducible factor 1, alpha subunit	3	0.0112	0.0223
50	*Ahr*	aryl-hydrocarbon receptor	3	0.0127	0.0231
51	*Gata4*	GATA binding protein 4	3	0.0127	0.0231

52	*Apex1*	apurinic/apyrimidinic endonuclease 1	2	0.0129	0.0231
53	*Dnmt3a*	DNA methyltransferase 3A	2	0.0129	0.0231
54	*Foxg1*	forkhead box G1	2	0.0129	0.0231
55	*Runx3*	runt related transcription factor 3	2	0.0129	0.0231
56	*Crebbp*	CREB binding protein	4	0.0133	0.0233
57	*Cebpa*	CCAAT/enhancer binding protein (C/EBP), alpha	3	0.0161	0.0263
58	*Foxo3*	forkhead box O3	2	0.0164	0.0263
59	*Gfi1b*	growth factor independent 1B	2	0.0164	0.0263
60	*Ncoa3*	nuclear receptor coactivator 3	2	0.0164	0.0263
61	*Tbx2*	T-box 2	2	0.0164	0.0263
62	*Foxa1*	forkhead box A1	2	0.0202	0.0314
63	*Nfkb2*	nuclear factor of kappa light polypeptide gene enhancer in B cells 2, p49/p100	2	0.0202	0.0314
64	*Myc*	myelocytomatosis oncogene	4	0.0242	0.0361
65	*Ncoa1*	nuclear receptor coactivator 1	2	0.0243	0.0361
66	*Nfic*	nuclear factor I/C	2	0.0243	0.0361
67	*Myod1*	myogenic differentiation 1	4	0.0259	0.0378
68	*Esr1*	estrogen receptor 1 (alpha)	3	0.0268	0.0387
69	*Foxp3*	forkhead box P3	2	0.0288	0.0408
70	*Creb1*	cAMP responsive element binding protein 1	3	0.032	0.0447
71	*Epas1*	endothelial PAS domain protein 1	2	0.0335	0.0449
72	*Fli1*	Friend leukemia integration 1	2	0.0335	0.0449
73	*Mybl2*	myeloblastosis oncogene-like 2	2	0.0335	0.0449
74	*Lef1*	lymphoid enhancer binding factor 1	3	0.0347	0.0459
75	*Etv4*	ets variant 4	2	0.0438	0.0564
76	*Gata6*	GATA binding protein 6	2	0.0438	0.0564
77	*Atf2*	activating transcription factor 2	2	0.0493	0.0627

Note: data was retrieved from TRRUST (version 2) (https://www.grnpedia.org/trrust/)
