# Supplementary material for: Transcriptomics Changes in the Peritoneum of Mice with Lipopolysaccharide-Induced Peritonitis
Source: Int J Mol Sci. 2021 Nov 30;22(23):13008. doi: 10.3390/ijms222313008 (PMC8657704; doi:10.3390/ijms222313008)
Supplement: Supplementary file 1 [file ijms-22-13008-s001.zip › ijms-1473149-supplementary/Supplementary_18_Table S17. Hub Genes Information.pdf]

International Journal of
Molecular Sciences

MDPI

Table S17. Hub Genes Information

Gene name<br>Ensembl ID	Species<br>gene type	Location<br>Length	Expression Changes (TAK 242 vs. LPS)	Function	Refs [Stelzer, et al. The GeneCards Suite: From Gene Data Mining to Disease Genome Sequence Analyses *Curr Protoc Bioinformatics* 2016]
Sele<br>(Selectin E)<br>(ENSEMBL: ENSG00000007908)	Mus musculus<br>Protein coding	Chr 1<br>(2,912 bp)	Down-regulated	Is involved in the modulation of inflammation such as blood leukocytes accumulation, cell adhesion, and endothelium adhesion. It is anticipated in the disorders of African Tick-Bite Fever, atherosclerosis, and Rheumatoid Vasculitis.	[Pastwińska, et al. The Art of Mast Cell Adhesion *Cells* **2020**]
9930111J21Rik2<br>(RIKEN cDNA 9930111J21 gene 2)<br>(Ensembl:ENSMUSG00000069892)	Mus musculus<br>Protein coding	Chr 11<br>(2,403 bp)	Down-regulated	None available.	None
Gzmb<br>(granzyme B)<br>(Ensembl:ENSMUSG00000015437)	Mus musculus<br>Protein coding	Chr 14<br>(1,528 bp)	Down-regulated	Involved in wound healing and chronic inflammation and anticipated in the pathology of diseases such as	[Zeglinski and Granville. Granzymes in cardiovascular

				Aggressive Nk-Cell Leukemia and Peripheral T-Cell Lymphoma	injury and disease ***Cellular Signalling*** **2020**]
Lpar3 (lysophosphatidic acid receptor 3) (Ensembl:ENSMUSG00000036832)	Mus musculus Protein coding	Chr 3 (2,494 bp)	Down-regulated	Acts as a receptor for lysophosphatidic acid and regulates calcium mobilization. It is involved in the GPCR signaling and peptide ligand-binding receptor pathway. Besides, it is associated with the Tracheal cancer and Trachea Adenoid Cystic Carcinoma	[Lin, et al. A Novel Function of the Lysophosphatidic Acid Receptor 3 (LPAR3) Gene in Zebrafish on Modulating Anxiety, Circadian Rhythm Locomotor Activity, and Short-Term Memory ***International Journal of Molecular Sciences*** **2020**]
Card10 (caspase recruitment domain family, member 10) (Ensembl:ENSMUSG00000033170)	Mus musculus Protein coding	Chr 15 (4,748 bp)	Up-regulated	Involved in the apoptosis signaling pathway and is associated with diseases such as Primary Open Angle, Open-Angle Glaucoma, and Glaucoma.	[Zhang and Lin. CARMA3: Scaffold Protein Involved in NF-κB Signaling ***Front Immunol*** **2019**]

Plekhh3 (pleckstrin homology domain containing, family H (with MyTH4 domain) member 3) (Ensembl:ENSMUSG00000035172)	Mus musculus Protein coding	Chr 11 (3,007 bp)	Up-regulated	Involved in endothelial tight junction.	[Chen, et al. Genome-wide gene expression array identifies novel genes related to disease severity and excessive daytime sleepiness in patients with obstructive sleep apnea ***PLoS ONE*** **2017**]
Higd1b (HIG1 domain family, member 1B) (Ensembl:ENSMUSG00000020928)	Mus musculus Protein coding	Chr 11 (735 bp)	Up-regulated	Anticipated in the progression of pituitary adenomas and tumorigenesis.	[Zhou, et al. Identification of Key Genes With Differential Correlations in Lung Adenocarcinoma ***Front Cell Dev Biol*** **2021**]
Gm12689 (predicted gene 12689) (Ensembl: none)	Mus musculus Protein coding	Chr 4 (792 bp)	Up-regulated	None available.	None
Plxdc1 (plexin domain containing 1) (Ensembl:ENSMUSG00000017417)	Mus musculus Protein coding	Chr 11 (2,922 bp)	Up-regulated	Involved in endothelial cell capillary morphogenesis and diseases such as Osteogenic Sarcoma and Mulchandani-Bhoj-Conlin Syndrome.	[van Beijnum, et al. Tumor endothelium is characterized by

					a matrix remodeling signature ***Front Biosci (Schol Ed)*** **2009**]
Pkn3 (protein kinase N3) (Ensembl:ENSMUSG00000026785)	Mus musculus Protein coding	Chr 2 (2,999 bp)	Up-regulated	Contributes to disorders such as Pachyonychia Congenita 1, malignant prostate cancer, and Gastrointestinal Neuroendocrine Tumor.	[Uehara, et al. Regulation of osteoclast function via Rho-Pkn3-c-Src pathways ***J Oral Biosci*** **2019**]
