# Supplementary material for: Transcriptomics Changes in the Peritoneum of Mice with Lipopolysaccharide-Induced Peritonitis
Source: Int J Mol Sci. 2021 Nov 30;22(23):13008. doi: 10.3390/ijms222313008 (PMC8657704; doi:10.3390/ijms222313008)
Supplement: Supplementary file 1 [file ijms-22-13008-s001.zip › ijms-1473149-supplementary/Supplementary_19_Table S18. GO Analysis of Hub Genes.pdf]

International Journal of
Molecular Sciences

MDPI

Table S18. GO Analysis of Hub Genes

**Gene**	**GO analysis** [Smith, et al. The mouse Gene Expression Database (GXD): 2019 update ***Nucleic Acids Research* 2019;** Bult, et al. Mouse Genome Database (MGD) 2019 ***Nucleic Acids Research* 2019**]
Sele	MF: carbohydrate derivative binding; signaling receptor activity
	BP: cellular component organization; establishment of localization; immune system process; response to stimulus
	CC: cytoskeleton; extracellular region; non-membrane-bounded organelle; plasma membrane
9930111J21Rik2	MF: none
	BP: none
	CC: none
Gzmb	MF: hydrolase
	BP: cell death; immune system process; protein metabolic process; response to stimulus; signaling
	CC: extracellular region; vacuole
Lpar3	MF: lipid binding; signaling receptor activity
	BP: cell differentiation; cellular component organization; establishment of localization; homeostatic process; response to stimulus; signaling; system development
	CC: cell projection; plasma membrane
Card10	MF: none
	BP: cell death; protein metabolic process; response to stimulus; signaling; system development

	CC: none
Plekhh3	MF: none
	BP: response to stimulus; signaling; system development
	CC: on-membrane-bounded organelle
Higd1b	MF: none
	BP: cellular component organization
	CC: mitochondrion
Gm12689	MF: none
	BP: none
	CC: none
Plxdc1	MF: none
	BP: system development
	CC: cell projection; plasma membrane
Pkn3	MF: carbohydrate derivative binding; transferase
	BP: protein metabolic process; response to stimulus; signaling
	CC: nucleus

Note: data were obtained from the Mouse Genome Database (MGD) at the Mouse Genome Informatics website (URL: http://www.informatics.jax.org) [Smith, et al. The mouse Gene Expression Database (GXD): 2019 update ***Nucleic Acids Research* 2019;** Bult, et al. Mouse Genome Database (MGD) 2019 ***Nucleic Acids Research* 2019**] [date of retrieving data: August 23, 2021]
