# Supplementary material for: Transcriptomics Changes in the Peritoneum of Mice with Lipopolysaccharide-Induced Peritonitis
Source: Int J Mol Sci. 2021 Nov 30;22(23):13008. doi: 10.3390/ijms222313008 (PMC8657704; doi:10.3390/ijms222313008)
Supplement: Supplementary file 1 [file ijms-22-13008-s001.zip › ijms-1473149-supplementary/Supplementary_2_Table S1. Details of DEGs-protein_coding genes (LPS vs. Ctrl).pdf]

International Journal of
Molecular Sciences

MDPI

Table S1. Details of DEGs-protein_coding genes (LPS vs. Ctrl)

Gene_symbol	log2FoldChange	Padj	Gene_chrosome	Change
Diras2	-7.227158938	8.68E-15	13	Down
Slc26a3	-6.817309891	2.38E-09	12	Down
Ssu2	-6.521561652	1.03E-10	6	Down
Mup20	-6.490688125	0.000800266	4	Down
Lrrc31	-6.455008731	1.26E-06	3	Down
Cdk15	-6.409764656	1.29E-10	1	Down
Abcg8	-6.403276061	6.38E-07	17	Down
Pck1	-6.376271511	1.03E-78	2	Down
Nat8f7	-6.225040769	1.99E-09	6	Down
Necab3	-6.190565407	3.73E-11	2	Down
2810459M11Rik	-6.051087061	2.32E-08	1	Down
Slc16a5	-6.010049261	2.04E-08	11	Down
Aass	-5.787626955	1.09E-08	6	Down
Ccdc27	-5.741419833	1.14E-09	4	Down
Btbd17	-5.682975397	1.73E-08	11	Down
Rnf112	-5.636118987	3.21E-16	11	Down
Skint3	-5.633149386	1.10E-11	4	Down
Hcar1	-5.533768487	9.97E-41	5	Down
Ptchd3	-5.531312005	2.21E-05	11	Down
Foxq1	-5.446658562	2.51E-06	13	Down
Odf3b	-5.432574747	3.61E-07	15	Down
Sprn	-5.404707949	9.26E-09	7	Down
Grb7	-5.37645893	4.95E-06	11	Down
Abcg5	-5.369287498	3.52E-06	17	Down
Sez6	-5.228425899	0.000163561	11	Down
Sertad4	-5.227677872	2.33E-38	1	Down
Gpr165	-5.206991954	3.31E-06	X	Down
Fam198a	-5.185852842	1.92E-23	9	Down
Kcnb2	-5.174676088	0.001095007	1	Down
Abcc6	-5.116486167	3.20E-05	7	Down
Il1a	8.782095807	5.25E-20	2	Up
Mt2	8.660370182	3.77E-180	8	Up
Slamf1	8.634856178	2.55E-50	1	Up
Saa1	8.437687757	2.07E-240	7	Up
Stfa2	8.313215192	9.68E-14	16	Up
Slfn4	8.275767349	2.38E-210	11	Up
Prokr2	8.247548334	4.00E-14	2	Up
Lrfn5	8.114025053	3.60E-08	12	Up
Ccl5	8.084876655	4.78E-103	11	Up
Cxcl9	7.984207178	2.08E-181	5	Up
Sh2d5	7.91000168	4.60E-72	4	Up
Adamts4	7.874385672	5.87E-62	1	Up
Rnd1	7.696790648	2.54E-133	15	Up

Gm15056	7.695134836	1.20E-16	8	Up
Ccl19	7.660939596	0.007795487	4	Up
Ptx3	7.540516519	5.74E-212	3	Up
Gm5483	7.484771148	1.75E-16	16	Up
Clec4e	7.473971526	2.01E-80	6	Up
AA467197	7.421360496	1.07E-138	2	Up
Trem1	7.420841235	1.02E-14	17	Up
Olfr56	7.408139809	3.97E-61	11	Up
Gzmb	7.400785993	7.11E-33	14	Up
Ido1	7.37401006	4.05E-54	8	Up
Rsad2	7.336874961	4.62E-102	12	Up
4930438A08Rik	7.304215903	3.28E-14	11	Up
Dnmt3c	7.30317473	3.94E-11	2	Up
Il1f9	7.272300908	4.04E-14	2	Up
Ccl2	7.264600645	1.20E-140	11	Up
Stfa3	7.261733834	1.18E-16	16	Up
Serpina3i	7.244338562	8.60E-59	12	Up

Note: down-regulated and up-regulated 30 protein-coding genes were demonstrated.
