# Supplementary material for: Transcriptomics Changes in the Peritoneum of Mice with Lipopolysaccharide-Induced Peritonitis
Source: Int J Mol Sci. 2021 Nov 30;22(23):13008. doi: 10.3390/ijms222313008 (PMC8657704; doi:10.3390/ijms222313008)
Supplement: Supplementary file 1 [file ijms-22-13008-s001.zip › ijms-1473149-supplementary/Supplementary_20_Table S19. Protein modeling templates.pdf]

International Journal of
Molecular Sciences

MDPI

Table S19. Protein modeling templates

Proteins	Species	Length (a.a)	Templates (query cover, identify)
SELE	Mus musculus	619	4C16_A (1.93 Å) (85%, 76.16%)<br>6V06_A (2.40 Å) (46%, 29.60%)<br>1C1Z_A (2.87 Å) (46%, 29.60%)
9930111J21RIK2	Mus musculus	467	7C3K_A (2.60 Å) (85%, 48.66%)<br>1TQD_A (2.30 Å) (85%, 44.63%)<br>1TQ6_A (2.70 Å) (85%, 44.63%)
GZMB	Mus musculus	247	1FI8_A (2.20 Å) (91%, 80.70%)<br>1IAU_A (2.00 Å) (91%, 70.22%)<br>3G01_A (2.50 Å) (91%, 68.14%)
LPAR3	Mus musculus	354	4Z36_A (2.90 Å) (84%, 55.56%)<br>7AUE_R (2.97 Å) (75%, 29.02%)<br>6K41_R (2.90 Å) (76%, 28.23%)
CARD10	Mus musculus	1070	*De novo* by Rosetta
PLEKHH3	Mus musculus	796	*De novo* by Rosetta
HIGD1B	Mus musculus	98	*De novo* by Rosetta
GM12689	Mus musculus	117	*De novo* by Rosetta
PLXDC1	Mus musculus	507	*De novo* by Rosetta
PKN3	Mus musculus	878	4OTD_A (2.00 Å) (37%, 65.17%)<br>4CRS_A (2.75 Å) (37%, 63.33%)<br>1O6L_A (1.60 Å) (37%, 44.64%)
