# Supplementary material for: Transcriptomics Changes in the Peritoneum of Mice with Lipopolysaccharide-Induced Peritonitis
Source: Int J Mol Sci. 2021 Nov 30;22(23):13008. doi: 10.3390/ijms222313008 (PMC8657704; doi:10.3390/ijms222313008)
Supplement: Supplementary file 1 [file ijms-22-13008-s001.zip › ijms-1473149-supplementary/Supplementary_21_Table S20. Ramachandran plot of hub proteins.pdf]

International Journal of
Molecular Sciences

MDPI

Table S20. Ramachandran plot of hub proteins

Proteins	Residues in most favored regions	Residues in additional allowed regions	Residues in generously allowed regions	Residues in disallowed regions
SELE	378/475 (79.6%)	73/475 (15.4%)	15/475 (3.2%)	9/475 (1.9%)
9930111J21RIK2	362/401 (90.3%)	31/401 (7.7%)	4/401 (1.0%)	4/401 (1.0%)
GZMB	167/184 (90.8%)	14/184 (7.6%)	3/184 (1.6%)	0/184 (0.0%)
LPAR3	287/317 (90.5%)	26/317 (8.2%)	4/317 (1.3%)	0/317 (0.0%)
CARD10	313/387 (80.9%)	62/387 (16.0%)	7/387 (1.8%)	5/387 (1.3%)
PLEKHH3	551/611 (90.2%%)	50/611 (8.2%)	4/611 (0.7%)	6/611 (1.0%)
HIGD1B	84/85 (98.8%)	1/85 (1.2%)	0/85 (0.0%)	0/85 (0.0%)
GM12689	70/89 (78.7%)	12/89 (13.5%)	4/89 (4.5%)	3/89 (3.4%)
PLXDC1	359/415 (86.5%)	50/415 (12.0%)	2/415 (0.5%)	4/415 (1.0%)
PKN3	428/555 (77.1%)	89/555 (16.0%)	24/555 (4.3%)	14/555 (2.5%)

Note: random coil parts of the N-terminal/C-terminal sequence of the hub proteins were deleted for molecular dynamics simulation.
