# Supplementary material for: Transcriptomics Changes in the Peritoneum of Mice with Lipopolysaccharide-Induced Peritonitis
Source: Int J Mol Sci. 2021 Nov 30;22(23):13008. doi: 10.3390/ijms222313008 (PMC8657704; doi:10.3390/ijms222313008)
Supplement: Supplementary file 1 [file ijms-22-13008-s001.zip › ijms-1473149-supplementary/Supplementary_22_Table S21. Primers for qPCR.pdf]

International Journal of
Molecular Sciences

MDPI

Table S21. Primers for qPCR

Genes		Sequences (5' to 3')	Product (bp)
GAPDH	Forward	GGCAAATTCAACGGCACAGTCAAG	81
	Reverse	TCGCTCCTGGAAGATGGTGATGG	
Sele	Forward	TTGCTCTACTTGTTGATG	143
	Reverse	CATTTGTGTTCCTGATTG	
9930111J21Rik2	Forward	ATTGACACGGTTGCGAGCGATG	125
	Reverse	GGAAACACTTTGCCCACGGACTC	
Gzmb	Forward	AGCATCTCTATTTACAGCGGGAACG	144
	Reverse	GGAGCACCATCCGATGACAAGC	
Lpar3	Forward	GCTCCCATTTACAGTAGGAGTT	101
	Reverse	ACATACATGTAGATGCGTACGT	
Card10	Forward	CGAACGCTGCTGAAGGACTGTG	96
	Reverse	GATTGCCTGGTCCCGCTCTTTC	
Plekhh3	Forward	GACTAAGCCTGCCAAGAG	79
	Reverse	TGGTTGCCTCAAATCTCAG	
Higd1b	Forward	TGTCCAAGAAGTTCCTAAG	88
	Reverse	ATCCTGTATGCTGCTATC	
Gm12689	Forward	GCACAGACAATTACAATG	114
	Reverse	CCTCTAAGTACAGTGATTC	
Plxdc1	Forward	TTAATGGCGACTCCACTAC	76
	Reverse	GGATTCAACTTGGTGTCATC	
Pkn3	Forward	AGGCGTTGAAGAAGCAGGAAGTG	120
	Reverse	AGGCAAGGAGAGAGAGCAGGAAG	
