# Supplementary material for: Transcriptomics Changes in the Peritoneum of Mice with Lipopolysaccharide-Induced Peritonitis
Source: Int J Mol Sci. 2021 Nov 30;22(23):13008. doi: 10.3390/ijms222313008 (PMC8657704; doi:10.3390/ijms222313008)
Supplement: Supplementary file 1 [file ijms-22-13008-s001.zip › ijms-1473149-supplementary/Supplementary_23_Table S22 RMSD-RMSF-Rg.pdf]

International Journal of
Molecular Sciences

MDPI

Table S23. Mean value of RMSD, RMSF, and Rg

Protein	RMSD	RMSF	Rg
SELE	2.315	1.298	3.861
9930111J21RIK2	0.9725	0.3550	0.9725
GZMB	0.2803	0.1658	1.707
LPAR3	0.9497	0.3541	2.425
CARD10	1.823	0.8103	2.937
PLEKHH3	1.534	0.6020	3.253
HIGD1B	0.9055	0.3621	2.070
GM12689	0.6669	0.3518	1.474
PLXDC1	2.207	0.6737	2.580
PKN3	1.035	0.3919	2.791
