# Supplementary material for: Transcriptomics Changes in the Peritoneum of Mice with Lipopolysaccharide-Induced Peritonitis
Source: Int J Mol Sci. 2021 Nov 30;22(23):13008. doi: 10.3390/ijms222313008 (PMC8657704; doi:10.3390/ijms222313008)
Supplement: Supplementary file 1 [file ijms-22-13008-s001.zip › ijms-1473149-supplementary/Supplementary_3_Table S2. Details of DEGs-protein_coding genes (TAK vs. LPS).pdf]

International Journal of
Molecular Sciences

MDPI

Table S2. Details of DEGs-protein_coding genes (TAK vs. LPS)

Gene_symbol	log2FoldChange	Padj	Gene_chrosome	Change
Sele	-2.480305674	0.049100611	1	Down
9930111J21Rik2	-2.442979149	0.049100611	11	Down
Gzmb	-2.324252647	0.006178091	14	Down
Lpar3	-2.014707545	0.047550023	3	Down
Nr4a3	-1.544222339	0.049100611	4	Down
St6gal1	-1.453749932	0.049100611	16	Down
Pkn3	3.281765542	0.032118609	2	Up
Plxdc1	3.039222087	0.040508588	11	Up
Gm12689	3.019761304	0.049100611	4	Up
Higd1b	2.212102654	0.041390252	11	Up
Plekhh3	2.173246157	0.047550023	11	Up
Card10	2.081192293	0.049100611	15	Up
Cbx6	1.949752092	0.004062808	15	Up
Gstt3	1.87286013	0.049100611	10	Up
mt-Nd3	1.868936208	0.049100611	MT	Up
Rbm12b1	1.362563897	0.004157553	4	Up
Ddit3	1.060271348	0.049100611	10	Up

Note: down-regulated and up-regulated protein-coding genes were demonstrated.
