# Supplementary material for: Transcriptomics Changes in the Peritoneum of Mice with Lipopolysaccharide-Induced Peritonitis
Source: Int J Mol Sci. 2021 Nov 30;22(23):13008. doi: 10.3390/ijms222313008 (PMC8657704; doi:10.3390/ijms222313008)
Supplement: Supplementary file 1 [file ijms-22-13008-s001.zip › ijms-1473149-supplementary/Supplementary_4_Table S3. Details of DEGs-promoter_lncRNA genes (LPS vs. Ctrl).pdf]

International Journal of
Molecular Sciences

MDPI

Table S3. Details of DEGs-promoter_lncRNA genes (LPS vs. Ctrl)

Gene_symbol	log2FoldChange	Padj	Gene_chrosome	Change
Gm10614	-3.65283242	0.021667045	8	Down
Dio3os	-3.182353352	9.20E-08	12	Down
Gm10101	-2.222286142	0.041938124	14	Down
Gm20684	-2.077536216	0.004478741	7	Down
Gm26684	-2.033512179	0.001023381	15	Down
B230354K17Rik	-1.792497952	5.82E-15	17	Down
9330162012Rik	-1.735574655	0.000517791	13	Down
Gm48768	-1.583498759	1.83E-05	10	Down
Gm5144	-1.414598651	0.027253129	15	Down
9530018F02Rik	-1.229569045	0.013593777	12	Down
BC048644	4.314735766	0.022372629	8	Up
E230013L22Rik	3.683997295	9.95E-23	8	Up
Gm45418	2.815829385	0.000612766	8	Up
Gm16675	2.766108993	8.62E-13	8	Up
D430020J02Rik	2.077847252	0.001131056	12	Up
4933412E12Rik	1.880429223	6.73E-13	10	Up
Haglr	1.750425984	0.004844512	2	Up
Gm26839	1.533171542	0.035142113	12	Up
2310014F06Rik	1.265258073	1.86E-07	7	Up
