# Supplementary material for: Transcriptomics Changes in the Peritoneum of Mice with Lipopolysaccharide-Induced Peritonitis
Source: Int J Mol Sci. 2021 Nov 30;22(23):13008. doi: 10.3390/ijms222313008 (PMC8657704; doi:10.3390/ijms222313008)
Supplement: Supplementary file 1 [file ijms-22-13008-s001.zip › ijms-1473149-supplementary/Supplementary_5_Table S4. Details of DEGs-pseudogene genes (LPS vs. Ctrl).pdf]

International Journal of *Molecular Sciences*

MDPI

Table S4. Details of DEGs-pseudogene genes (LPS vs. Ctrl)

Gene_symbol	log2FoldChange	Padj	Gene_chrosome	Change
Hmgb1-ps8	-6.041328811	8.25E-10	10	Down
Gm21013	-5.30528402	5.10E-06	9	Down
Alms1-ps1	-4.577788833	2.07E-05	6	Down
Adh6-ps1	-4.22846595	0.002430004	3	Down
Mrgprb11-ps	-4.147725344	9.01E-05	7	Down
Nxpe1-ps	-4.094886957	1.70E-05	9	Down
Gm8097	-3.957692624	0.014557895	X	Down
Gm26956	-3.852471539	0.041030197	13	Down
Gm47062	-3.759874803	0.016417816	9	Down
BC064078	-3.457558325	3.17E-12	6	Down
Gm17229	-3.384970565	0.007912595	10	Down
Alms1-ps2	-3.165682875	0.024407118	6	Down
Gm5454	-3.022705584	5.05E-05	13	Down
Gm47099	-2.94318294	0.036761143	10	Down
Gm7117	-2.858351905	0.000129616	X	Down
Gm3325	-2.855948576	9.65E-05	13	Down
Rpl19-ps3	-2.826345172	0.025735582	14	Down
Gm4804	-2.794321434	0.041900244	12	Down
Gm13688	-2.755438112	0.000767593	2	Down
Gm5575	-2.74210821	0.002818996	5	Down
Cyp4f41-ps	-2.629797751	0.033053879	17	Down
Rpl26-ps6	-2.611828117	0.010009791	9	Down
Gm49331	-2.574834268	4.90E-10	12	Down
Gm9387	-2.552258842	0.027028778	3	Down
Gm15422	-2.549482722	0.009403966	14	Down
Gm6723	-2.51628411	0.030476641	15	Down
Gpr137b-ps	-2.384015129	3.57E-27	13	Down
Zkscan7	-2.359435156	8.84E-05	9	Down
Gm36298	-2.341998847	1.04E-07	13	Down
Gm7240	-2.311202989	2.46E-05	13	Down
AC159187.1	8.454911655	4.51E-19	17	Up
Cxcl11	7.708606602	7.27E-57	5	Up
Gm5970	7.121312845	4.30E-53	18	Up
B230303A05Rik	6.143601795	1.76E-08	13	Up
Mx2	6.023557746	3.10E-91	16	Up
I830127L07Rik	5.854147176	1.42E-09	15	Up
Gm18445	5.795681939	2.35E-21	1	Up
CT025731.2	5.791769206	2.81E-08	17	Up
Gm12250	5.749528679	3.43E-44	11	Up
Ccl19-ps3	5.615045362	1.31E-10	4	Up
Gm6524	5.473606307	3.81E-06	8	Up
Serpina3h	5.34676281	1.95E-35	12	Up

Gm8818	5.231636028	1.81E-11	1	Up
Gm5300	5.079733204	0.000591239	6	Up
Gm4240	4.907053932	7.00E-18	14	Up
Gm12407	4.844237388	2.24E-23	4	Up
Gm18853	4.842158327	1.34E-09	7	Up
Gm19121	4.716212944	6.55E-05	9	Up
Gm6610	4.609484729	0.000325266	15	Up
Gm18752	4.586042844	8.20E-05	1	Up
Gbp11	4.575594673	3.53E-29	5	Up
Gm18246	4.554268479	0.005900806	12	Up
Gm6545	4.515643273	5.89E-05	19	Up
Olfr753-ps1	4.465132525	0.002422735	17	Up
Gm7582	4.361514088	0.000640602	1	Up
Gm29747	4.322534132	0.002654063	3	Up
Gm7115	4.316128711	4.23E-06	3	Up
Gm45471	4.297995055	0.007556784	7	Up
Rps8-ps3	4.197622772	0.013260064	6	Up
Gm17083	4.168379887	0.001659982	13	Up

Note: down-regulated and up-regulated 30 pseudogene genes were illustrated.
