# Supplementary material for: Transcriptomics Changes in the Peritoneum of Mice with Lipopolysaccharide-Induced Peritonitis
Source: Int J Mol Sci. 2021 Nov 30;22(23):13008. doi: 10.3390/ijms222313008 (PMC8657704; doi:10.3390/ijms222313008)
Supplement: Supplementary file 1 [file ijms-22-13008-s001.zip › ijms-1473149-supplementary/Supplementary_7_Table S6. Details of DEGs-processed_transcript genes (LPS vs. Ctrl).pdf]

International Journal of
Molecular Sciences

MDPI

Table S6. Details of DEGs-processed_transcript genes (LPS vs. Ctrl)

Gene_symbol	log2FoldChange	Padj	Gene_chrosome	Change
A830012C17Rik	-5.494889748	1.43E-07	4	Down
Gm10790	-5.344628148	3.38E-07	13	Down
2900041M22Rik	-4.662208239	3.15E-23	11	Down
4930447K03Rik	-4.116624326	0.026319463	13	Down
E330020D12Rik	-4.085889148	0.002109298	1	Down
Stamos	-3.77444065	0.000374721	2	Down
Gm48693	-3.686738909	0.036733667	12	Down
Gm44597	-3.658616854	0.010958169	6	Down
Gm28417	-3.589523648	0.001205842	1	Down
Gm12409	-3.541358122	0.042572093	4	Down
Gm11748	-3.491123361	0.012803227	11	Down
E230016K23Rik	-3.410766264	4.08E-09	11	Down
Hoxa11os	-3.240255997	0.005533673	6	Down
9130017K11Rik	-3.09447008	0.029266441	11	Down
B130024G19Rik	-2.986236206	2.42E-05	7	Down
2010001K21Rik	-2.947965053	0.000888262	13	Down
Gm14207	-2.854684802	2.44E-06	2	Down
3010001F23Rik	-2.854373827	0.024754894	X	Down
Ptprv	-2.851399035	0.01317413	1	Down
Trerf1	-2.812112011	7.74E-21	17	Down
Gm20492	-2.793731935	0.013565711	10	Down
Gm20743	-2.785360434	6.70E-10	1	Down
Gm16244	-2.773621202	0.020023542	19	Down
BC028777	-2.700196334	0.035627523	17	Down
Gm10044	-2.660165142	0.01226064	14	Down
Gm34121	-2.622806873	0.01731764	7	Down
Gm6556	-2.588659775	0.012562106	13	Down
2010016I18Rik	-2.474720755	0.000525629	3	Down
Foxd2os	-2.400622612	1.00E-05	4	Down
A930006K02Rik	-2.268151381	0.001841466	16	Down
1700011B04Rik	5.809288332	2.72E-08	13	Up
Gm12339	4.873362917	0.0010318	11	Up
Gm19744	4.464919746	1.62E-05	8	Up
AC154478.1	4.032025145	2.10E-08	16	Up
Gm14636	3.995409809	0.000650841	X	Up
Gm12503	3.491226347	0.030172497	4	Up
Anxa13	3.418415518	5.22E-06	15	Up
Gm26672	3.126307586	0.023690583	18	Up
G530011O06Rik	2.985307527	8.97E-09	X	Up
Gm28496	2.858597423	0.017838764	7	Up
4931431B13Rik	2.716660535	0.006761584	7	Up
Gm20470	2.692481411	0.032389912	10	Up

Gm20559	2.52917493	2.04E-17	6	Up
AW047730	2.279442314	0.044198316	3	Up
4930597O21Rik	2.224261549	0.019133588	6	Up
Gm16096	2.195637479	0.000409774	9	Up
Gm43305	2.186726372	1.15E-07	14	Up
C4a	2.170682597	3.34E-06	17	Up
Gm15832	2.041754693	7.50E-09	1	Up
2410006H16Rik	2.039786967	2.55E-20	11	Up
Snhg15	2.027441339	3.56E-36	11	Up
9530080O11Rik	1.910473244	0.001176049	4	Up
Snhg17	1.844571431	3.83E-21	2	Up
Snhg5	1.831363478	4.70E-15	9	Up
Snhg1	1.794337871	1.01E-22	19	Up
F830208F22Rik	1.788427737	0.000611371	18	Up
Gm15764	1.641752121	0.014168194	16	Up
1700101I11Rik	1.574454516	0.000302766	6	Up
Gm17494	1.563099677	0.048497313	3	Up
Dancr	1.548971836	0.000184145	5	Up

Note: down-regulated and up-regulated 30 processed_transcript genes were demonstrated.
