# Supplementary material for: Transcriptomics Changes in the Peritoneum of Mice with Lipopolysaccharide-Induced Peritonitis
Source: Int J Mol Sci. 2021 Nov 30;22(23):13008. doi: 10.3390/ijms222313008 (PMC8657704; doi:10.3390/ijms222313008)
Supplement: Supplementary file 1 [file ijms-22-13008-s001.zip › ijms-1473149-supplementary/Supplementary_8_Table S7. Details of DEGs-processed_transcript genes (TAK vs. LPS).pdf]

International Journal of
Molecular Sciences

Table S7. Details of DEGs-processed_transcript genes (TAK vs. LPS)

Gene_symbol	log2FoldChange	Padj	Gene_chrosome	Change
Mir99ahg	2.05934764	0.032118609	16	Up
Gm17066	1.638658503	0.026443359	14	Up
