# Supplementary material for: Transcriptomics Changes in the Peritoneum of Mice with Lipopolysaccharide-Induced Peritonitis
Source: Int J Mol Sci. 2021 Nov 30;22(23):13008. doi: 10.3390/ijms222313008 (PMC8657704; doi:10.3390/ijms222313008)
Supplement: Supplementary file 1 [file ijms-22-13008-s001.zip › ijms-1473149-supplementary/Supplementary_file.pdf]

International Journal of
Molecular Sciences

MDPI

Supplementary Materials

Transcriptomics changes in the peritoneum of mice with lipo-polysaccharide-induced peritonitis

Shaoguang Liu [1], Shaotong Zhang [2], Yulong Sun [3], Wence Zhou [1*]

Affiliations:

1 The First Clinical Medical College, Lanzhou University, Lanzhou, Gansu Province, 730000, China; liushaoguang@gszy.edu.cn (S.G.L); zhouwc@lzu.edu.cn (W.C.Z).

2 The Clinical Medical College, Ningxia Medical University, Yinchuan, Ningxia Hui Autonomous Region, 750004, China; zhangshaotong@nxmu.edu.cn (S.T.Z).

3 Key Laboratory for Space Biosciences & Biotechnology, Institute of Special Environmental Biophysics, School of Life Sciences, Northwestern Polytechnical University, Xi'an, Shaanxi Province, 710072, China; yulongsun@nwpu.edu.cn (Y.L.S).

* Correspondence: zhouwc@lzu.edu.cn; Tel.: +86 0931 8356022

List of contents:

1. Overall Summary

2. Results

1. Overall Summary

In the present study, the transcriptomics profiles of mouse peritoneum with LPS-mediated peritonitis were investigated with a series of comprehensive approaches such as transcriptomics, bioinformatics, and molecular simulation (Figure 1). 1) The es-tablishment of the model. An LPS-stimulated mouse peritonitis model was established. 2) Anti-peritonitis activity of TLR4 inhibitor TAK 242. The effect of TAK 242 on the inflam-matory cytokines in the peritoneal lavage fluid of mice was examined by the ELISA test. 3) Transcriptome sequencing of mouse peritoneal tissue. The peritoneal tissues of three groups of mice (Control group, LPS group, LPS+TAK 242 group) (n = 6) were isolated and subjected to RNA-seq. 4) Bioinformatics analysis. A series of bioinformatics analyses were performed, including differentially expressed genes (DEGs), enrichment pathways, pro-tein-protein interaction, transcription factor pathways, and hub genes. 5) Molecular dy-namics analysis. The three-dimensional structure of hub proteins corresponding to hub genes was obtained by using homology modeling and molecular dynamics (300 ns). 6) Molecular docking. The molecular docking between TAK 242 and hub proteins was car-ried out to investigate the possible binding modes. Overall, our data provide clues for gene expression in mouse models of LPS-mediated peritonitis, which will be helpful for ex-ploring the mechanism of LPS-induced peritonitis in mice.

2. Results

2.1. LPS-induced Peritonitis in Mice

2.2. Screening of DEGs

2.3. Enrichment Pathway of DEGs-KEGG

2.4. The Enrichment Pathway of DEGs-Metascape

2.5. Enrichment Pathway of DEGs-PANTHER

2.6. The Enrichment Pathway of DEGs-Cluego

2.7. Transcriptional Factors Tied to DEGs

2.8. Protein-protein Interaction (PPI) Network of DEGs

2.9. Identification of Hub Genes

2.10. Modeling of Hub Proteins

2.11. Molecular Dynamics Simulation of Hub Proteins

2.12. TAK 242-hub Protein Docking
